# Corticosterone-linked microglial activity underpins sexually dimorphic neuroplasticity after ketamine anesthesia

**DOI:** 10.1126/sciadv.adz6517

**Published:** 2026-07-31

**Authors:** Alessandro Venturino, MohammadAmin Alamalhoda, Thomas Negrello, Kelly Jin, Cindy T. J. van Velthoven, Ryan John A. Cubero, Jake Yeung, Peter Koppensteiner, Bosiljka Tasic, Sandra Siegert

**Affiliations:** ^1^ Institute of Science and Technology Austria (ISTA), Am Campus 1, 3400 Klosterneuburg, Austria.; ^2^ Allen Institute, Brain Science, 615 Westlake Ave. N, Seattle, WA 90109, USA.

## Abstract

Anesthesia recovery is critical for resuming normal physiological and neuronal functions; however, the mechanisms involved remain elusive. Here, we identify a female-selective corticosterone-mediated microglia-neuron interaction during ketamine anesthesia recovery, absent in males. This microglia-neuron interaction induces plastic and functional neuronal changes, as evidenced by increased mEPSC frequency, which was occluded upon microglia depletion. We showed that this process is driven through up-regulation of the stress-responsive co-chaperone *Fkbp5* mRNA and its protein, Fkbp51, in female microglia. *Fkbp5*/Fkbp51 is a key intermediary in a corticosteroid-induced stress response, and its involvement points toward a critical interface between endocrine signaling and microglia. To counteract the observed ketamine anesthesia-mediated increase in blood corticosterone during recovery, we removed the primary source of corticosterone by adrenalectomy. Close microglia-neuron interaction was reduced and increased again following corticosterone injection. Our findings identify a sex-specific microglia-mediated mechanism of neuronal plasticity during anesthesia recovery, driven by corticosterone, thereby enhancing our understanding of sex differences in brain function.

## Teaser

Immune cells in the brain of females reshape neuronal connections during recovery from anesthesia.

## INTRODUCTION

Recovery from anesthesia is a fundamental yet complex process critical for resuming physiological functions. Ketamine distinguishes itself from other anesthetics by its unique pharmacological properties as an *N*-methyl-d-aspartate (NMDA) receptor antagonist, which preferentially targets GABAergic inhibitory interneurons (*1*, *2*). It has generally been considered to comply with the principles of general anesthesia, which require a drug-inducible, fully reversible change in consciousness and cognition (*3*). However, repeated exposure to ketamine anesthesia reinstates juvenile-like plasticity in the primary visual cortex, mediated through alterations in the extracellular perineuronal nets (*4*). Moreover, ketamine anesthesia induces mild anxiety behavior phenotypes, interestingly, only in females (*5*), suggesting inherent sex differences in anesthesia recovery with neuronal consequences that extend beyond the immediate sex-dependent metabolic processing described for low-dose ketamine (*6*).

Ketamine, across different dosages, affects microglia (*4*, *7*–*9*), which are embedded within the neuronal network (*10*). Locally, microglia influence the synaptic machinery and neuronal firing properties by responding to environmental changes (*8*, *11*–*14*). Sex differences have been reported to influence microglial function (*7*, *15*–*18*), yet, as immune-associated cells, microglia remain underexplored in the emerging fields of sex differences in immune responses (*19*, *20*) and neuronal activity (*21*, *22*).

Here, we investigate whether sex-specific microglia-neuron dynamics occur in a murine model of ketamine-induced anesthesia. Only female mice showed a pronounced interaction between microglia and neurons during recovery, which was followed by increased synaptic density and enhanced neuronal firing. Mechanistically, we found that female microglia selectively up-regulated the co-chaperone *Fkbp5*/FK506-binding protein 51 (Fkbp51), which is a key intermediary in the corticosteroid-induced stress response (*23*–*25*). The selective hypothalamic activation and elevated blood plasma corticosterone levels during the recovery phase in females shape the microglia-neuron interactions, highlighting a link between the endocrine and the brain-immune axes.

## RESULTS

### Microglia-enabled neuronal network adaptation upon KXA recovery only in females

**Fig. 1.**
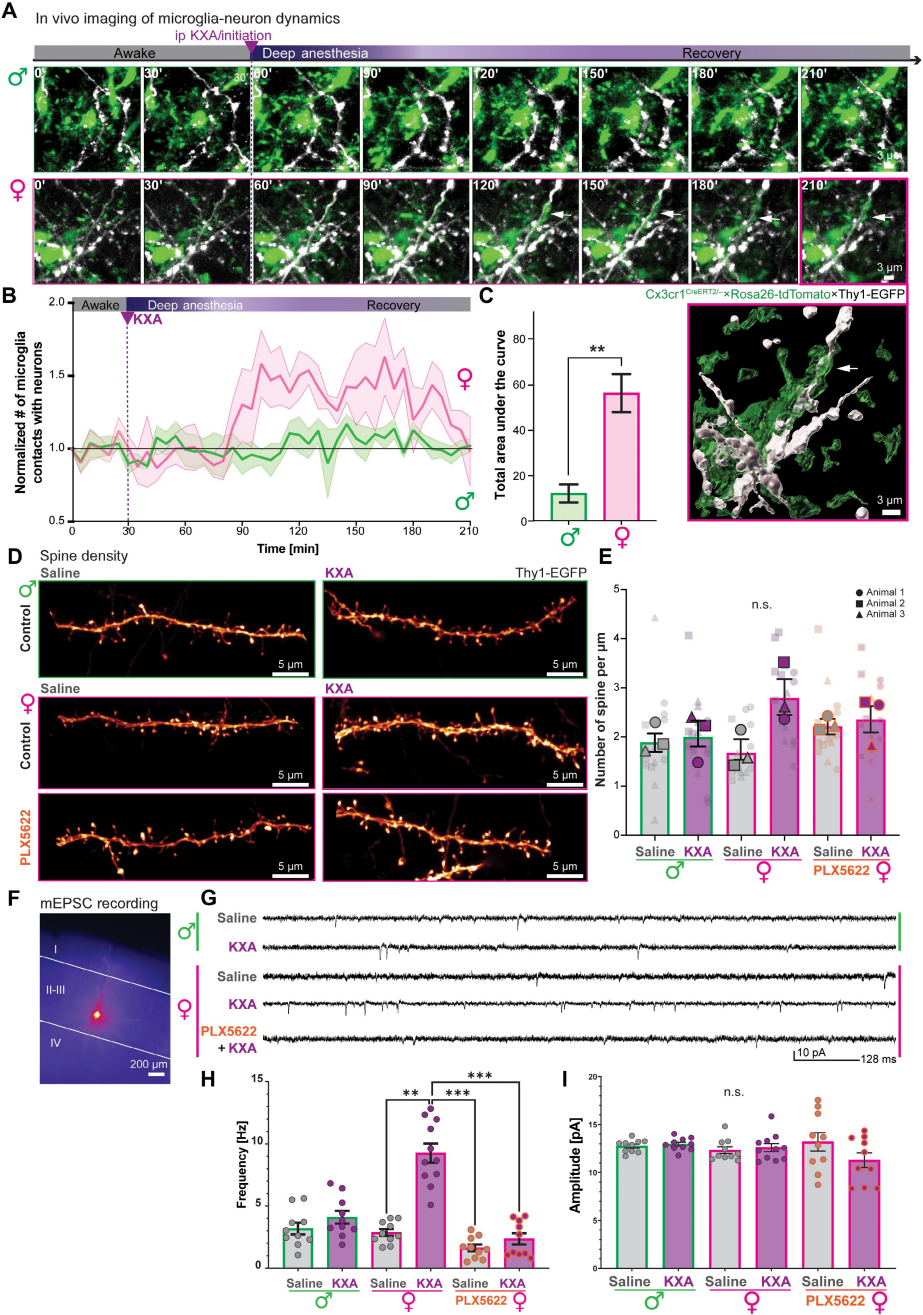
Female microglia interact with neurons, promoting spinogenesis and plasticity upon ketamine recovery. (**A** to **C**) In vivo two-photon imaging in the primary visual cortex (VISp) through a cranial window of Cx3cr1^CreERT2/−^×Ai9×Thy1-EGFP mice of both sexes spanning the phases of awake, deep anesthesia, and recovery after KXA administration. (A) Sequential snapshots of microglia (green) and excitatory neurons (white). Top, males; bottom, females; white arrow, prolonged microglia-dendrite contact; magenta frame, 3D surface rendering of the 210-min frame in females. Scale bars, 3 μm. (B) Normalized number of microglia and Thy1-EGFP neuronal process contacts over time in males (green) and females (magenta) as mean ± SEM confidence band. Dashed green line, KXA injection. Five animals per condition. (C) Bar chart of the mean total area under the curves in (B). Mean ± SEM of five animals per condition. Unpaired *t* test with Welch’s correction, **P < 0.01. (**D** and **E**) Spine density quantification in VISp, layer II/III in Thy1-EGFP mice 4 hours after saline or KXA injection. (D) Representative super-resolution images of dendritic processes. Top, males; middle, females; bottom, 1.5-week PLX5622-treated females to deplete microglia. Scale bars, 5 μm. (E) Bar chart of the mean number of spines per micrometer with ±SEM. Five dendrites per animal. Symbols, different animals. Three animals per condition. One-way nested analysis of variance (ANOVA), not significant (n.s.) *P* > 0.05. (**F** to **I**) mEPSC (miniature excitatory postsynaptic currents) recording from layer II/III VISp pyramidal neurons, 4 hours after saline or KXA injection in males, females, or microglia-depleted females (PLX5622). (F) Representative epifluorescence image of a biocytin-filled pyramidal neuron after mEPSC recording. Scale bar, 200 μm. (G) Representative recorded mEPSC example traces. [(H) and (I)] Bar charts of mean mEPSC frequency (H) and amplitude (I) with ±SEM. Dot, recorded neuron. Two to four cells per animal. Three animals per condition. Kruskal-Wallis with selected Dunn’s multiple comparisons post hoc test, **P < 0.01, ***P < 0.001, and *P* > 0.05 (n.s). ip, intraperitoneal.

Anesthesia is typically divided into the initiation, deep anesthesia maintenance, and recovery stages (Fig. 1A). We administered a single dose of ketamine-xylazine-acepromazine (KXA) anesthesia to C57BL6/J mice, euthanized the animals 4 hours later, and performed immunostaining for Iba1 to identify microglia, the endosomal-lysosomal marker CD68 as a proxy for microglial reactivity, and Vglut2 to delineate the cortical layers (fig. S1, A and B). We focused our subsequent analysis on the primary visual cortex (VISp) because we have previously shown that repeated exposure to ketamine anesthesia reinstated juvenile-like plasticity (*4*). Unexpectedly, when we analyzed CD68 volume in microglia, we found that CD68 was up-regulated only in female microglia across cortical layers II/III, IV, and V/VI (fig. S1, A to C). In parallel, the microglia three-dimensional (3D) morphology indicated a shift in the latent space of the KXA female population away from the saline condition (fig. S1D), which we analyzed with an adapted morphOMICs pipeline (*7*) without bootstrapping. We confirmed that this shift in the density distribution was significant for microglia in II-VI (fig. S2 and Materials and Methods). In contrast, the microglial morphologies in males remained intermingled between the KXA and the saline condition (fig. S1E). In addition to the CD68 increase in females after KXA, CD68 relocated from the soma to the tips of the microglia processes (fig. S3, A and B) and also positively labeled for the perineuronal net (PNN) staining *Wisteria floribunda agglutinin* (WFA; fig. S3, C to F) (*4*). WFA selectively binds to the *N*-acetyl-galactosamine group of the chondroitin sulfate proteoglycans, which is part of the perineuronal nets, among others, in the cortical layer IV (*26*). All these observations suggest that a single round of KXA anesthesia affects female microglia differently than it does for male microglia. Notably, CD68 up-regulation in female microglia was not exclusive to the VISp and also occurred in the olfactory bulb, frontal cortex, dentate gyrus, and substantia nigra, whereas the cerebellum did not show this effect (fig. S4A). CD68 was then down-regulated again after 48 hours (fig. S4B), supporting a broad systemic yet specific effect of KXA anesthesia after 4 hours. A 40-min isoflurane anesthesia and analyses of the brain 4 hours after induction did not replicate this phenotype (fig. S5).

To verify the sex-specific KXA effect and identify the stage of anesthesia at which female microglia start to respond, we imaged microglial dynamics in vivo in the VISp. We performed a cranial window surgery at the Cx3cr1^CreERT2/het^×Ai9-tdTomato mouse model, crossed with Thy1–enhanced green fluorescent protein (EGFP), to visualize microglia and the sparsely labeled pyramidal neuronal processes reaching cortical layers I and II, respectively. During the 30-min baseline recording, microglial processes infrequently contacted neuronal processes and dendritic spines, and quantification of the nonnormalized baseline recordings revealed no significant differences between groups (fig. S6). After the intraperitoneal injection of KXA, microglial contacts remained similar to baseline for ∼60 min during anesthesia maintenance (Fig. 1, A and B). One hour following the single dosage of KXA injection, female microglia established significantly more contacts with the dendritic processes and spines at the group level (Fig. 1C, fig. S6, and movies S1 and S2). This interaction gradually diminished over the next 3 hours of in vivo recording, as the animal slowly awoke. Although a subset of male animals also displayed transient increases in microglia-neuron interactions, these responses were heterogeneous and did not produce a significant group-level effect.

To determine whether the close interaction of microglial processes with pyramidal cells correlates with changes in dendritic processes (*14*, *27*, *28*), we quantified spine density in the second-order dendrites 4 hours after KXA using super-resolution imaging (Fig. 1D). Females subjected to KXA exhibited a larger observed effect size in spine density relative to saline conditions (Hedges *g*: 1.5). However, these effects did not reach statistical significance after a nested analysis (Fig. 1E). We next examined spontaneous miniature excitatory postsynaptic currents (mEPSCs) from layer II/III pyramidal cells in slice recordings at the same period (Fig. 1, F and G). The mEPSC frequency increased significantly only in females (Fig. 1H), while the amplitude remained unchanged across both sexes (Fig. 1I). To establish the relevance of microglia in this process, we provided chow containing the colony stimulating factor 1 receptor (CSF1R) blocker PLX5622 to mice 1.5 weeks before the KXA experiment. This treatment achieved ∼80% microglial depletion (fig. S7). It abolished the effects of increased spine numbers in females after KXA (*g* = 0.18; Fig. 1, D and E) as well as the increase in mEPSC frequency (Fig. 1, G to I), supporting the notion of female-selective microglia-enabled neuroplasticity.

### KXA affects genes associated with the glucocorticoid pathway

**Fig. 2.**
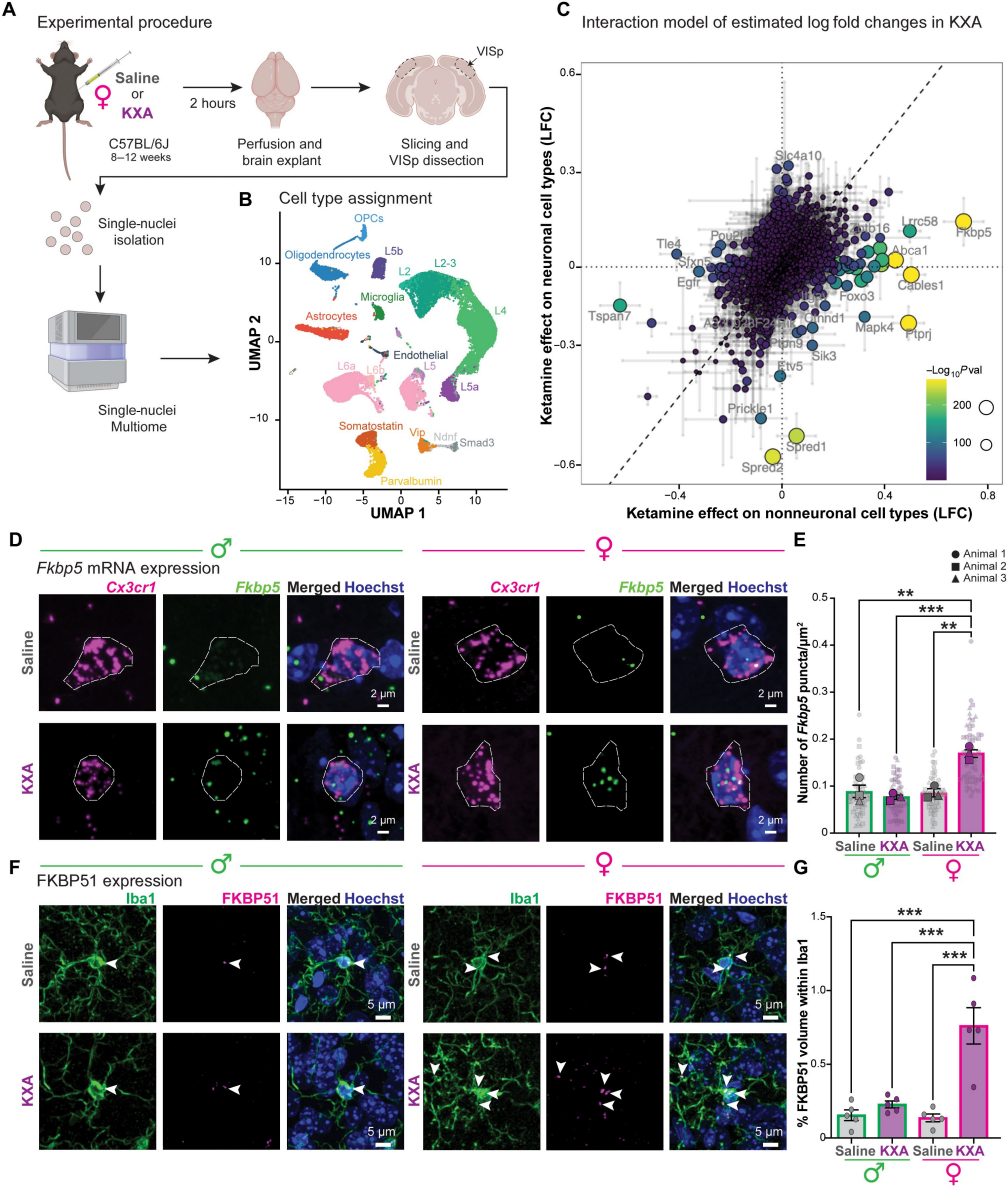
Microglia up-regulate the stress mediator *Fkbp5*/Fkbp51. (**A**) Experimental strategy for single-nuclei multiome sequencing. Primary visual cortex (VISp) of female mice microdissected 2 hours after saline or KXA treatment. Figure created with BioRender.com. (**B**) Uniform Manifold Approximation and Projection (UMAP) visualization of single-nucleus transcriptomes and cell type assignment. (**C**) Log fold change (LFC) estimates from KXA treatment on nonneuronal (astrocytes and microglia) versus neuronal cell types. Size and yellow intensity of points are proportional to the log_10_(*P* value) from ANOVA for interaction between ketamine effect and astrocytes/microglia cell type (see Materials and Methods). Error bars: 90% confidence intervals inferred from the KXA effect on nonneuronal or neuronal cell types. Genes along the dashed line have equal LFC and zero interaction effect. (**D** and **E**) Fluorescence in situ hybridization for male and female mice 2 hours after saline or KXA injection in VISp. (**D**) Example high-magnification images of mRNA probes against *Fkbp5* (green) and *Cx3cr1* (magenta, microglia), counterstained with the nuclei dye Hoechst (blue), which provides the nucleus contour (white dashed line). Scale bars, 2 μm. (**E**) Bar chart of the mean *Fkbp5* mRNA puncta within the Hoechst contour with SEM. Each dot represents one microglial contour, 20 cells per animal, three animals per condition. One-way nested ANOVA with selected Tukey’s multiple comparisons post hoc test, **P < 0.01 and ***P < 0.001. (**F** and **G**) Fkbp51 protein expression 4 hours after saline or KXA injection in the VISp, layers III-V of males and females. (F) Example immunofluorescence images for Iba1 (green) and Fkbp51 (magenta), counterstained with the nuclei dye Hoechst (blue). White arrowheads, Fkbp51 localization within Iba1^+^ microglia. Scale bars, 5 μm. (G) Bar chart showing the mean percentage of Fkbp51 volume in microglia, with ±SEM. Each dot represents an animal, five animals per condition. Two-way ANOVA with selected Tukey’s multiple comparisons post hoc test, ***P < 0.001.

To unbiasedly screen for pathways associated with the microglia-mediated response, we performed single-nucleus multiome sequencing of the entire cell population in female VISp 2 hours after KXA induction (Fig. 2A). The total dataset consisted of 36,701 cells after quality control and removal of low-quality cells (fig. S8, A to D). After batch-corrected normalization, we assigned the clusters to individual cell types (Fig. 2B and fig. S8H). The overall effect of KXA across the entire population did not produce a prominent cluster or shift among the cell populations. Furthermore, microglia and astrocytes were distributed similarly across the conditions, indicating no cell loss or proliferation. Next, we identified the 2000 most differentially regulated genes in the global transcriptome. We developed an interaction model to estimate log fold changes in KXA treatment effects on the nonneuronal astrocyte-microglia population relative to neuronal cell types, using a linear mixed model (LMM). Most genes had small effect sizes and remained unaffected by KXA (Fig. 2C). Specifically, in neurons, only *Spred1* and *Spred2* were down-regulated, which are associated with the mitogen-activated protein (MAP) kinase cascade (*29*, *30*). In contrast, nonneuronal cells showed significant up-regulation of *Abca1*, *Cables1*, *Ptprj*, and *Fkbp5*. A common theme among these genes is their relationship to glucocorticoids, either in their production from cholesterol (*Abca1*) (*31*, *32*) and in lipid metabolism (*Ptprj*) (*33*) or in their regulation (*Cables1* and *Fkbp5*) (*34*, *35*). Specifically, *Fkbp5* and its protein, the Fkbp51, are essential stress-response regulators (*23*–*25*) that tightly coregulate glucocorticoid receptor expression and a cell’s sensitivity to detect the stress hormone corticosterone (*24*, *25*, *35*, *36*). On the basis of these findings, we focused our subsequent analysis on *Fkbp5*/Fkbp51, which we validated using orthogonal histological, pharmacological, and genetic approaches.

To verify the selective up-regulation of *Fkbp5* mRNA in female KXA microglia, we performed fluorescence in situ hybridization and quantified the number of *Fkbp5* puncta within the Hoechst nuclei contour using cell type–specific probes in KXA- and saline-treated tissue from both sexes. Only female *Cx3cr1^+^* microglia exposed to KXA showed a significant increase in *Fkbp5* puncta. This effect did not occur in males (Fig. 2, D and E). Furthermore, the KXA-mediated up-regulation was specific to microglia. In S100β^+^ astrocytes, *Fkbp5* puncta were reduced in males, whereas in females, the effect size was medium (*g =* 0.65). In contrast, neuronal nuclei (NeuN)^+^ neurons showed no significant differences between the saline and KXA conditions, with a small effect size of 0.19 and 0.21 in males and females, respectively (fig. S9).

Last, we evaluated whether *Fkbp5* up-regulation also translated into increased Fkbp51 protein level. Like *Fkbp5*, only female microglia up-regulated Fkbp51 4 hours after KXA exposure (Fig. 2, F and G), suggesting that *Fkbp5*/Fkbp51 contributes to the female microglial response during KXA recovery.

### *Fkbp5* links to the female microglia-enabled effect on neuronal plasticity

**Fig. 3.**
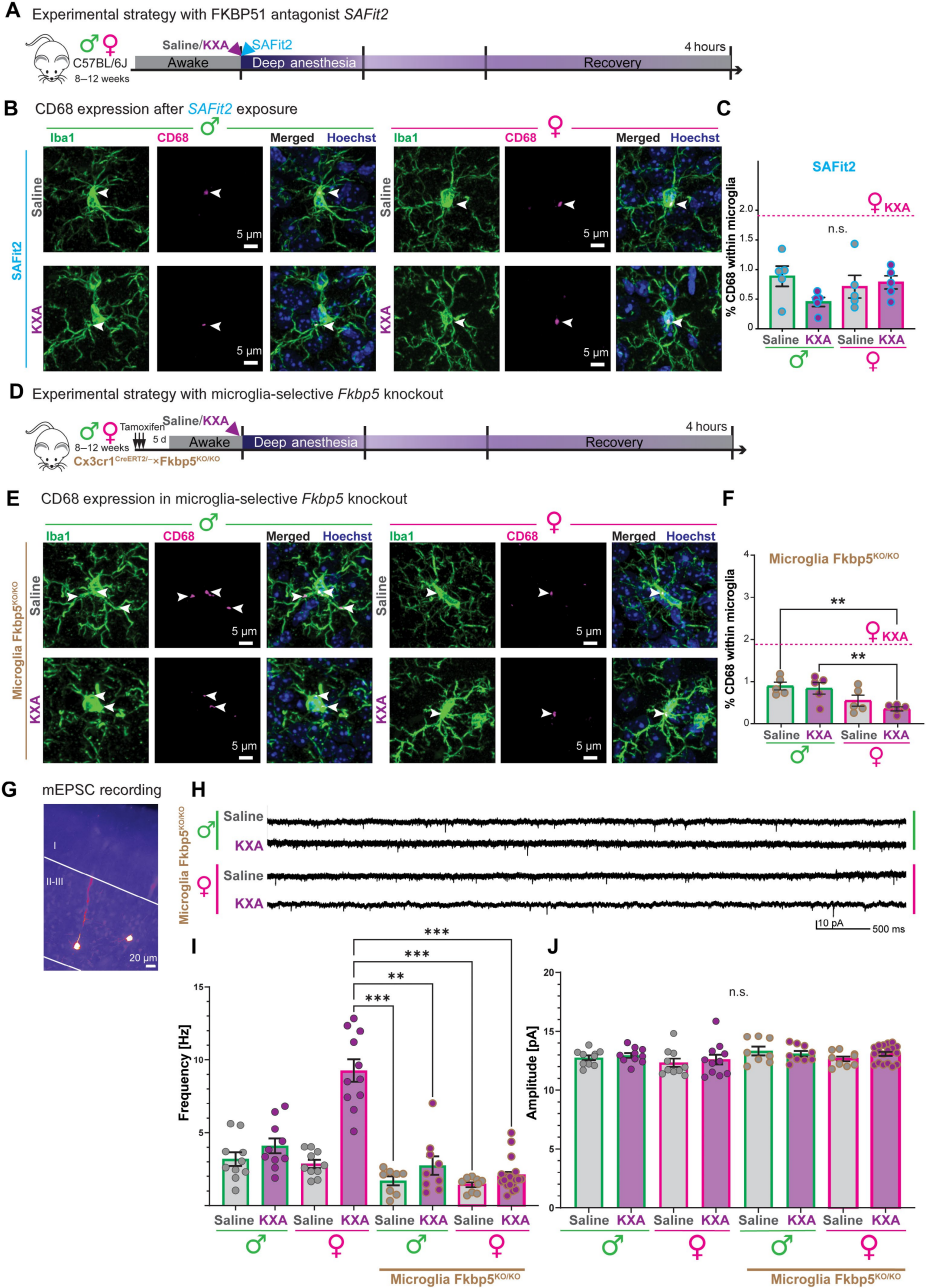
SAFit2 treatment or microglia-specific *Fkbp5* knockout prevents female microglia response and neuronal plasticity. (**A** to **C**) Experimental strategy for antagonizing Fkbp51 with SAFit2 after injection of saline or KXA. (B and C) CD68 quantification in VISp, layer III-V, males and females, 4 hours after saline or KXA with SAFit2. (B) Representative immunostainings for Iba1 (green) and CD68 (magenta), counterstained with nuclei dye Hoechst (blue). Arrow, CD68 localization within microglia. Scale bars, 5 μm. (C) Bar chart of mean percentage of CD68 volume within microglia with ±SEM. Dot, one animal. Five animals per condition. Dashed line, reference value of female KXA without SAFit2 from fig. S1C. Two-way ANOVA. *P* > 0.05 (n.s.). (**D** to **J**) Consequences of microglia-selective *Fkbp5*-knockout experiment using a tamoxifen-inducible *Cx3cr1*^CreERT2/−^×*Fkbp5*^KO/KO^ reporter mouse line (microglia *Fkbp5*^KO/KO^). (D) Experimental strategy. Three consecutive tamoxifen injections, starting 8 days before procedure. (E and F) CD68 quantification in VISp, layer III-V, males and females, 4 hours after saline or KXA. (E) Representative images of immunostainings for Iba1 (green) and CD68 (magenta), counterstained with the nuclei dye Hoechst (blue). Arrow, CD68 localization within microglia. Scale bars, 5 μm. (F) Bar chart of the mean percentage of CD68 volume within microglia with ±SEM. Dot, one animal. Five animals per condition. Dashed line, reference value of female KXA in C57BL/6J from fig. S1C. Two-way ANOVA with selected Tukey’s multiple comparisons post hoc test, **P < 0.01. [(G) to (J)] mEPSC recording from layer II/III VISp pyramidal neurons, 4 hours after saline or KXA injection, males or females. (G) Representative epifluorescence image of biocytin-filled pyramidal neuron after mEPSC recording. Scale bar, 20 μm. (H) Representative recorded mEPSC example traces. [(I) and (J)] Bar charts of mean mEPSC frequency (I) and amplitude (J) with ±SEM. Dot, recorded neuron. Two to four cells per animal. Three animals per condition. Kruskal-Wallis with selected Dunn’s multiple comparisons post hoc test. **P < 0.01, ***P < 0.001, and *P* > 0.05 (n.s.). d, days.

*Fkbp5*/Fkbp51 is an interesting drug target for pharmaceutical strategies due to its predicted gene-environment interactions at the human *FKBP5* locus in various mental disorders (*25*, *37*). Selective antagonists of the Fkbp51 by induced fit (including SAFit2) have been developed with a high binding affinity for Fkbp51 (*37*, *38*). We injected SAFit2 with either KXA or saline and analyzed the overall effect on CD68 expression in microglia 4 hours later (Fig. 3A). Inhibiting Fkbp51 prevented KXA-mediated CD68 up-regulation (Fig. 3, B and C) and PNN accumulation within the microglial CD68 compartments (fig. S10, A to C). SAFit2 injected with saline did not directly affect microglia morphology. In contrast, SAFit2 and KXA treatment partially mitigated the morphological shift observed in KXA female microglia across cortical layers (fig. S11, A to C).

Since SAFit2 affects all cells that express Fkbp51, including neurons and astrocytes (fig. S9), we chose to examine a microglia-selective *Fkbp5* knockout mouse line (*39*), *Cx3cr1*^CreERT2/het^×*Fkbp5*^flox/flox^, for KXA effects (Fig. 3D). After confirming the selective Fkbp51 loss in microglia following the tamoxifen-induced knockout (fig. S12, A to C), we verified the lack of KXA-mediated CD68 level increase (Fig. 3, E and F) and the containment of WFA staining (fig. S12, D and E), suggesting that the selective knockout of *Fkbp5* hindered the targeted microglial response upon KXA recovery. Notably, the microglial density remained unaffected, excluding macrophage infiltration (fig. S12, F and G). Last, to test effects on neuronal plasticity, we measured the frequency and amplitude of mEPSC in layer II/III pyramidal cells after saline or KXA exposure in both males and females (Fig. 3, G and H). We found that ketamine failed to induce a significant increase in the mEPSC frequency (Fig. 3I) in females and did not affect males. The amplitude remained unaltered (Fig. 3J). These results suggest an involvement of *Fkbp5*/Fkbp51 in the female-specific microglia response during ketamine recovery.

### Corticosterone as a driver of sex-specific microglia-mediated effects

*Fkbp5*/Fkbp51 up-regulation in female microglia during KXA recovery and the lack of a microglial response upon *Fkbp5* knockout and Fkbp51 inhibition suggest a link between the body periphery and neuronal network adaptation mediated via the endocrine steroid hormone corticosterone, the only glucocorticoid in mice (*40*). The hypothalamic paraventricular nucleus (PVN) is one of the key regions that regulates corticosterone levels by releasing corticotropin-releasing hormone (CRH). The anterior pituitary gland converts CRH into adrenocorticotropic hormone, which stimulates the adrenal glands to release corticosterone (*31*, *41*).

**Fig. 4.**
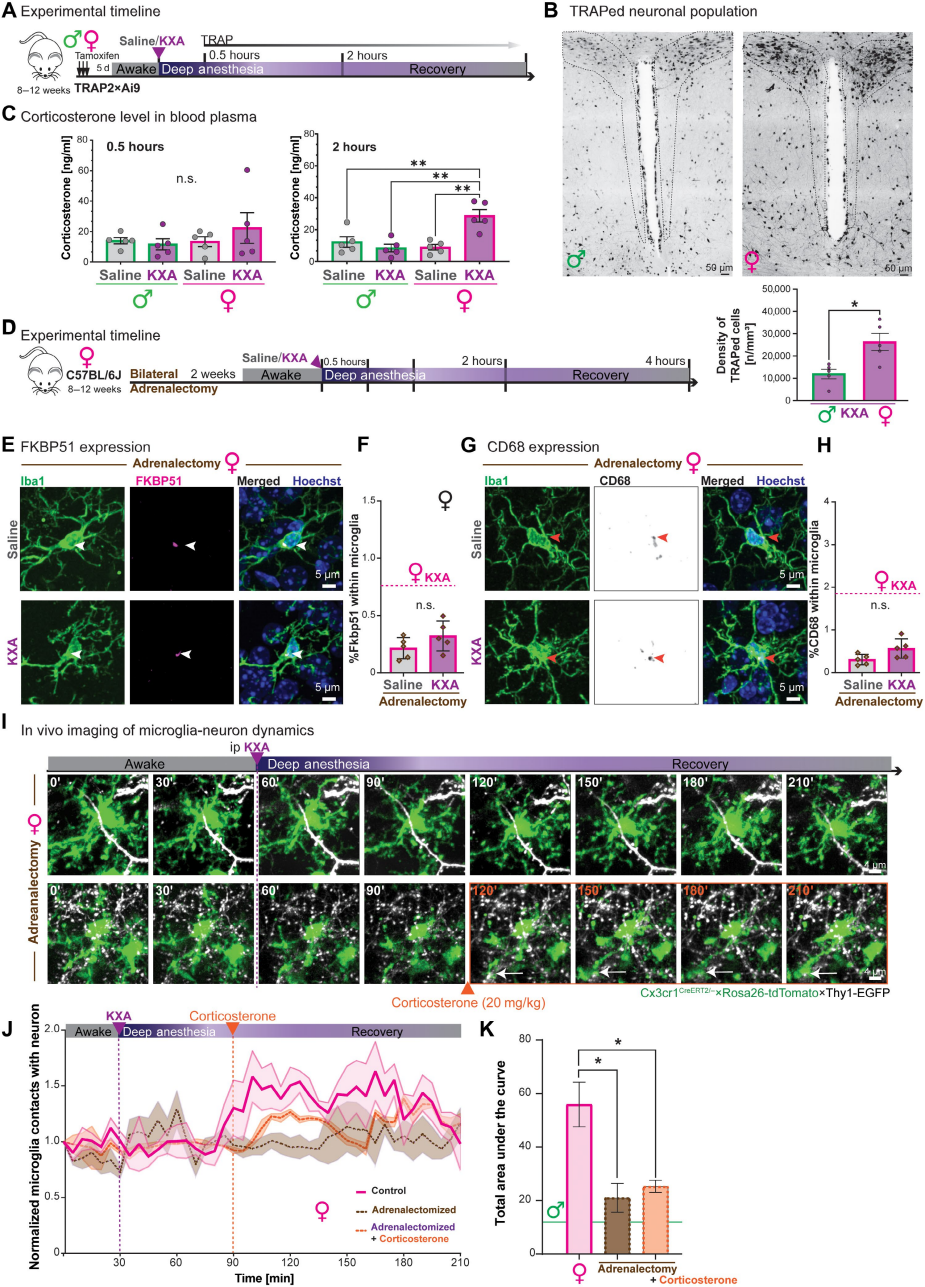
Ketamine-induced corticosterone modulates female microglia-neuron interaction. (**A**) Experimental timeline. (**B**) Representative images of targeted recombination in active populations (TRAP)ed neurons 30 min after KXA in PVN (dashed line, coronal); males (left), females (right). Scale bars, 50 μm. Bottom, bar chart of TRAPed neurons, mean density ± SEM. Dot, one animal. Five mice per condition. Unpaired *t* test with Welch’s correction, *P < 0.05. (**C**) Corticosterone level in blood plasma; males (green), females (magenta), either at 0.5 (left) or 2 hours (right). No repeated blood collection. Dot, one animal. Five mice per condition. Two-way ANOVA with selected Tukey’s multiple comparisons post hoc test, **P < 0.01. (**D** to **K**) Experimental timeline for bilateral adrenalectomy in females. (E to H) Immunostainings and quantification in VISp, layer III-V, 4 hours after saline or KXA. (E and G) Representative immunostainings for Iba1 (green), Fkbp51 (magenta, E), and CD68 (gray, G), counterstained with nuclei dye Hoechst (blue). Arrow, localization within microglia. Scale bars, 5 μm. (F and H) Bar chart of mean percentage ± SEM of Fkbp51 (F) and CD68 (H) volume within microglia. Dot, one animal. Five animals per condition. Dashed line, reference value of female KXA for Fkbp51 (Fig. 2G) and CD68 (fig. S1C). Welch’s *t* test. *P* > 0.05 (n.s.). (I to K) In vivo two-photon imaging in VISp of adrenalectomized *Cx3cr1*^CreERT2/−^×Ai9×Thy1-EGFP females. (I) Sequential snapshots; microglia (green); neurons (white). Top, adrenalectomized females; bottom, adrenalectomized females with intraperitoneal corticosterone injection (orange); white arrow, prolonged microglia-dendrite contact. Scale bars, 4 μm. (J) Normalized number of microglia and Thy1-EGFP contacts (magenta; see Fig. 1B), adrenalectomized females (purple, *n* = 3), and adrenalectomized females injected with corticosterone (orange, *n* = 3) as mean ± SEM confidence band. Dashed lines: KXA (green) and corticosterone (orange). (K) Bar chart of mean total area under curves with ±SEM from (J). Green line, reference value male from Fig. 1C. Mean ± SEM. Three to five animals per condition. Brown-Forsythe ANOVA test with Dunnett’s T3 multiple comparisons test, *P < 0.05.

To systematically establish the connection between corticosterone and the microglia-mediated effects, we used the targeted recombination in active population (TRAP) cFos mouse line (*42*), crossed with the Ai9 reporter line. We trapped neurons with 4-OH-tamoxifen, starting 30 min after KXA, to capture c-Fos–dependent neuronal activity during the recovery phase between both sexes (Fig. 4, A and B). We found a significantly higher number of trapped neurons in the female PVN than in males (Fig. 4B). To determine whether this translates into increased corticosterone levels, we collected blood plasma at 30 or 120 min after saline or KXA injection (Fig. 4A). After 30 min, corticosterone levels were comparable between saline- and KXA-injected groups, with no sex differences (Fig. 4C). After 120 min, only in females, corticosterone increased almost threefold after KXA, suggesting an intrinsic, sex-dependent response during the KXA recovery.

The adrenal glands are the primary source of corticosterone (*41*). We performed bilateral adrenalectomy to prevent the KXA-mediated rise in corticosterone (Fig. 4D). After the surgery, the animals were exposed to low doses of corticosterone in their drinking water to avoid altering circadian rhythms (*43*). To confirm that this experimental procedure prevented microglia response, we performed immunostaining for Fkbp51 and CD68 4 hours after KXA exposure. Those females exhibited significantly lower Fkbp51 and CD68 expression in microglia than non-adrenalectomized animals (Fig. 4, E to H, and fig. S13, A and B). Next, we used this model to link microglia-mediated Fkbp51 corticosterone to neuronal interaction. We performed cranial window surgery in *Cx3cr1*^CreERT2/het^×Ai9×Thy1-EGFP adrenalectomized females and imaged microglia-neuron interaction in vivo 2 weeks after the surgery. The baseline activity of microglial processes contacting neuronal processes was similar among adrenalectomized females (fig. S13C), and quantification of the non-normalized baseline recordings revealed no significant differences (fig. S6D). When we administered KXA, microglia did not significantly increase their interactions with dendritic processes (Fig. 4, I to K, and fig. S13D), as previously observed (Fig. 1, A to C), suggesting that the lack of corticosterone affects this exchange.

Given the known increase in *Fkbp5* and corticosterone after 2 hours (Fig. 4C), we injected corticosterone (20 mg/kg) 60 min after the KXA injection in adrenalectomized females to stimulate a potential interaction via an additional corticosterone surge (*44*, *45*). Immediately after the corticosterone injection, the in vivo microglia-neuron interaction increased (Fig. 4, I to K; fig. S13D; and movies S3 and S4) with a slope comparable to non-adrenalectomized females (fig. S13E) and with a strong effect size (Hedges *g*: 1.74) based on the corticosterone half-life of 30 min (*46*). To determine whether this corticosterone exposure also promotes the interaction in males, we repeated this experiment in non-adrenalectomized males. Similar to adrenalectomized females, the males showed an increase in microglia-neuron interactions immediately after corticosterone administration (fig. S13, F and G) with a comparable slope (fig. S13E) and a strong effect size (Hedges *g*: 0.942). These data support a link between corticosterone-associated blood levels and microglia-neuron interaction via *Fkbp5*/Fkbp51.

Two recent studies demonstrated differences in microglial dynamics between awake and anesthetized mice using in vivo two-photon microscopy (*12*, *47*) and linked the microglial response to norepinephrine. To determine whether norepinephrine is also involved in our glucocorticoid female-selective response, we injected an adeno-associated virus encoding a norepinephrine sensor under the human synapsin promoter (*48*) into the V1Sp and, in parallel, performed a cranial window surgery (fig. S14A). After 2 weeks of recovery, we imaged noradrenergic release at baseline, after KXA anesthesia induction, and during the subsequent deep anesthesia and recovery phase. We found that fluorescence intensity increased significantly in females 60 min after KXA induction, lasted for ∼1 hour, and returned to baseline toward the end of the recordings (fig. S14, A to C), which is in line with studies in the amygdala (*49*). In contrast, males showed reduced fluorescence intensity immediately after KXA induction and did not show an increase in activity during the recovery. These data further emphasize sex-specific differences in mice recovering from KXA anesthesia, suggesting a well-regulated interplay among neuromodulator systems that affect neuronal plasticity, with microglia having a critical impact in this process (fig. S14D).

## DISCUSSION

Our study uncovers significant sex-specific differences in neuronal adaptation during recovery from ketamine anesthesia, driven by microglia. Specifically, female mice showed enhanced microglial activity in response to corticosterone elevation following ketamine anesthesia, facilitated through the *Fkbp5*/Fkbp51 pathway. This response increased synaptic density and neuronal plasticity, underscoring a critical role for microglia in female neural adaptation—a process not observed in males at the selected time point.

Corticosterone, the primary glucocorticoid in mice (*40*), is traditionally known to be released into the bloodstream during a physiological stress response or to regulate inflammation (*31*, *50*, *51*). Unexpectedly, we found that the recovery from ketamine anesthesia induced a similar response only in females (Fig. 4C) without additional stressors involved, such as psychological distress, physical restraint, or tissue trauma. As a lipophilic molecule, corticosterone enters cells by passive diffusion and binds to available intracellular glucocorticoid receptors, followed by the chaperone heat shock protein Hsp90 and one of the highly homologous immunophilin proteins Fkbp51 or Fkbp52 (*52*). Depending on the cell-intrinsic concentration of each Fkbp, either Fkbp51 sequesters the glucocorticoid receptor or Fkbp52 translocates the complex into the nucleus. There, the complex regulates transcription of diverse signaling pathways and *Fkbp5*, forming a negative feedback loop that regulates glucocorticoid receptor activity in the cell (*24*). Our data show that *Fkbp5* and Fkbp51 levels increased at 2 and 4 hours after KXA anesthesia induction, respectively (Fig. 2, D to G), specifically in microglia, resulting in increased reactivity reflected in a morphological shift and up-regulation of the endosomal-lysosomal marker CD68 (figs. S1 to S3). Specific Fkbp51 blockade or selective *Fkbp5* knockout in microglia prevented this effect (Fig. 3, A to F, and fig. S11). This positions the glucocorticoid pathway in microglia as a mediator of this sex-specific KXA recovery.

Previous gene expression datasets have shown that microglia highly express *Fkbp5* (*53*, *54*), which also emerged in our multiome screen, but follow-up studies have not further explored the consequences of *Fkbp5* in microglia for neuronal activity. The increased mEPSC frequency in pyramidal neurons of cortical layer II/III (Fig. 1H), which does not occur when *Fkbp5* is selectively knocked out in microglia (Fig. 3I), supports the functional integration of the newly formed synapses 4 hours after KXA. As in previous studies (*13*, *14*, *27*), increased microglia-dendrite contact appears critical during the recovery phase for mediating this effect, since microglia depletion prevents the increase in mEPSC frequency (Fig. 1H). One potential reason for the increased interaction might be to establish an environment that enables synapse formation by remodeling the extracellular space (*55*), similar to the response of microglia in cortical layer III-V, where CD68 colabeled with the PNN marker (figs. S3, C to F, S10, and S12, D and E). Yet the confirmation of this hypothesis would require additional experimental testing.

Our findings contribute to a growing body of evidence recognizing sex-specific differences in brain function and immune responses, the latter of which is already well known for increased susceptibility to infection and autoimmune diseases (*19*, *20*). Previous research has often overlooked sex distinctions, treating them as minor (*22*); however, recent advancements highlight the substantial impact of sex on physiological and behavioral responses (*21*). Both *Fkbp5* and microglia have been reported separately to show sex differences (*16*, *56*, *57*). Our results identified a link between microglia-specific *Fkbp5* expression and ketamine action (fig. S14D), warranting a reevaluation of assumptions that ketamine is a general anesthetic and fully reversible across sexes. Already at subanesthetic dosages, ketamine is metabolized differently (*6*, *58*). Also, more recent studies have raised the point that females may be affected differently during anesthesia with increased awareness during and faster emergence from general anesthesia (*59*) and that hormonal aspects are underappreciated (*60*). We previously showed that removing the main source of estrogen via ovariectomy shifts microglial morphology into an alternative state that does not resemble either male or female morphology (*7*). Yet, reducing differences between males and females just to estrogen levels might be too simplistic. Gonadal hormones such as estrogen, progesterone, and androgens can bias the stress response through direct and indirect effects on glucocorticoid receptors and downstream pathways (*23*, *61*) and on macrophage function (*62*). In line with this complexity, male animals did not exhibit a significant group-level increase in microglia-neuron interactions during KXA recovery, although a subset of males showed heterogeneous responses by imaging in vivo (fig. S6A), suggesting greater variability than in females.

Besides sex hormones, interactions between noradrenaline and corticosteroids have been described (*63*). Microglia selectively express the β2-adrenergic receptor (*64*, *65*) to detect norepinephrine, a neuromodulator of arousal (*66*). Adrenergic signaling has been shown to regulate microglial dynamics across different anesthetics, with decreased norepinephrine levels increasing microglial activity under isoflurane (*12*, *47*). Specifically, the study by Liu *et al.* (*12*) also included ketamine/xylazine and identified an increase in microglial process area and surveillance 20 to 30 min after anesthesia induction. Mechanistically, isoflurane binds to the GABA-A receptor, thereby preventing inhibition, while ketamine acts on NMDA receptors, leading to widespread excitation (*3*). When we analyzed microglia 4 hours after the start of isoflurane administration, they did not express CD68 (fig. S5). These findings could be related to differences in the actions of the anesthetics, or the experiments may require further optimization of the postanesthesia time point, since animals recover 10 times faster from isoflurane than from ketamine *(**3**,* *5**)*. Independently, we found a remarkably female-specific norepinephrine response during the recovery phase (fig. S14, A to C) that lasts 1 hour, consistent with the literature (*63*) and aligning with the prolonged contact between microglia and dendritic processes (Fig. 1B). On the basis of this result, we propose that during the KXA anesthesia recovery phase, noradrenaline may precede corticosterone release and ensure a constant corticosterone flow after 2 hours (Fig. 4C). Also, in a recent study in zebrafish, a subanesthetic dose of ketamine induces plasticity via norepinephrine, involving astrocytes (*67*). This finding brings another cell type into focus. Astrocytes also respond to norepinephrine (*68*) and express *Fkbp5* at baseline. *Fkbp5* has also been identified as a “marker” of neurotoxic A1 astrocytes (*69*, *70*), which we interestingly found to be down-regulated upon KXA in males (fig. S9, A and B). This emphasizes that future investigations into the effects of neuromodulators, such as corticosterone, are not limited to neurons and can have broad, sex-dependent systemic effects on signal processing across glial cells in the brain.

Last, a shortcoming of this study is that we used the *Cx3cr1* mouse model for our in vivo analysis, which robustly labels microglia in the brain parenchyma (Figs. 1, A to C, and 4, I and J). Yet, recent studies emphasize that *Cx3cr1* deficiency can affect microglia function (*71*–*73*). Although we used heterozygous animals and consistently measured them at the same time of day to avoid differences related to the light/dark cycle (*74*), we cannot rule out potential off-target effects, nonmicroglia expression, or spontaneous “leakiness” of the reporter leading to unintended baseline activity (*75*–*77*).

In conclusion, our study highlights significant sex-specific microglia-neuron interplay during recovery from anesthesia in the adult brain, mediated by the glucocorticoid pathway. Our findings show that circulating sex hormones are not the sole factor accounting for differences (*22*) and that microglia serve as a interface between the endocrine stress response and the brain-immune cell system.

## MATERIALS AND METHODS

### Animals

If not otherwise indicated, we used adult mice (8 to 12 weeks) of both sexes. C57BL/6J (catalog no. 000664), *Cx3Cr1*^CreERT2^ [B6.129P2(C)-*Cx3cr1*tm2.1(cre/ERT2)Jung/J, catalog no. 020940, always used heterozygous] (*78*), Ai9 [B6;129S6-Gt(ROSA)26Sortm9(CAG-tdTomato)Hze/J, catalog no. 007909] (*79*), Thy1-GFP [Tg(Thy1-EGFP)MJrs/J, catalog no. 007788] (*80*), and TRAP2 [(Fostm2.1(icre/ERT2)Luo/J), catalog no. 030323, crossed with Ai9 (*42*)] were purchased from the Jackson Laboratory. Fkbp5^flox/flox^ mice were provided by M. V. Schmidt (*39*). All mice were housed in the Institute of Science and Technology Austria (ISTA) Preclinical Facility with a 12-hour light-dark cycle. Food and water were provided ad libitum. The estrous phase was not systematically determined, except for the multiome analysis. All animal procedures are approved by the Bundesministerium für Wissenschaft, Forschung und Wirtschaft (bmwfw) Tierversuchsgesetz 2012 (TVG 2012), BGBI. I Nr. 114/2012, idF BGBI. I Nr. 31/2018 under the numbers 66.018/0005-WF/V/3b/2016, 66.018/0010-WF/V/3b/2017, 66.018/0025-WF/V/3b/2017, 66.018/0001_V/3b/2019, and 2020-0.272.234.

### Drugs

If not otherwise indicated, mice received intraperitoneal injections on weekdays in the morning. Since circadian cues might affect the experimental readout, we performed the experiments in the morning, when glucocorticoid levels are at their lowest (*81*, *82*).

#### Ketamine-xylazine-acepromazine

We combined ketamine (100 mg/kg; MSD Animal Health, catalog no. A137A01) with xylazine (10 mg/kg; Livisto, catalog no. 7630120), which prevents ketamine-induced muscle rigidity, and acepromazine (3 mg/kg; VANA GmbH, catalog no. 18F211), a phenothiazine tranquilizer (KXA) (*83*), solubilized in physiological saline solution [0.9% (w/v) NaCl, Fresenius Kabi Austria, catalog no. 19MIA700]. The solution was always freshly prepared to avoid pH fluctuations. As a control, we injected saline solution with the same volume as KXA. KXA was administered during the mice’s light phase, always between 9 and 10 a.m. (local time).

#### Tamoxifen

Adult *Cx3Cr1*^CreERT2/*het*^×Ai9 and *Cx3Cr1*^CreERT2/*het*^×Fkbp5^flox/flox^ mice were injected with tamoxifen (150 mg/kg; Sigma-Aldrich, catalog no. T5648, lot WXBD2299V) dissolved in corn oil (Sigma-Aldrich, catalog no. MKCH1635) for 3 consecutive days. Experiments started 5 days after the last injection.

#### 4-Hydroxy-tamoxifen

Injections were performed as previously described (*84*). Briefly, TRAP2-Ai9 mice were injected with 4-hydroxy-tamoxifen (50 mg/kg; Sigma-Aldrich, catalog no. H6278) in corn oil. The day before the experiment, 10 mg of 4-hydroxytamoxifen powder was solubilized in 1000 μl of pure ethanol (EtOH). The solution was divided into 150 μl of aliquots and stored overnight at −20°C. On the day of the experiment, the aliquots were suspended in 150 μl of corn oil, forming two phases: EtOH (top) and corn oil (bottom). The suspensions were centrifuged using a Speed Vac (Thermo Fisher Scientific, catalog no. SPD210) for 15 min at 40°C until the top phase was completely evaporated. The bottom phase, containing corn oil and 4-hydroxytamoxifen, was injected into the animals.

#### PLX5622

The animals received ad libitum chow 1.5 weeks before KXA injection, containing PLX5622 (1200 mg/kg; DC Chemicals, catalog no. DC21518), incorporated into the food pellets (Sniff, catalog no. S4865-E012). Mice were kept on this diet throughout the experiment (*85*, *86*). We opted for this strategy since current region-specific targeting requires invasive brain surgery that needs anesthesia and analgesia management, causes local inflammation, and is only short-lasting because microglia reoccupy within 3 to 7 days. Also, limiting to 1.5 weeks should avoid the reported side effects on neuronal activity (*87*–*89*).

#### Corticosterone

Corticosterone synthetic powder (≥92% purity, Sigma-Aldrich, catalog no. C2505) was solubilized in 50 μl of dimethyl sulfoxide (Sigma-Aldrich, catalog no. D8418) and subsequently diluted in a 1:5 ratio with saline to reduce toxicity (*90*). During the two-photon imaging session, adrenalectomized *Cx3Cr1*^CreERT2/het^×Ai9×Thy1-GFP mice were injected with corticosterone (20 mg/kg) (*91*).

#### SAFit2

SAFit2 (MedChemExpress, catalog no. HY-102080) was dissolved in 90% (v/v) corn oil and 10% (v/v) EtOH, and 20 mg/kg was injected (*92*).

#### Isoflurane

For fig. S5, C57BL/6J mice were anesthetized for 40 min with isoflurane (Zoetis, catalog no. 6089373, 5% induction, 2.5% maintenance) in O_2_ (0.6 liters/min) and were maintained at 37°C using a heating pad connected to a rectal probe during the entire procedure (Harvard Apparatus, catalog no. 14-516-264). The animals were then allowed to recover in their home cage and euthanized 4 hours after the initiation of the anesthesia with isoflurane. Control animals did not receive any treatments.

### Anesthesia induction

After the animals received the KXA injection, their eyes were treated with eye ointment (Oleo Vital) to prevent corneal dehydration, and they were maintained at 37°C throughout the procedure. The achievement of deep anesthesia was confirmed on the basis of the following parameters: (i) absence of the toe pinch reflex 10 min after induction; (ii) decrease in respiratory frequency; (iii) no responses to noxious stimuli; (iv) flaccid paralysis; and (v) absence of whisker movement (*93*).

### Immunostaining

#### Brain tissue preparation

If not otherwise indicated, the brain was dissected 4 hours after the drug treatment, exceeding the KXA half-life of 2 to 3 hours (*94*). The animal was quickly anesthetized with isoflurane (Zoetis, catalog no. 6089373) and secured to the perfusion plate. The chest was opened to expose the heart. The left ventricle was cannulated, and the inferior vena cava was cut. The animals were initially perfused with 20 ml of phosphate-buffered saline (PBS) containing heparin (100 mg/liter; Sigma-Aldrich, catalog no. H0878), followed by 20 ml of 4% (w/v) paraformaldehyde (PFA; Sigma-Aldrich, catalog no. P6148) in PBS using a peristaltic pump (Behr, catalog no. PLP 380; speed: 25 rpm). The animal was decapitated, and the brain was explanted and postfixed in 4% (w/v) PFA/PBS overnight (16 hours) at 4°C. Then, the tissue was washed in PBS and stored in PBS with 0.025% (w/v) sodium azide (VWR, catalog no. 786-299) at 4°C. For cryoprotection, the tissue was transferred to 30% (w/v) sucrose (Sigma-Aldrich, catalog no. 84097) in PBS and incubated overnight at 4°C. The brains were kept at −70°C in 30% (w/v) sucrose for long-term storage. If not otherwise indicated, the brain was sliced into 100-μm coronal sections on a vibratome (Leica VT 1200S).

#### Immunohistochemistry

The brain slices were incubated in a blocking solution containing 1% (w/v) bovine serum albumin (Sigma-Aldrich, catalog no. A9418), 5% (v/v) Triton X-100 (Sigma-Aldrich, catalog no. T8787), 0.5% (w/v) sodium azide (VWR, catalog no. 786-299), and 10% (v/v) serum (either goat, Millipore, catalog no. S26, or donkey, Millipore, catalog no. S30) for 1 hour at room temperature on a shaker. Afterward, the samples were immunostained with primary antibodies diluted in an antibody solution containing 1% (w/v) bovine serum albumin, 5% (v/v) Triton X-100, 0.5% (v/v) sodium azide, and 3% (v/v) goat or donkey serum for 48 hours on a shaker at room temperature. The following primary antibodies were used: rat α-CD68 (1:250; Bio-Rad, catalog no. MCA1957, lot 155083) for microglia reactivity (*95*); goat α-Iba1 (1:250; Abcam, catalog no. ab5076, lot 1014660-1); rabbit α-Iba1 (1:750; GeneTex, catalog no. GTX100042, lot 44200) for microglia (*96*); rabbit α-Fkbp5 (1:200; Thermo Fisher Scientific, catalog no. 711292, lot 2311462); guinea pig α-Vglut2 (1:1000; EMP Millipore, catalog no. AB2251-I, lot 3593077); and fluorescein-labeled *W. floribunda* lectin (1:200; Szabo-Scandic, catalog no.VECFL-1351, lot ZL0618). The slices were then washed three times with PBS and incubated, protected from light, for 2 hours at room temperature on a shaker, with the secondary antibodies diluted in an antibody solution. The secondary antibodies raised in goat or donkey were purchased from Thermo Fisher Scientific (1:2000; Alexa Fluor 488, Alexa Fluor 568, and Alexa Fluor 647). The slices were washed three times with PBS. The nuclei were labeled with Hoechst 33342 (1:5000; Thermo Fisher Scientific, catalog no. H3570) diluted in PBS for 15 min. The slices were mounted on microscope glass slides (Assistant, catalog no. 42406020) with coverslips (Menzel-Glaser #1, Fisher Scientific, catalog no. 17234914) using an antifade solution consisting of 10% (v/v) mowiol (Sigma-Aldrich, catalog no. 81381), 26% (v/v) glycerol (Sigma-Aldrich, catalog no. G7757), 0.2 M tris buffer (pH 8), and 2.5% (w/v) 1,4-Diazabicyclo[2.2.2]octane (DABCO, Sigma-Aldrich, catalog no. D27802).

#### Microscopy

*Confocal microscopy*. Images were acquired using Zeiss LSM800 or LSM900 upright microscopes with a Plan-Apochromat 40×/1.3 Oil differential interference contrast (DIC), ultraviolet-infrared (catalog no. 420762-9800-799) or a Plan-Apochromat 40×/1.3 Oil DIC M27 (catalog no. 420762-9800-799), respectively. Z-stack images were obtained as a 2 × 2 tile scan, or 4 × 2 for whole VISp layers, at a resolution of 0.156 μm × 0.156 μm × 0.24 μm. Images from TRAP2 mice were captured with a Zeiss LSM800 using a Plan-Apochromat 20×/0.8 (catalog no. 420650-9901).

*Super-resolution microscopy*. The z-stack images of the dendritic spines in cortical layer II/III in Thy1-EGFP were acquired using a Zeiss LSM900 upright microscope equipped with a second-generation Airyscan module optimized for a Plan-Apochromat 40×/1.3 Oil DIC M27 (catalog no. 420762-9800-799). Images were acquired at a resolution of 0.045 μm × 0.045 μm × 0.19 μm.

#### Image processing and analysis

Confocal tile images were converted to .ims using Imaris File Converter 9.9.1v and stitched with Imaris Stitcher 9.9.1.v. The images were processed using the Imaris 9.9.1.v processing tool, applying a Gaussian filter width of 0.156 μm, setting the background subtraction to 76.2 μm, and activating the layers’ normalization.

*Reconstruction of 3D segmented microglia*. Microglial cells were traced in 3D with the filament-tracing plugin on Imaris 9.9.1v. Filament starting points were set on microglial somata with a diameter of 7.8 μm and seeding points of 0.5 μm. Disconnected segments were removed using a filtering smoothness of 0.5 μm. After tracing, microglial cells at the image border that were only partially traced were manually removed. The data analysis was performed blinded. The Vglut2 staining, concentrated in layers I and IV (*97*), distinguished cortical layers in the VISp. Microglia with somas located in layer I or IV were labeled as layer I or IV microglia, respectively, with layer II/III microglia in between and layer V/VI microglia below. The generated layer-discriminated skeleton images were converted from .ims format (Imaris) to .swc format (*98*) by first obtaining the 3D positions and the diameter of each traced microglial process using the ImarisReader toolbox for MATLAB (https://github.com/PeterBeemiller/ImarisReader/) and then exporting to the standard format using the Neuroland Morphology Converter (http://neuroland.org/). Artifacts from the 3D reconstructions were automatically unable to be converted to the .swc format.

*Microglia morphological analysis with morphOMICs*. 3D segmented microglia were analyzed using morphOMICs, a topological data analysis framework designed to extract and interpret microglial morphological phenotypes (*7*). The morphOMICs pipeline comprises several sequential steps: First, the topological morphology descriptor (*99*) was applied using the radial distance from the soma as a filter function to capture the topological features of each segmented microglial structure via a persistence barcode. Then, persistence barcodes were filtered out on the basis of the following criteria: (i) fewer than five or more than 250 bars; (ii) the longest bar exceeded 110 microns; (iii) more than 10 bars with a death distance of 0 microns. After visual inspection, we confirmed that these barcodes resulted from tracing errors, overlapping cells, wrongly segmenting two cells as one, or failures in the .swc file format conversion. From our dataset of 3730 persistence barcodes, we excluded 7 persistence barcodes. Next, each barcode was vectorized using the persistence image transformation (*100*). Then, we applied multiple dimensionality reduction techniques to the persistence images to visualize microglia’s morphological embeddings and to explore condition-dependent morphological variation using a variational autoencoder (VAE) (*101*). Before applying VAE, preliminary dimensionality reductions were performed. First, pixels with an SD below 1 × 10^−5^ across all persistence images in the microglia dataset were removed. This step aimed to eliminate pixels consistently black across the dataset, typically located far from regions of high persistent feature density in the images. Subsequently, the dataset underwent *z*-score standardization to ensure that each feature had a mean of zero and an SD of one. Last, principal components analysis (PCA) (*102*) was conducted using the scikit-learn implementation with default parameters.

VAE was developed using PyTorch (v2.6.0; CUDA 12.6). The VAE encoder was used to capture the underlying data structure, and the decoder was used to reconstruct embedded persistence images for interpretation of the embedding (latent space). Both were implemented as multilayer perceptrons. The encoder comprised three hidden layers with sizes [32, 16, 8], while the decoder mirrored the encoder architecture with layers of sizes [8, 16, 32]. Scaled Exponential Linear Unit activation functions were used for all hidden layers (*103*). The latent space was set to dimension 2 to facilitate visualization and interpretation. The VAE was trained on the first 64 principal components derived from the PCA applied to persistence images, which collectively accounted for more than 99% of the total variance and facilitated efficient training. Weights in the network were initialized using the Kaiming uniform initialization method. The COntinuous COin Betting optimizer (*104*) was used to eliminate the need for manual learning rate tuning. Training was conducted over 2000 epochs with a batch size of 8. A Kullback-Leibler (KL) annealing strategy was implemented to balance the reconstruction loss and the KL divergence. Specifically, the weight of the KL term was gradually increased from approximately 0 to 1 following an exponential schedule, defined by the function w(x)=1−(e−x), where x values were linearly spaced between 2 and 7 (i.e., x=5∗i/1999+2, with i the epoch index between 0 and 1999). This approach enabled the model to focus on accurate reconstruction during the initial training phase, progressively encouraging the latent space to conform to a standard normal distribution.

An interpolation composed of five points was constructed by performing a linear regression in the VAE’s latent space on the medians of the female and male groups for both KXA and saline to compute the slope and intercept. The 2D embedded persistence images in the VAE latent space were then projected onto this interpolation line, yielding a 1D representation of the distribution across the four groups. After assessing normality using the Shapiro-Wilk test, we performed a Kruskal-Wallis test, followed by a post hoc Dunn’s test with Bonferroni correction for multiple comparisons. Statistical significance was determined at a *P* value threshold of 0.05. A Jupyter notebook reproducing the results in this paper and the corresponding parameter file can be found at https://github.com/siegert-lab/V1_morphOMICs.

*CD68 volume within microglia*. Surface renderings were generated on microglia and CD68 z-stacks using the surface-rendering module of Imaris 9.9.1.v. Surfaces were created with a surface detail of 0.2 μm. The surface-surface colocalization plugin was used to identify the CD68 surface within microglia. This analysis was performed on the entire image. The total ratio of CD68 volume to microglial volume (CD68-to-microglial volume) was calculated as (CD68 volume/total microglia volume) × 100 for each image.

*PNN inside the CD68 volume within microglia*. Surface renderings of microglia, CD68, and PNN were generated from z-stacks using the surface-rendering module of Imaris 9.9.1.v. Surfaces were created with a surface detail setting of 0.2 μm. The CD68 surface within microglia was first identified using the surface-surface colocalization plugin, which generated a new surface module. Subsequently, the surface-surface colocalization plugin was applied to identify overlapping surfaces between the microglia-CD68 colocalization and the PNN. The PNN ratio within the microglial CD68 volume was calculated for each image.

*Dendritic spine quantification*. The density of dendritic spines from super-resolution images was quantified semiautomatically using Imaris 9.9.1v. The manual drawing module was activated through the filament tracing function. A starting point was established at the beginning of the dendrite, and the dendrite was traced with the autopath function. The spine density was automatically calculated using the Imaris filament creation module by entering the dendrite diameter, the thinnest point (corresponding to the spine neck), and the maximum length measured for each dendrite.

### Slice electrophysiology recording

Acute sagittal brain slices containing the primary visual cortex (VISp) were derived from P60-P90 C57BL/6J male and female mice. Four hours after the injection of KXA or saline, the mice were anesthetized with isoflurane and briefly transcardially perfused with ice-cold, oxygenated (95% O_2_, 5% CO_2_) artificial cerebrospinal fluid (ACSF) containing 118 mM NaCl, 2.5 mM KCl, 1.25 mM NaH_2_PO_4_, 2 mM MgCl_2_, 1 mM CaCl_2_, 26 mM NaHCO_3_, 3 mM myo-inositol, 20 mM sucrose, and 10 mM glucose (pH = 7.35 to 7.40). The brain was rapidly removed and mounted, and 300-μm-thick coronal slices were cut on a vibratome (VT1200S, Leica). The acute brain slices were incubated at 32°C in ACSF for 20 min and then slowly cooled to room temperature over an additional 40 min. For recordings, brain slices were transferred to a recording chamber on an LNScope 240 XY (Luigs & Neumann) and superfused with ACSF (22° to 24°C) at a rate of 3 ml min^−1^ with a peristaltic pump (MP3, Gilson). Layer II/III pyramidal neurons were recorded through patch pipettes (3 to 5 megohm) made from borosilicate glass capillaries pulled on a P1000 glass puller (Sutter Instrument). Patch pipettes were filled with an intracellular solution containing (pH 7.3, adjusted with KOH; 302 milliosmoles): 120 mM K-gluconate, 20 mM KCl, 0.5 mM EGTA, 2 mM MgCl_2_, 10 mM Hepes, 2 mM sodium adenosine triphosphate (Na-ATP), 0.2 mM sodium guanosine triphosphat (Na-GTP), 23 mM sucrose, and 0.5% biocytin (w/v) (Tocris, 3349). mEPSCs were recorded in voltage clamp at −60 mV, in the presence of 1 μM TTX (Tocris, 1069), 10 μM bicuculline (Tocris, 0109), and 2 mM CaCl_2_, using a Multiclamp 700B amplifier (Molecular Devices) connected to a 1550B digitizer (Molecular Devices), sampled at 10 KHz, and filtered at 2 KHz. The frequency and amplitude of the mEPSCs were analyzed using the software Simplyfire (*105*).

### In vivo live imaging

#### Cranial window surgery

*Cx3Cr1*Cre^ERT2/het^×Ai9×Thy1-GFP mice, 8 to 10 weeks old, were anesthetized with isoflurane (Zoetis, catalog no. 6089373; 5% induction, 2.5% maintenance) in O_2_ (0.6 liters/min) and were maintained at 37°C using a heating pad connected to a rectal probe during the surgical procedure (Harvard Apparatus, catalog no. 14-516-264). The mice were positioned in a stereotaxic frame (KOPF digital plus). Eye ointment (Oleovital) was applied to prevent corneal dehydration. The head was shaved and disinfected with 70% (v/v) EtOH in H_2_O; an incision in the scalp was made, and the skin flap covering both hemispheres was removed. The periosteum was treated with a 3% (v/v) hydrogen peroxide (Sigma-Aldrich, catalog no. 216763) in PBS solution. A 3-mm biopsy tool (Henry Schein, catalog no. 394-314) was used to mark the skull above VISp for drilling, and a craniotomy centered 3.6 mm posterior to bregma and 1.65 mm interaural was performed on the right hemisphere with a 0.9-mm micro drill (Fine Science Tools, catalog no. 19007-09). After achieving hemostasis, the exposed brain was covered with a double glass window made of 3- and 5-mm glass coverslips (Multi Channels System, catalog nos. 640720 and 640731) glued together with glass superglue (UHU Glass Special Glue). The glass window was then fixed to the skull using dental cement (Kulzer Paladur, catalog nos. 64707948 and 64707938). A custom-made metal frame was secured to the skull with dental cement. Pain control was administered with metamizol (Sanofi Aventis, catalog no. Ay005; sc 200 mg/kg during surgery) and meloxicam (Boehringer-Ingelheim, catalog no. KPOEH3R, sc 5 mg/kg after surgery every 24 hours for 3 consecutive days). To minimize disruption of neuroimmune function during surgery, we ensured that the pia mater and brain parenchyma were not damaged. Afterward, the animal recovered for at least 2 weeks to resolve acute inflammation and astrogliosis (*106*–*108*). We previously confirmed that this time period was sufficient. We only used animals if they showed no signs of discomfort after 2 weeks and had a clear cranial window.

#### Two-photon microscopy

Awake *Cx3Cr1*^CreERT2/het^×Ai9×Thy1-GFP mice were head-fixed on a custom-made microscopy stage and imaged with a Leica TCS SP8 DIVE CS microscope equipped with an HC FUOTAR L 25×/0.95 W (catalog no. 15506374), working distance  = 2.5 mm, wide angle (41°). Two lasers were used in this experiment: a tunable laser at 920 nm and a nontunable one at 1045 nm. Approximately 100-μm z-stack images at a resolution of 1600 × 1600 pixels and a *z*-step of 2 μm were acquired starting 50 μm below the dura. The attenuation correction was activated on the laser power to compensate for signal reduction during the z-stack acquisition. Images were acquired bidirectionally with an *x*-phase of 31.55 using the two HyD SP GaAsPDetectors, one for GFP and one for Ai9, with a set gain of 100. The time interval between the z-stacks was 5 min. The experiment began with 30 min of awake recording. Imaging was paused, and the animal received an intraperitoneal injection of KXA or KXA plus corticosterone (20 mg/kg). Imaging was then resumed until a total duration of 210 min.

#### Image processing

Two-photon time-lapse images were converted to .ims using Imaris File Converter 9.9.1v. A spot with a diameter of 8 μm was created on the soma of each microglia cell using the Imaris spot function. A translational drift correction of image and spot objects was performed. Images were processed with the Imaris 9.9.1.v processing tool, using a Gaussian filter width of 0.174 μm, a background subtraction set to 11.2 μm, and activated normalization of the layers.

#### Virus injection

C57BL/6J mice, 8 to 10 weeks old, were anesthetized with isoflurane (Zoetis, catalog no. 6089373; 5% induction, 2.5% maintenance) in O_2_ (0.6 liters/min) and were maintained at 37°C using a heating pad connected to a rectal probe during the surgical procedure (Harvard Apparatus, catalog no. 14-516-264). The mice were positioned in a stereotaxic frame (KOPF digital plus). Eye ointment (Oleovital) was applied to prevent corneal dehydration. The head was shaved and disinfected with 70% (v/v) EtOH in H_2_O; an incision in the scalp was made, and the skin flap covering both hemispheres was removed. The periosteum was treated with 3% (v/v) hydrogen peroxide (Sigma-Aldrich, catalog no. 216763) in PBS solution. A 3-mm biopsy tool (Henry Schein, catalog no. 394-314) was used to mark the skull above VISp for drilling, and a craniotomy centered 3.6 mm posterior to bregma and 1.65 mm interaural was performed on the right hemisphere with a 0.9-mm micro drill (Fine Science Tools, catalog no. 19007-09). At the center of the craniotomy, 0.8 ml of AAV9_hSyn1_nLightG virus (Addgene, catalog no. 187179-AAV9) at a titer of ≥1 × 10^13^ vg/ml was injected with a pulled glass capillary [World Precision Instruments (WPI), catalog no. 504949]. The injection was performed using a Nanoliter2020 Injector equipped with a MICRO2T SMARTouch controller (WPI, catalog no. 300704) at a rate of 1 nl/s. The injection started 800 μm below the dura mater, and the glass capillary was lifted 100 μm every minute, reaching a final injection depth of 200 μm. After the injection, the exposed brain was covered with a double glass window made of 3- and 5-mm glass coverslips (Multi Channels System, catalog nos. 640720 and 640731) glued together with glass superglue (UHU Glass Special Glue). The glass window was then fixed to the skull using dental cement (Kulzer Paladur, catalog nos. 64707948 and 64707938). A custom-made metal frame was secured to the skull with dental cement. Pain control was administered with metamizol (Sanofi Aventis, catalog no. Ay005, sc 200 mg/kg during surgery) and meloxicam (Boehringer-Ingelheim, catalog no. KPOEH3R, sc 5 mg/kg after surgery every 24 hours for 3 consecutive days).

#### Norepinephrine imaging in vivo

Awake C57BL/6J mice were head-fixed on a custom-made microscopy stage and imaged with a Leica TCS SP8 DIVE CS microscope equipped with an HC FUOTAR L 25×/0.95 W (catalog no. 15506374), WD = 2.5 mm, wide angle (41°). The imaging was performed with a tunable laser set to 920 nm. Approximately 25-μm z-stack images at a resolution of 512 × 512 pixels, a frequency of 400 Hz, and a *z*-step of 2 μm were acquired. Images were acquired using the HyD SP GaAsPDetectors, one for eGFP, with a set gain of 100. Time-lapse images were acquired for 5 min. The time interval between each acquisition was 25 min. The experiment began with a baseline awake recording. Imaging was paused, and the animal received an intraperitoneal injection of KXA. Imaging was then resumed until a total duration of 210 min.

#### Norepinephrine fluorescence analysis

The norepinephrine fluorescence intensity was quantified in Fiji (version 2.16) by manually defining one region of interest (ROI) over the background and two ROIs over regions with viral expression. The fluorescence from the two ROIs was averaged over each frame. Background-subtracted mean fluorescence was extracted for each frame and expressed as Δ*F*/*F*_0_, calculated using the equation Δ*F*/*F*_0_ = [*F*(*t*) − *F*0]/*F*0 where *F*_0_ was defined as the mean fluorescence during the baseline period and *F*(*t*) as the averaged fluorescence of the two ROIs at time (*t*).

### Single-nucleus multiome sequencing

#### Tissue preparation

C57BL/6J females, 8 weeks old, littermates in the follicular phase, received KXA or saline intraperitoneal injection. Two hours after the treatment, the animals were briefly anesthetized with isoflurane and perfused with ice-cold Hepes cutting solution containing 110 mM NaCl, 10 mM Hepes, 25 mM glucose, 75 mM sucrose, 6 mM MgCl_2_, 7.5 mM MgCl_2_, and 2.5 mM KCl. The pH of the solution was adjusted to 7.4 using 1 N NaOH. After being perfused with 20 ml of ice-cold Hepes cutting solution, the brain was explanted and immediately frozen in liquid N_2_ vapors for 2 min. The brains were stored at −70°C in 5-ml Eppendorf tubes containing optimal cutting temperature (OCT) compound (A. Hartenstein, catalog no. TTEK). The samples were shipped to the Allen Institute on dry ice .

#### Sequencing

*cDNA amplification and library construction*. For single-nucleus multiome library generation, we used the Chromium Next GEM Single Cell multiome ATAC + Gene Expression Reagent Bundle (10× Genomics, catalog no. 1000283). We adhered to the manufacturer’s instructions for transposition, nucleus capture, barcoding, reverse transcription, cDNA amplification, and library construction [Allen Institute for Brain Science. 10x multiome sample processing. Protocols.io https://doi.org/10.17504/protocols.io.bp2l61mqrvqe/v1 (2023)].

For the sn-multiome libraries, we loaded 11,154 ± 1386 nuclei per port. For single-nucleus RNA sequencing, we targeted a sequencing depth of 120,000 reads per nucleus. The actual average achieved for the nuclei included in this study was 87,183 ± 24,943 reads per nucleus. For single-nucleus Assay for Transposase-Accessible Chromatin using sequencing (snATAC-seq), we targeted a sequencing depth of 85,000 reads per nucleus. The actual average achieved for the nuclei included in this study was 90,470 ± 7780 reads per nucleus across four libraries. The snM-multiome libraries were sequenced on the Illumina NovaSeq 6000, and sequencing reads were aligned to the mouse references downloaded from 10× Genomics, including the Ensembl GRCm38 (v98) fasta file and the gencode (vM23) gtf file, using the 10× Genomics CellRanger Arc (v2.0) workflow with default parameters.

#### Data analysis

*Quality control*. We loaded the unique molecular identifier (UMI) count matrices for each mouse sample in R (v4.3.1, Linux distribution) as a Seurat object (*109*), requiring that each gene be expressed in at least five captured nuclei and that each cell contain at least 300 genes with nonzero counts. For each sample, we implemented scDblFinder (*110*) to detect potential doublets—two nuclei that were sequenced as one—with the following parameters: “min.mean”: 0.1, “includePCs”: 20, “nfeatures”: 1250, and “seed”: 34151 for reproducibility. After filtering out the inferred doublets, we further filtered out nuclei with total counts and unique genes greater than 3 MAD (median absolute deviation) across all nuclei sequenced per sample (fig. S5, A and B). To focus more on canonical genes and further reduce noise, we drop pseudogenes and noncoding RNAs (i.e., genes whose names start with “Gm” or end with “Rik”), as well as microRNAs (genes starting with “Mir” and ending with “hg”).

*Normalization and integration*. We log-normalized the filtered count matrix using Seurat’s NormalizeData(). We identified 3000 highly variable genes using the vst selection method with FindVariableFeatures(), which we used as anchors to integrate the samples with SelectIntegrationFeatures(), FindIntegrationAnchors(), and lastly IntegrateData(). We scaled the integrated dataset using ScaleData() and conducted an initial PCA using RunPCA(), retaining only the first 50 PCs. To visualize potential batch effects, we inferred a 2D manifold using RunUMAP() with the following parameters: “umap.method”: “umap-learn,” “metric”: “cosine,” “n.neighbors”: 100, “n.components”: 2, “spread”: 1, “densmap”: 0, and “seed.use”: 5143 for reproducibility (fig. S5, C and D).

*Cell type assignment*. To extract high-resolution clusters of nuclei with similar transcriptomic profiles, we implemented the Louvain algorithm using FindNeighbors() with the following parameters: “k.param”: 20, “nn.method”: “annoy,” “annoy.metric”: cosine, “l2.norm”: 0 and then with FindClusters() with the following parameters: “resolution”: 0.5, “algorithm”: 1 for the original Louvain algorithm, and “random.seed”: 3617 for reproducibility. We then used SingleR (*111*) to annotate cluster identities with the Allen Brain Atlas VISp transcriptomic cell type annotations (*112*) as a reference. Note that this Allen Brain annotation contains cellular subtypes based on specific gene markers. Here, we focus on the main cell type annotations. We confirm the cluster identities using known gene markers (fig. S5, E to H).

*Interaction model*. We estimated the interaction effect of ketamine on neuronal versus astrocytes/microglia cell types while accounting for intermouse variability across the four female mice within the two groups (saline and KXA) using an LMM. In this way, the model favors gene expression changes that are consistent across mice. Furthermore, we reduced variability in cell type proportions by broadly defining cell types as neuronal or nonneuronal (i.e., astro for astrocytes and micro for microglia). This broad grouping resulted in similar variability across neuronal cells: 0.885, 0.854, 0.876, and 0.869 for ctrl1, ctrl2, kxa1, and kxa2, respectively, allowing us to find significant differences between control and KXA-treated females. For fixed effects in the LMM, we estimated the ketamine effect, the cell type effect, and the interaction between ketamine and cell type. We accounted for variability across mice as random effects. We fit the model for each of the top 2000 highly variable genes, calculated using binomial deviance (*113*).

For each gene *g*, we fit an LMM

$$Y_{g}=X\beta_{g}+Zu_{g}+\epsilon_{g}$$

where $Y_{g}$ is the log-normalized mRNA abundance for gene *g*. We calculated $Y_{g}$ by log transforming and normalizing the raw counts x: $Y_{g}=\text{log}\;\left(\frac{x_{g}}{\sum_{\mathrm{i\epsilon G}}x_{i}}\;10^{5}+1\right)$. *G* is the set of highly variable genes.

X is the design matrix for fixed effects. For the full model, it is *N* by 4, and for the null model, it is *N* by 3 (*N* = 37,054 cells). The four columns denote (i) intercept, (ii) whether the cell is an astrocyte/microglia cell type, (iii) whether the cell is treated with ketamine, or (iv) whether the cell is both an astrocyte/microglia cell type and treated with ketamine. In this design matrix, for example, a neuronal cell type without ketamine treatment (intercept case) would be (1, 0, 0, 0). In the null model, we dropped the fourth column to assume no interaction.

β is a vector of fixed effects, corresponding to log fold changes across different conditions. For the full model, the four fixed effects are (i) intercept (log mRNA abundance of a neuronal cell type with no ketamine treatment), (ii) log fold change for being an astrocyte/microglia cell type, (iii) log fold change of ketamine treatment, or (iv) interaction effect of ketamine treatment and astrocyte/microglia cell type.

Z is the design matrix for random effects of size *N* by *m*, where *m* is the number of mice in the experiment (*m* = 4 mice). u is a vector of random effects.

We fit the data with the full model and compared it with the null model to identify genes for which the interaction between ketamine treatment and astrocyte/microglial cell type was significant. We fit the full model using the lme4 R package with the R formula: Y∼cell type+ketamine+cell type:ketamine+(1∣mouse). For the null model, we set the interaction effect to zero: Y∼cell type+ketamine+(1∣mouse). We compared the two model fits using analysis of variance (ANOVA) implemented in lme4 to obtain a *P* value: anova(null model,full model), which uses an *F*-statistic calculation (*114*).

#### snATAC-seq analysis

We also analyzed the snATAC-seq data in our multiome experiment. Many gene expression changes were larger than the chromatin accessibility changes, suggesting a limited sensitivity of the ATAC-seq signal relative to RNA sequencing and therefore cannot provide additional insights.

### Fluorescence in situ hybridization

C57BL/6J mice of both sexes, aged 8 to 10 weeks, were perfused with 4% (w/v) PFA in PBS 2 hours after the injection of KXA or saline, in line with the timeline of the single-nucleus multiome sequencing experiment. The explanted brains were postfixed in 4% (w/v) PFA in PBS for 24 hours at 4°C on an orbital shaker. Tissues were cryoprotected in an ascending sucrose gradient (10% (w/v), 20% (w/v), and 30% (w/v) in PBS) until the tissues sank to the bottom of the container (∼18 hours per step). The brains were embedded in OCT (A. Hartenstein, catalog no. TTEK), and 10-μm slices were cut using a cryostat (Thermo Fisher Scientific, NX70, catalog no. 957000L). The slices were stored at −70°C. The fluorescence in situ hybridization experiment was conducted using the RNAScope Intro Pack for Multiplex Fluorescence Reagent Kit v2 (Bio-Techne, catalog no. 323136) according to the manufacturer’s protocol. In this experiment, we identified the following probes: Fkbp5 [probe: Mm-Fkbp5–*Mus musculus* FK506 binding protein 5 (*Fkbp5*) mRNA, catalog no. 457241], Cx3cr1 [*M. musculus* chemokine (C-X3-C) receptor 1 (*Cx3cr1*) mRNA, catalog no. 314221-C2], NeuN [probe: Mm-Rbfox3-C2–*M. musculus* RNA binding protein fox-1 homolog (*Caenorhabditis elegans*) 3 (*Rbfox3*/*NeuN*) transcript variant 1 mRNA, catalog no. 313311-C2], and S100β [probe: Mm-S100b-C3–*M. musculus* S100 protein beta polypeptide neural (*S100b*) mRNA, catalog no. 431731-C3]. The probes were visualized using TSA Vivid Dyes linked to 520-, 570-, or 650-nm fluorophores (Bio-Techne, catalog nos. 323271, 323272, and 323273, respectively). The nuclei were labeled using 4′,6-diamidino-2-phenylindole (available in the kit). The z-stack images were acquired in super-resolution using a Zeiss LSM 900 upright microscope, equipped with an Airyscan second-generation module optimized for a Plan-Apochromat 40×/1.3 Oil DIC M27 (catalog no. 420762-9800-799). Images were captured at a resolution of 0.045 μm × 0.045 μm × 0.19 μm. The images were analyzed in Imaris 9.9.1v using the Spot function. The results were expressed as puncta density (*n* puncta/area).

### Blood corticosterone measurement

C57BL/6J mice of both sexes, 8 to 10 weeks old, were injected with saline or KXA. Animals were collected either 30 min or 2 hours after the injection, by briefly placing them in an induction chamber with 4 to 5% isoflurane. Once the loss of reflexes was confirmed, the skin was sterilized with 70% (v/v) EtOH, and the chest and abdomen were opened. The inferior vena cava was cut, and the systemic blood was collected in a 2-ml Eppendorf tube. The blood was allowed to coagulate for 15 min at room temperature. The samples were centrifuged (Eppendorf 5224R cooling, catalog no. 5424000410) at 4°C for 10 min at 2000*g* to separate the serum. The supernatant was collected and stored at −70°C. The corticosterone was quantified using an enzyme-linked immunosorbent assay (ELISA) competitive kit (Biocat, catalog no. K014-C1-AAS) following the manufacturer’s instructions. The results were read using an ELISA plate reader (BioTek Synergy H1).

### Adrenalectomy surgery

C57BL/6J females, 8 to 10 weeks old, were anesthetized with isoflurane (5% induction, 2.5% maintenance) at a rate of 0.6 liters/min O_2_ and were maintained at 37°C using a heating pad connected to a rectal probe during the surgical procedure. After shaving and sterilizing the skin, adrenalectomy was performed using a dorsal midline incision ∼1 cm long, located at the level of the third lumbar vertebrae, caudal to the last rib, to access the abdominal cavity. The kidney, adrenal gland, and associated fat pad were identified, and the vessels supplying the gland were cauterized. The gland was then excised at its base. The procedure was performed bilaterally. The abdominal cavity and skin were sutured using Dafilon Blue 4/0 1.5 surgical sutures (Henry Schein, catalog no. 656-476). To prevent changes in circadian rhythm, adrenalectomized mice were given corticosterone (25 μg/ml) in drinking water, approximating basal corticosterone levels and mimicking the typical circadian secretion pattern (*43*). The mice were allowed to recover for at least 2 weeks. Pain was managed with metamizol (Sanofi Aventis, catalog no. Ay005, sc 200 mg/kg during surgery) and with meloxicam (Boehringer-Ingelheim, catalog no. KPOEH3R, sc 5 mg/kg after surgery every 24 hours for 3 consecutive days.

### Statistical analysis

Data were analyzed using GraphPad Prism 10.2.2. The datasets were tested for normal distribution using the Shapiro-Wilk test. For multiple comparisons analyses of normally distributed data, we used a two-way ANOVA. If a significant effect (*P* < 0.05) was observed, we applied Tukey’s correction for multiple comparisons. We used a Friedman test for multiple comparison analyses of nonnormally distributed data. Two sets of unpaired variables that showed normal distribution were compared using the Student’s *t* test, whereas, in the case of nonnormal distribution, the Mann-Whitney test was used. Data in the plots are expressed as means ± SEM. Statistical significance across groups was determined using the nonparametric Kruskal-Wallis test. If a significant effect (*P* < 0.05) was found, post hoc pairwise comparisons were conducted using the Dunn’s test to account for multiple comparisons. Group differences with unequal variances across groups were analyzed using the Welch’s ANOVA test. When a significant effect was detected, the Dunnett’s T3 multiple comparisons test was used to compare each treatment group with the control group. Repeated technical measurements obtained from the same animal were analyzed using a nested design to account for within-animal dependence. For comparisons involving multiple groups, a nested one-way ANOVA was performed. Statistical significance was indicated as *P < 0.05, **P < 0.01, ***P < 0.001, or *P* > 0.05 (not significant). Effect sizes were quantified using Hedges’ *g*, calculated in Python. An effect size was interpreted as small when *g* < 0.2, medium when *g* < 0.5, and large when *g* > 0.8. If not otherwise indicated, graphs show mean and ± SEM. A detailed statistical analysis is available in table S1.

### Image graphics

The image graphics of Fig. 2A was created by BioRender.com. All other image graphics from Figs. 3 (A and D) and 4 (A and D) and figs. S10A, S12A, and S14 (A and D) were created by the authors with Adobe Illustrator 2026, v30.4

## References

[1] J. W. Olney, J. W. Newcomer, N. B. Farber, NMDA receptor hypofunction model of schizophrenia. J. Psychiatr. Res. 33, 523–533 (1999). 10628529 10.1016/s0022-3956(99)00029-1

[2] J. Seamans, Losing inhibition with ketamine. Nat. Chem. Biol. 4, 91–93 (2008). 18202677 10.1038/nchembio0208-91

[3] E. N. Brown, P. L. Purdon, C. J. Van Dort, General anesthesia and altered states of arousal: A systems neuroscience analysis. Annu. Rev. Neurosci. 34, 601–628 (2011). 21513454 10.1146/annurev-neuro-060909-153200 PMC3390788

[4] A. Venturino, R. Schulz, H. D. Jesús-Cortés, M. E. Maes, B. Nagy, F. Reilly-Andújar, G. Colombo, R. J. A. Cubero, F. E. S. Uiterkamp, M. F. Bear, S. Siegert, Microglia enable mature perineuronal nets disassembly upon anesthetic ketamine exposure or 60-Hz light entrainment in the healthy brain. Cell Rep. 36, 109313 (2021). 34233180 10.1016/j.celrep.2021.109313 PMC8284881

[5] K. Hohlbaum, B. Bert, S. Dietze, R. Palme, H. Fink, C. Thöne-Reineke, Impact of repeated anesthesia with ketamine and xylazine on the well-being of C57BL/6JRj mice. PLOS ONE 13, e0203559 (2018). 30231081 10.1371/journal.pone.0203559 PMC6145541

[6] J. N. Highland, C. A. Farmer, P. Zanos, J. Lovett, C. A. ZarateJr, R. Moaddel, T. D. Gould, Sex-dependent metabolism of ketamine and (2R,6R)-hydroxynorketamine in mice and humans. *J. Psychopharmacol.* (*Oxf.*) 36, 170–182 (2022). 10.1177/02698811211064922 PMC9904319 34971525

[7] G. Colombo, R. J. A. Cubero, L. Kanari, A. Venturino, R. Schulz, M. Scolamiero, J. Agerberg, H. Mathys, L.-H. Tsai, W. Chachólski, K. Hess, S. Siegert, A tool for mapping microglial morphology, morphOMICs, reveals brain-region and sex-dependent phenotypes. Nat. Neurosci. 25, 1379–1393 (2022). 36180790 10.1038/s41593-022-01167-6 PMC9534764

[8] I. Hristovska, M. Robert, K. Combet, J. Honnorat, J.-C. Comte, O. Pascual, Sleep decreases neuronal activity control of microglial dynamics in mice. Nat. Commun. 13, 6273 (2022). 36271013 10.1038/s41467-022-34035-9 PMC9586953

[9] M. Valenza, R. Facchinetti, C. Torazza, C. Ciarla, M. R. Bronzuoli, M. Balbi, G. Bonanno, M. Popoli, L. Steardo, M. Milanese, L. Musazzi, T. Bonifacino, C. Scuderi, Molecular signatures of astrocytes and microglia maladaptive responses to acute stress are rescued by a single administration of ketamine in a rodent model of PTSD. Transl. Psychiatry 14, 209 (2024). 38796504 10.1038/s41398-024-02928-6 PMC11127980

[10] R. C. Paolicelli, A. Sierra, B. Stevens, M.-E. Tremblay, A. Aguzzi, B. Ajami, I. Amit, E. Audinat, I. Bechmann, M. Bennett, F. Bennett, A. Bessis, K. Biber, S. Bilbo, M. Blurton-Jones, E. Boddeke, D. Brites, B. Brône, G. C. Brown, O. Butovsky, M. J. Carson, B. Castellano, M. Colonna, S. A. Cowley, C. Cunningham, D. Davalos, P. L. D. Jager, B. de Strooper, A. Denes, B. J. L. Eggen, U. Eyo, E. Galea, S. Garel, F. Ginhoux, C. K. Glass, O. Gokce, D. Gomez-Nicola, B. González, S. Gordon, M. B. Graeber, A. D. Greenhalgh, P. Gressens, M. Greter, D. H. Gutmann, C. Haass, M. T. Heneka, F. L. Heppner, S. Hong, D. A. Hume, S. Jung, H. Kettenmann, J. Kipnis, R. Koyama, G. Lemke, M. Lynch, A. Majewska, M. Malcangio, T. Malm, R. Mancuso, T. Masuda, M. Matteoli, B. W. McColl, V. E. Miron, A. V. Molofsky, M. Monje, E. Mracsko, A. Nadjar, J. J. Neher, U. Neniskyte, H. Neumann, M. Noda, B. Peng, F. Peri, V. H. Perry, P. G. Popovich, C. Pridans, J. Priller, M. Prinz, D. Ragozzino, R. M. Ransohoff, M. W. Salter, A. Schaefer, D. P. Schafer, M. Schwartz, M. Simons, C. J. Smith, W. J. Streit, T. L. Tay, L.-H. Tsai, A. Verkhratsky, R. von Bernhardi, H. Wake, V. Wittamer, S. A. Wolf, L.-J. Wu, T. Wyss-Coray, Microglia states and nomenclature: A field at its crossroads. Neuron 110, 3458–3483 (2022). 36327895 10.1016/j.neuron.2022.10.020 PMC9999291

[11] K. Haruwaka, Y. Ying, Y. Liang, A. D. Umpierre, M.-H. Yi, V. Kremen, T. Chen, T. Xie, F. Qi, S. Zhao, J. Zheng, Y. U. Liu, H. Dong, G. A. Worrell, L.-J. Wu, Microglia enhance post-anesthesia neuronal activity by shielding inhibitory synapses. Nat. Neurosci. 27, 449–461 (2024). 38177340 10.1038/s41593-023-01537-8 PMC10960525

[12] Y. U. Liu, Y. Ying, Y. Li, U. B. Eyo, T. Chen, J. Zheng, A. D. Umpierre, J. Zhu, D. B. Bosco, H. Dong, L.-J. Wu, Neuronal network activity controls microglial process surveillance in awake mice via norepinephrine signaling. Nat. Neurosci. 22, 1771–1781 (2019). 31636449 10.1038/s41593-019-0511-3 PMC6858573

[13] C. N. Parkhurst, G. Yang, I. Ninan, J. N. Savas, J. R. Yates, J. J. Lafaille, B. L. Hempstead, D. R. Littman, W.-B. Gan, Microglia promote learning-dependent synapse formation through brain-derived neurotrophic factor. Cell 155, 1596–1609 (2013). 24360280 10.1016/j.cell.2013.11.030 PMC4033691

[14] L. Weinhard, G. di Bartolomei, G. Bolasco, P. Machado, N. L. Schieber, U. Neniskyte, M. Exiga, A. Vadisiute, A. Raggioli, A. Schertel, Y. Schwab, C. T. Gross, Microglia remodel synapses by presynaptic trogocytosis and spine head filopodia induction. Nat. Commun. 9, 1228 (2018). 29581545 10.1038/s41467-018-03566-5 PMC5964317

[15] B. C. Bobotis, O. Braniff, M. Gargus, E. T. Akinluyi, I. O. Awogbindin, M.-È. Tremblay, Sex differences of microglia in the healthy brain from embryonic development to adulthood and across lifestyle influences. Brain Res. Bull. 202, 110752 (2023). 37652267 10.1016/j.brainresbull.2023.110752

[16] D. Guneykaya, A. Ivanov, D. P. Hernandez, V. Haage, B. Wojtas, N. Meyer, M. Maricos, P. Jordan, A. Buonfiglioli, B. Gielniewski, N. Ochocka, C. Cömert, C. Friedrich, L. S. Artiles, B. Kaminska, P. Mertins, D. Beule, H. Kettenmann, S. A. Wolf, Transcriptional and translational differences of microglia from male and female brains. Cell Rep. 24, 2773–2783.e6 (2018). 30184509 10.1016/j.celrep.2018.08.001

[17] M. S. Thion, D. Low, A. Silvin, J. Chen, P. Grisel, J. Schulte-Schrepping, R. Blecher, T. Ulas, P. Squarzoni, G. Hoeffel, F. Coulpier, E. Siopi, F. S. David, C. Scholz, F. Shihui, J. Lum, A. A. Amoyo, A. Larbi, M. Poidinger, A. Buttgereit, P.-M. Lledo, M. Greter, J. K. Y. Chan, I. Amit, M. Beyer, J. L. Schultze, A. Schlitzer, S. Pettersson, F. Ginhoux, S. Garel, Microbiome influences prenatal and adult microglia in a sex-specific manner. Cell 172, 500–516.e16 (2018). 29275859 10.1016/j.cell.2017.11.042 PMC5786503

[18] A. Villa, P. Gelosa, L. Castiglioni, M. Cimino, N. Rizzi, G. Pepe, F. Lolli, E. Marcello, L. Sironi, E. Vegeto, A. Maggi, Sex-specific features of microglia from adult mice. Cell Rep. 23, 3501–3511 (2018). 29924994 10.1016/j.celrep.2018.05.048 PMC6024879

[19] S. L. Klein, K. L. Flanagan, Sex differences in immune responses. Nat. Rev. Immunol. 16, 626–638 (2016). 27546235 10.1038/nri.2016.90

[20] V. Taneja, Sex hormones determine immune response. Front. Immunol. 9, 1931 (2018). 30210492 10.3389/fimmu.2018.01931 PMC6119719

[21] D. A. Bangasser, A. Cuarenta, Sex differences in anxiety and depression: Circuits and mechanisms. Nat. Rev. Neurosci. 22, 674–684 (2021). 34545241 10.1038/s41583-021-00513-0

[22] L. Cahill, Why sex matters for neuroscience. Nat. Rev. Neurosci. 7, 477–484 (2006). 16688123 10.1038/nrn1909

[23] J. D. Gray, J. F. Kogan, J. Marrocco, B. S. McEwen, Genomic and epigenomic mechanisms of glucocorticoids in the brain. Nat. Rev. Endocrinol. 13, 661–673 (2017). 28862266 10.1038/nrendo.2017.97

[24] C. M. Noddings, J. L. Johnson, D. A. Agard, Cryo-EM reveals how Hsp90 and FKBP immunophilins co-regulate the glucocorticoid receptor. Nat. Struct. Mol. Biol. 30, 1867–1877 (2023). 37945740 10.1038/s41594-023-01128-y PMC10716051

[25] A. S. Zannas, T. Wiechmann, N. C. Gassen, E. B. Binder, Gene–stress–epigenetic regulation of FKBP5: Clinical and translational implications. Neuropsychopharmacology 41, 261–274 (2016). 26250598 10.1038/npp.2015.235 PMC4677131

[26] W. Härtig, K. Brauer, G. Brückner, Wisteria floribunda agglutinin-labelled nets surround parvalbumin-containing neurons. Neuroreport 3, 869–872 (1992). 1421090 10.1097/00001756-199210000-00012

[27] A. Miyamoto, H. Wake, A. W. Ishikawa, K. Eto, K. Shibata, H. Murakoshi, S. Koizumi, A. J. Moorhouse, Y. Yoshimura, J. Nabekura, Microglia contact induces synapse formation in developing somatosensory cortex. Nat. Commun. 7, 12540 (2016). 27558646 10.1038/ncomms12540 PMC5007295

[28] F. C. Nebeling, S. Poll, L. C. Justus, J. Steffen, K. Keppler, M. Mittag, M. Fuhrmann, Microglial motility is modulated by neuronal activity and correlates with dendritic spine plasticity in the hippocampus of awake mice. eLife 12, e83176 (2023). 36749020 10.7554/eLife.83176 PMC9946443

[29] K. Bundschu, U. Walter, K. Schuh, Getting a first clue about SPRED functions. Bioessays 29, 897–907 (2007). 17691106 10.1002/bies.20632

[30] T. Wakioka, A. Sasaki, R. Kato, T. Shouda, A. Matsumoto, K. Miyoshi, M. Tsuneoka, S. Komiya, R. Baron, A. Yoshimura, Spred is a Sprouty-related suppressor of Ras signalling. Nature 412, 647–651 (2001). 11493923 10.1038/35088082

[31] D. W. Cain, J. A. Cidlowski, Immune regulation by glucocorticoids. Nat. Rev. Immunol. 17, 233–247 (2017). 28192415 10.1038/nri.2017.1 PMC9761406

[32] D. S. Park, T. Kozaki, S. K. Tiwari, M. Moreira, A. Khalilnezhad, F. Torta, N. Olivié, C. H. Thiam, O. Liani, A. Silvin, W. W. Phoo, L. Gao, A. Triebl, W. K. Tham, L. Gonçalves, W. T. Kong, S. Raman, X. M. Zhang, G. Dunsmore, C. A. Dutertre, S. Lee, J. M. Ong, A. Balachander, S. Khalilnezhad, J. Lum, K. Duan, Z. M. Lim, L. Tan, I. Low, K. H. Utami, X. Y. Yeo, S. Di Tommaso, J.-W. Dupuy, B. Varga, R. T. Karadottir, M. C. Madathummal, I. Bonne, B. Malleret, Z. Y. Binte, N. Wei Da, Y. Tan, W. J. Wong, J. Zhang, J. Chen, R. M. Sobota, S. W. Howland, L. G. Ng, F. Saltel, D. Castel, J. Grill, V. Minard, S. Albani, J. K. Y. Chan, M. S. Thion, S. Y. Jung, M. R. Wenk, M. A. Pouladi, C. Pasqualini, V. Angeli, O. N. F. Cexus, F., Ginhoux, iPS-cell-derived microglia promote brain organoid maturation via cholesterol transfer. Nature 623, 397–405 (2023). 37914940 10.1038/s41586-023-06713-1

[33] J. Ulke, S. Chopra, O. L.-J. Kadiri, P. Geserick, V. Stein, S. Cheshmeh, A. Kleinridders, K. Kappert, PTPRJ is a negative regulator of insulin signaling in neuronal cells, impacting protein biosynthesis, and neurite outgrowth. J. Neuroendocrinol. 36, e13446 (2024). 39253900 10.1111/jne.13446 PMC11646663

[34] A. Roussel-Gervais, C. Couture, D. Langlais, S. Takayasu, A. Balsalobre, B. R. Rueda, L. R. Zukerberg, D. Figarella-Branger, T. Brue, J. Drouin, The cables1 gene in glucocorticoid regulation of pituitary corticotrope growth and cushing disease. J. Clin. Endocrinol. Metab. 101, 513–522 (2016). 26695862 10.1210/jc.2015-3324

[35] A. S. Zannas, M. Jia, K. Hafner, J. Baumert, T. Wiechmann, J. C. Pape, J. Arloth, M. Ködel, S. Martinelli, M. Roitman, S. Röh, A. Haehle, R. T. Emeny, S. Iurato, T. Carrillo-Roa, J. Lahti, K. Räikkönen, J. G. Eriksson, A. J. Drake, M. Waldenberger, S. Wahl, S. Kunze, S. Lucae, B. Bradley, C. Gieger, F. Hausch, A. K. Smith, K. J. Ressler, B. Müller-Myhsok, K.-H. Ladwig, T. Rein, N. C. Gassen, E. B. Binder, Epigenetic upregulation of FKBP5 by aging and stress contributes to NF-κB–driven inflammation and cardiovascular risk. Proc. Natl. Acad. Sci. U.S.A. 116, 11370–11379 (2019). 31113877 10.1073/pnas.1816847116 PMC6561294

[36] I. Bancos, B. A. Hatipoglu, K. C. J. Yuen, L. Chandramohan, S. Chaudhari, A. G. Moraitis, Evaluation of FKBP5 as a cortisol activity biomarker in patients with ACTH-dependent Cushing syndrome. J. Clin. Transl. Endocrinol. 24, 100256 (2021). 34258233 10.1016/j.jcte.2021.100256 PMC8260880

[37] V. Buffa, F. H. Knaup, T. Heymann, M. Springer, M. V. Schmidt, F. Hausch, Analysis of the selective antagonist SAFit2 as a chemical probe for the FK506-binding protein 51. ACS Pharmacol. Transl. Sci. 6, 361–371 (2023). 36926456 10.1021/acsptsci.2c00234 PMC10012253

[38] S. Gaali, A. Kirschner, S. Cuboni, J. Hartmann, C. Kozany, G. Balsevich, C. Namendorf, P. Fernandez-Vizarra, C. Sippel, A. S. Zannas, R. Draenert, E. B. Binder, O. F. X. Almeida, G. Rühter, M. Uhr, M. V. Schmidt, C. Touma, A. Bracher, F. Hausch, Selective inhibitors of the FK506-binding protein 51 by induced fit. Nat. Chem. Biol. 11, 33–37 (2015). 25436518 10.1038/nchembio.1699

[39] A. S. Häusl, L. M. Brix, J. Hartmann, M. L. Pöhlmann, J.-P. Lopez, D. Menegaz, E. Brivio, C. Engelhardt, S. Roeh, T. Bajaj, L. Rudolph, R. Stoffel, K. Hafner, H. M. Goss, J. M. H. M. Reul, J. M. Deussing, M. Eder, K. J. Ressler, N. C. Gassen, A. Chen, M. V. Schmidt, The co-chaperone Fkbp5 shapes the acute stress response in the paraventricular nucleus of the hypothalamus of male mice. Mol. Psychiatry 26, 3060–3076 (2021). 33649453 10.1038/s41380-021-01044-x PMC8505251

[40] J. Eberwine, “Glucocorticoid and mineralocorticoid receptors as transcription Factors,” in *Basic Neurochemistry: Molecular*, *Cellular and Medical Aspects. 6th Edition* (Lippincott-Raven, 1999; https://ncbi.nlm.nih.gov/books/NBK28050/).

[41] K. Vagnerová, M. Jágr, C. Mekadim, P. Ergang, H. Sechovcová, M. Vodička, K. Olša Fliegerová, V. Dvořáček, J. Mrázek, J. Pácha, Profiling of adrenal corticosteroids in blood and local tissues of mice during chronic stress. Sci. Rep. 13, 7278 (2023). 37142643 10.1038/s41598-023-34395-2 PMC10160118

[42] L. A. DeNardo, C. D. Liu, W. E. Allen, E. L. Adams, D. Friedmann, L. Fu, C. J. Guenthner, M. Tessier-Lavigne, L. Luo, Temporal evolution of cortical ensembles promoting remote memory retrieval. Nat. Neurosci. 22, 460–469 (2019). 30692687 10.1038/s41593-018-0318-7 PMC6387639

[43] M. L. Lehmann, R. A. Brachman, K. Martinowich, R. J. Schloesser, M. Herkenham, Glucocorticoids orchestrate divergent effects on mood through adult neurogenesis. J. Neurosci. 33, 2961–2972 (2013). 23407954 10.1523/JNEUROSCI.3878-12.2013 PMC3711562

[44] L. Zhang, R.-R. Lu, R.-H. Xu, H.-H. Wang, W.-S. Feng, X.-K. Zheng, Naringenin and apigenin ameliorates corticosterone-induced depressive behaviors. Heliyon 9, e15618 (2023). 37215924 10.1016/j.heliyon.2023.e15618 PMC10192682

[45] K. A. Kelly, D. B. Miller, J. F. Bowyer, J. P. O’Callaghan, Chronic exposure to corticosterone enhances the neuroinflammatory and neurotoxic responses to methamphetamine. J. Neurochem. 122, 995–1009 (2012). 22776046 10.1111/j.1471-4159.2012.07864.x PMC4706460

[46] E.-L. Sainio, T. Lehtola, P. Roininen, Radioimmunoassay of total and free corticosterone in rat plasma: Measurement of the effect of different doses of corticosterone. Steroids 51, 609–622 (1988). 3242180 10.1016/0039-128x(88)90056-6

[47] R. D. Stowell, G. O. Sipe, R. P. Dawes, H. N. Batchelor, K. A. Lordy, B. S. Whitelaw, M. B. Stoessel, J. M. Bidlack, E. Brown, M. Sur, A. K. Majewska, Noradrenergic signaling in the wakeful state inhibits microglial surveillance and synaptic plasticity in the mouse visual cortex. Nat. Neurosci. 22, 1782–1792 (2019). 31636451 10.1038/s41593-019-0514-0 PMC6875777

[48] Z. Kagiampaki, V. Rohner, C. Kiss, S. Curreli, A. Dieter, M. Wilhelm, M. Harada, S. N. Duss, J. Dernic, M. A. Bhat, X. Zhou, L. Ravotto, T. Ziebarth, L. M. Wasielewski, L. Sönmez, D. Benke, B. Weber, J. Bohacek, A. Reiner, J. S. Wiegert, T. Fellin, T. Patriarchi, Sensitive multicolor indicators for monitoring norepinephrine in vivo. Nat. Methods 20, 1426–1436 (2023). 37474807 10.1038/s41592-023-01959-z PMC7615053

[49] G. L. Quirarte, B. Roozendaal, J. L. McGaugh, Glucocorticoid enhancement of memory storage involves noradrenergic activation in the basolateral amygdala. Proc. Natl. Acad. Sci. U.S.A. 94, 14048–14053 (1997). 9391150 10.1073/pnas.94.25.14048 PMC28430

[50] S. F. Sorrells, J. R. Caso, C. D. Munhoz, R. M. Sapolsky, The stressed CNS: When glucocorticoids aggravate inflammation. Neuron 64, 33–39 (2009). 19840546 10.1016/j.neuron.2009.09.032 PMC4782919

[51] J. Galon, D. Franchimont, N. Hiroi, G. Frey, A. Boettner, M. Ehrhart-Bornstein, J. J. O’shea, G. P. Chrousos, S. R. Bornstein, Gene profiling reveals unknown enhancing and suppressive actions of glucocorticoids on immune cells. FASEB J. 16, 61–71 (2002). 11772937 10.1096/fj.01-0245com

[52] C. L. Storer, C. A. Dickey, M. D. Galigniana, T. Rein, M. B. Cox, FKBP51 and FKBP52 in signaling and disease. Trends Endocrinol. Metab. 22, 481–490 (2011). 21889356 10.1016/j.tem.2011.08.001 PMC3229651

[53] Y. Zhang, K. Chen, S. A. Sloan, M. L. Bennett, A. R. Scholze, S. O’Keeffe, H. P. Phatnani, P. Guarnieri, C. Caneda, N. Ruderisch, S. Deng, S. A. Liddelow, C. Zhang, R. Daneman, T. Maniatis, B. A. Barres, J. Q. Wu, An RNA-sequencing transcriptome and splicing database of glia, neurons, and vascular cells of the cerebral cortex. J. Neurosci. 34, 11929–11947 (2014). 25186741 10.1523/JNEUROSCI.1860-14.2014 PMC4152602

[54] S. Siegert, E. Cabuy, B. G. Scherf, H. Kohler, S. Panda, Y.-Z. Le, H. J. Fehling, D. Gaidatzis, M. B. Stadler, B. Roska, Transcriptional code and disease map for adult retinal cell types. Nat. Neurosci. 15, 487–495 (2012). 22267162 10.1038/nn.3032

[55] P. T. Nguyen, L. C. Dorman, S. Pan, I. D. Vainchtein, R. T. Han, H. Nakao-Inoue, S. E. Taloma, J. J. Barron, A. B. Molofsky, M. A. Kheirbek, A. V. Molofsky, Microglial remodeling of the extracellular matrix promotes synapse plasticity. Cell 182, 388–403.e15 (2020). 32615087 10.1016/j.cell.2020.05.050 PMC7497728

[56] V. Nold, M. Portenhauser, D. Del Prete, A. Blasius, I. Harris, E. Koros, T. Peleh, B. Hengerer, I.-T. Kolassa, M. Slezak, K. A. Allers, Impact of Fkbp5 × early life adversity × sex in humanised mice on multidimensional stress responses and circadian rhythmicity. Mol. Psychiatry 27, 3544–3555 (2022). 35449298 10.1038/s41380-022-01549-z PMC9708571

[57] L. van Doeselaar, T. Stark, S. Mitra, H. Yang, J. Bordes, L. Stolwijk, C. Engelhardt, V. Kovarova, S. Narayan, L. M. Brix, M. Springer, J. M. Deussing, J. P. Lopez, M. Czisch, M. V. Schmidt, Sex-specific and opposed effects of FKBP51 in glutamatergic and GABAergic neurons: Implications for stress susceptibility and resilience. Proc. Natl. Acad. Sci U.S.A. 120, e2300722120 (2023). 37252963 10.1073/pnas.2300722120 PMC10266018

[58] P. Zanos, R. Moaddel, P. J. Morris, P. Georgiou, J. Fischell, G. I. Elmer, M. Alkondon, P. Yuan, H. J. Pribut, N. S. Singh, K. S. S. Dossou, Y. Fang, X.-P. Huang, C. L. Mayo, I. W. Wainer, E. X. Albuquerque, S. M. Thompson, C. J. Thomas, C. A. Zarate Jr., T. D. Gould, NMDAR inhibition-independent antidepressant actions of ketamine metabolites. Nature 533, 481–486 (2016). 27144355 10.1038/nature17998 PMC4922311

[59] H. E. Braithwaite, T. Payne, N. Duce, J. Lim, T. McCulloch, J. Loadsman, K. Leslie, A. C. Webster, A. Gaskell, R. D. Sanders, Impact of female sex on anaesthetic awareness, depth, and emergence: A systematic review and meta-analysis. Br. J. Anaesth. 131, 510–522 (2023). 37453840 10.1016/j.bja.2023.06.042

[60] A. Z. Wasilczuk, C. Rinehart, A. Aggarwal, M. E. Stone, G. A. Mashour, M. S. Avidan, M. B. Kelz, A. Proekt, ReCCognition Study Group, Hormonal basis of sex differences in anesthetic sensitivity. Proc. Natl. Acad. Sci U.S.A. 121, e2312913120 (2024). 38190526 10.1073/pnas.2312913120 PMC10801881

[61] J. A. Da Silva, Sex hormones and glucocorticoids: Interactions with the immune system. Ann. N. Y. Acad. Sci. 876, 102–118 (1999). 10415599 10.1111/j.1749-6632.1999.tb07628.x

[62] L. Miller, J. S. Hunt, Sex steroid hormones and macrophage function. Life Sci. 59, 1–14 (1996). 8684265 10.1016/0024-3205(96)00122-1

[63] H. J. Krugers, H. Karst, M. Joels, Interactions between noradrenaline and corticosteroids in the brain: From electrical activity to cognitive performance. Front. Cell. Neurosci. 6, 10.3389/fncel.2012.00015 (2012). 10.3389/fncel.2012.00015 PMC3321636 22509154

[64] R. Schulz, M. Korkut-Demirbaş, A. Venturino, G. Colombo, S. Siegert, Chimeric GPCRs mimic distinct signaling pathways and modulate microglia responses. Nat. Commun. 13, 4728 (2022). 35970889 10.1038/s41467-022-32390-1 PMC9378622

[65] J. O’Donnell, D. Zeppenfeld, E. McConnell, S. Pena, M. Nedergaard, Norepinephrine: A neuromodulator that boosts the function of multiple cell types to optimize CNS performance. Neurochem. Res. 37, 2496–2512 (2012). 22717696 10.1007/s11064-012-0818-x PMC3548657

[66] R. A. España, B. E. Schmeichel, C. W. Berridge, Norepinephrine at the nexus of arousal, motivation and relapse. Brain Res. 1641, 207–216 (2016). 26773688 10.1016/j.brainres.2016.01.002 PMC4879075

[67] K. Hohlbaum, B. Bert, S. Dietze, R. Palme, H. Fink, C. Thöne-Reineke, Severity classification of repeated isoflurane anesthesia in C57BL/6JRj mice—Assessing the degree of distress. PLOS ONE 12, e0179588 (2017). 28617851 10.1371/journal.pone.0179588 PMC5472303

[68] M. Duque, A. B. Chen, E. Hsu, S. Narayan, A. Rymbek, S. Begum, G. Saher, A. E. Cohen, D. E. Olson, Y. Li, D. A. Prober, D. E. Bergles, M. C. Fishman, F. Engert, M. B. Ahrens, Ketamine induces plasticity in a norepinephrine-astroglial circuit to promote behavioral perseverance. Neuron 113, 426–443.e5 (2025). 39694033 10.1016/j.neuron.2024.11.011 PMC11889991

[69] M. Paukert, A. Agarwal, J. Cha, V. A. Doze, J. U. Kang, D. E. Bergles, Norepinephrine controls astroglial responsiveness to local circuit activity. Neuron 82, 1263–1270 (2014). 24945771 10.1016/j.neuron.2014.04.038 PMC4080721

[70] S. A. Liddelow, K. A. Guttenplan, L. E. Clarke, F. C. Bennett, C. J. Bohlen, L. Schirmer, M. L. Bennett, A. E. Münch, W.-S. Chung, T. C. Peterson, D. K. Wilton, A. Frouin, B. A. Napier, N. Panicker, M. Kumar, M. S. Buckwalter, D. H. Rowitch, V. L. Dawson, T. M. Dawson, B. Stevens, B. A. Barres, Neurotoxic reactive astrocytes are induced by activated microglia. Nature 541, 481–487 (2017). 28099414 10.1038/nature21029 PMC5404890

[71] Y. Zhan, R. C. Paolicelli, F. Sforazzini, L. Weinhard, G. Bolasco, F. Pagani, A. L. Vyssotski, A. Bifone, A. Gozzi, D. Ragozzino, C. T. Gross, Deficient neuron-microglia signaling results in impaired functional brain connectivity and social behavior. Nat. Neurosci. 17, 400–406 (2014). 24487234 10.1038/nn.3641

[72] J. T. Rogers, J. M. Morganti, A. D. Bachstetter, C. E. Hudson, M. M. Peters, B. A. Grimmig, E. J. Weeber, P. C. Bickford, C. Gemma, CX3CR1 deficiency leads to impairment of hippocampal cognitive function and synaptic plasticity. J. Neurosci. 31, 16241–16250 (2011). 22072675 10.1523/JNEUROSCI.3667-11.2011 PMC3236509

[73] S. Gyoneva, R. Hosur, D. Gosselin, B. Zhang, Z. Ouyang, A. C. Cotleur, M. Peterson, N. Allaire, R. Challa, P. Cullen, C. Roberts, K. Miao, T. L. Reynolds, C. K. Glass, L. Burkly, R. M. Ransohoff, Cx3cr1-deficient microglia exhibit a premature aging transcriptome. Life Sci. Alliance 2, e201900453 (2019). 31792059 10.26508/lsa.201900453 PMC6892408

[74] G. Corsi, K. Picard, M. A. di Castro, S. Garofalo, F. Tucci, G. Chece, C. del Percio, M. T. Golia, M. Raspa, F. Scavizzi, F. Decoeur, C. Lauro, M. Rigamonti, F. Iannello, D. A. Ragozzino, E. Russo, G. Bernardini, A. Nadjar, M. E. Tremblay, C. Babiloni, L. Maggi, C. Limatola, Microglia modulate hippocampal synaptic transmission and sleep duration along the light/dark cycle. Glia 70, 89–105 (2022). 34487590 10.1002/glia.24090 PMC9291950

[75] L. Chappell-Maor, M. Kolesnikov, J.-S. Kim, A. Shemer, Z. Haimon, J. Grozovski, S. Boura-Halfon, T. Masuda, M. Prinz, S. Jung, Comparative analysis of CreER transgenic mice for the study of brain macrophages: A case study. Eur. J. Immunol. 50, 353–362 (2020). 31762013 10.1002/eji.201948342

[76] T. E. Faust, P. A. Feinberg, C. O’Connor, R. Kawaguchi, A. Chan, H. Strasburger, M. Frosch, M. A. Boyle, T. Masuda, L. Amann, K.-P. Knobeloch, M. Prinz, A. Schaefer, D. P. Schafer, A comparative analysis of microglial inducible Cre lines. Cell Rep. 42, 113031 (2023). 37635351 10.1016/j.celrep.2023.113031 PMC10591718

[77] A. M. Bedolla, G. L. McKinsey, K. Ware, N. Santander, T. D. Arnold, Y. Luo, A comparative evaluation of the strengths and potential caveats of the microglial inducible CreER mouse models. Cell Rep. 43, 113660 (2024). 38217856 10.1016/j.celrep.2023.113660 PMC10874587

[78] S. Yona, K.-W. Kim, Y. Wolf, A. Mildner, D. Varol, M. Breker, D. Strauss-Ayali, S. Viukov, M. Guilliams, A. Misharin, D. A. Hume, H. Perlman, B. Malissen, E. Zelzer, S. Jung, Fate mapping reveals origins and dynamics of monocytes and tissue macrophages under homeostasis. Immunity 38, 79–91 (2013). 23273845 10.1016/j.immuni.2012.12.001 PMC3908543

[79] L. Madisen, T. A. Zwingman, S. M. Sunkin, S. W. Oh, H. A. Zariwala, H. Gu, L. L. Ng, R. D. Palmiter, M. J. Hawrylycz, A. R. Jones, E. S. Lein, H. Zeng, A robust and high-throughput Cre reporting and characterization system for the whole mouse brain. Nat. Neurosci. 13, 133–140 (2010). 20023653 10.1038/nn.2467 PMC2840225

[80] G. Feng, R. H. Mellor, M. Bernstein, C. Keller-Peck, Q. T. Nguyen, M. Wallace, J. M. Nerbonne, J. W. Lichtman, J. R. Sanes, Imaging neuronal subsets in transgenic mice expressing multiple spectral variants of GFP. Neuron 28, 41–51 (2000). 11086982 10.1016/s0896-6273(00)00084-2

[81] C. Scheiermann, Y. Kunisaki, P. S. Frenette, Circadian control of the immune system. Nat. Rev. Immunol. 13, 190–198 (2013). 23391992 10.1038/nri3386 PMC4090048

[82] F. Spiga, J. J. Walker, J. R. Terry, S. L. Lightman, “HPA axis-rhythms,” in *Comprehensive Physiology* (John Wiley & Sons Ltd., 2014; https://onlinelibrary.wiley.com/doi/abs/10.1002/cphy.c140003), pp. 1273–1298. 10.1002/cphy.c140003 24944037

[83] M. Arras, P. Autenried, A. Rettich, D. Spaeni, T. Rülicke, Optimization of intraperitoneal injection anesthesia in mice: Drugs, dosages, adverse effects, and anesthesia depth. Comp. Med. 51, 443–456 (2001). 11924805

[84] W. E. Allen, L. A. DeNardo, M. Z. Chen, C. D. Liu, K. M. Loh, L. E. Fenno, C. Ramakrishnan, K. Deisseroth, L. Luo, Thirst-associated preoptic neurons encode an aversive motivational drive. Science 357, 1149–1155 (2017). 28912243 10.1126/science.aan6747 PMC5723384

[85] N. N. Dagher, A. R. Najafi, K. M. N. Kayala, M. R. P. Elmore, T. E. White, R. Medeiros, B. L. West, K. N. Green, Colony-stimulating factor 1 receptor inhibition prevents microglial plaque association and improves cognition in 3xTg-AD mice. J. Neuroinflammation 12, 139 (2015). 26232154 10.1186/s12974-015-0366-9 PMC4522109

[86] E. Spangenberg, P. L. Severson, L. A. Hohsfield, J. Crapser, J. Zhang, E. A. Burton, Y. Zhang, W. Spevak, J. Lin, N. Y. Phan, G. Habets, A. Rymar, G. Tsang, J. Walters, M. Nespi, P. Singh, S. Broome, P. Ibrahim, C. Zhang, G. Bollag, B. L. West, K. N. Green, Sustained microglial depletion with CSF1R inhibitor impairs parenchymal plaque development in an Alzheimer’s disease model. Nat. Commun. 10, 3758 (2019). 31434879 10.1038/s41467-019-11674-z PMC6704256

[87] Y.-J. Liu, K. N. Green, T. C. Holmes, X. Xu, Commentary: How do microglia regulate neural circuit connectivity and activity in the adult brain? Neurosci. Insights 17, 26331055211071124 (2022). 35098130 10.1177/26331055211071124 PMC8796061

[88] Y.-J. Liu, E. E. Spangenberg, B. Tang, T. C. Holmes, K. N. Green, X. Xu, Microglia elimination increases neural circuit connectivity and activity in adult mouse cortex. J. Neurosci. 41, 1274–1287 (2021). 33380470 10.1523/JNEUROSCI.2140-20.2020 PMC7888230

[89] B. Basilico, L. Ferrucci, A. Khan, S. Di Angelantonio, D. Ragozzino, I. Reverte, What microglia depletion approaches tell us about the role of microglia on synaptic function and behavior. Front. Cell. Neurosci. 16, 1022431 (2022). 36406752 10.3389/fncel.2022.1022431 PMC9673171

[90] K. Takeda, M. Pokorski, Y. Sato, Y. Oyamada, Y. Okada, “Respiratory toxicity of dimethyl sulfoxide,” in *Respirology*, M. Pokorski, Ed. (Springer International Publishing, 2016; 10.1007/5584_2015_187), pp. 89–96. 26747070

[91] E. L. van Donkelaar, K. R. D. Vaessen, J. L. Pawluski, A. S. Sierksma, A. Blokland, R. Cañete, H. W. M. Steinbusch, Long-term corticosterone exposure decreases insulin sensitivity and induces depressive-like behaviour in the C57BL/6NCrl mouse. PLOS ONE 9, e106960 (2014). 25310187 10.1371/journal.pone.0106960 PMC4195581

[92] M. G. Codagnone, N. Kara, A. Ratsika, B. R. Levone, M. van de Wouw, L. A. Tan, J. I. Cunningham, C. Sanchez, J. F. Cryan, O. F. O’Leary, Inhibition of FKBP51 induces stress resilience and alters hippocampal neurogenesis. Mol. Psychiatry 27, 4928–4938 (2022). 36104438 10.1038/s41380-022-01755-9 PMC9763121

[93] A. Tsukamoto, K. Serizawa, R. Sato, J. Yamazaki, T. Inomata, Vital signs monitoring during injectable and inhalant anesthesia in mice. Exp. Anim. 64, 57–64 (2015). 25312399 10.1538/expanim.14-0050 PMC4329516

[94] K. Zhang, Y. Fujita, K. Hashimoto, Lack of metabolism in (R)-ketamine’s antidepressant actions in a chronic social defeat stress model. Sci. Rep. 8, 4007 (2018). 29507385 10.1038/s41598-018-22449-9 PMC5838158

[95] J. Bauer, T. Sminia, F. G. Wouterlood, C. D. Dijkstra, Phagocytic activity of macrophages and microglial cells during the course of acute and chronic relapsing experimental autoimmune encephalomyelitis. J. Neurosci. Res. 38, 365–375 (1994). 7932870 10.1002/jnr.490380402

[96] D. Ito, Y. Imai, K. Ohsawa, K. Nakajima, Y. Fukuuchi, S. Kohsaka, Microglia-specific localisation of a novel calcium binding protein, Iba1. Mol. Brain Res. 57, 1–9 (1998). 9630473 10.1016/s0169-328x(98)00040-0

[97] S. G. Owe, A. Erisir, P. Heggelund, Terminals of the major thalamic input to visual cortex are devoid of synapsin proteins. Neuroscience 243, 115–125 (2013). 23535254 10.1016/j.neuroscience.2013.03.031 PMC3953556

[98] E. W. Stockley, H. M. Cole, A. D. Brown, H. V. Wheal, A system for quantitative morphological measurement and electrotonic modelling of neurons: Three-dimensional reconstruction. J. Neurosci. Methods 47, 39–51 (1993). 8321013 10.1016/0165-0270(93)90020-r

[99] L. Kanari, P. Dłotko, M. Scolamiero, R. Levi, J. Shillcock, K. Hess, H. Markram, A topological representation of branching neuronal morphologies. Neuroinformatics 16, 3–13 (2018). 28975511 10.1007/s12021-017-9341-1 PMC5797226

[100] H. Adams, S. Chepushtanova, T. Emerson, E. Hanson, M. Kirby, F. Motta, R. Neville, C. Peterson, P. Shipman, L. Ziegelmeier, Persistence images: A stable vector representation of persistent homology. arXiv:1507.06217 [cs.CG] (2016).

[101] D. P. Kingma, M. Welling, Auto-encoding variational Bayes. arXiv:1312.6114 [stat.ML] (2022).

[102] K. Pearson, On lines and planes of closest fit to systems of points in space. Lond. Edinb. Dublin Philos. Mag. J. Sci. 2, 559–572 (1901).

[103] G. Klambauer, T. Unterthiner, A. Mayr, S. Hochreiter, Self-normalizing neural networks. arXiv:1706.02515 [cs.LG] (2017).

[104] F. Orabona, T. Tommasi, Training deep networks without learning rates through coin betting. arXiv:1705.07795 [cs.LG] (2017).

[105] M. Mori, A. Rosko, J. Farnsworth, G. Carrasco, P. Broomandkhoshbacht, K. Pareja-Navarro, A. P. Haghighi, SimplyFire: An open-source, customizable software application for the analysis of synaptic events. eNeuro 11, ENEURO.0326-23 (2024). 10.1523/ENEURO.0326-23.2023 PMC10849045 38167616

[106] M. M. Koletar, A. Dorr, M. E. Brown, J. McLaurin, B. Stefanovic, Refinement of a chronic cranial window implant in the rat for longitudinal in vivo two–photon fluorescence microscopy of neurovascular function. Sci. Rep. 9, 5499 (2019). 30940849 10.1038/s41598-019-41966-9 PMC6445076

[107] S. W. Cramer, R. E. Carter, J. D. Aronson, S. B. Kodandaramaiah, T. J. Ebner, C. C. Chen, Through the looking glass: A review of cranial window technology for optical access to the brain. J. Neurosci. Methods 354, 109100 (2021). 33600850 10.1016/j.jneumeth.2021.109100 PMC8100903

[108] A. Holtmaat, T. Bonhoeffer, D. K. Chow, J. Chuckowree, V. De Paola, S. B. Hofer, M. Hübener, T. Keck, G. Knott, W.-C. A. Lee, R. Mostany, T. D. Mrsic-Flogel, E. Nedivi, C. Portera-Cailliau, K. Svoboda, J. T. Trachtenberg, L. Wilbrecht, Long-term, high-resolution imaging in the mouse neocortex through a chronic cranial window. Nat. Protoc. 4, 1128–1144 (2009). 19617885 10.1038/nprot.2009.89 PMC3072839

[109] T. Stuart, A. Butler, P. Hoffman, C. Hafemeister, E. Papalexi, W. M. Mauck, Y. Hao, M. Stoeckius, P. Smibert, R. Satija, Comprehensive integration of single-cell data. Cell 177, 1888–1902.e21 (2019). 31178118 10.1016/j.cell.2019.05.031 PMC6687398

[110] P.-L. Germain, A. Lun, C. G. Meixide, W. Macnair, M. D. Robinson, Doublet identification in single-cell sequencing data using *scDblFinder*. F1000Research 10, 979 (2021). 35814628 10.12688/f1000research.73600.1 PMC9204188

[111] D. Aran, A. P. Looney, L. Liu, E. Wu, V. Fong, A. Hsu, S. Chak, R. P. Naikawadi, P. J. Wolters, A. R. Abate, A. J. Butte, M. Bhattacharya, Reference-based analysis of lung single-cell sequencing reveals a transitional profibrotic macrophage. Nat. Immunol. 20, 163–172 (2019). 30643263 10.1038/s41590-018-0276-y PMC6340744

[112] B. Tasic, Z. Yao, L. T. Graybuck, K. A. Smith, T. N. Nguyen, D. Bertagnolli, J. Goldy, E. Garren, M. N. Economo, S. Viswanathan, O. Penn, T. Bakken, V. Menon, J. Miller, O. Fong, K. E. Hirokawa, K. Lathia, C. Rimorin, M. Tieu, R. Larsen, T. Casper, E. Barkan, M. Kroll, S. Parry, N. V. Shapovalova, D. Hirschstein, J. Pendergraft, H. A. Sullivan, T. K. Kim, A. Szafer, N. Dee, P. Groblewski, I. Wickersham, A. Cetin, J. A. Harris, B. P. Levi, S. M. Sunkin, L. Madisen, T. L. Daigle, L. Looger, A. Bernard, J. Phillips, E. Lein, M. Hawrylycz, K. Svoboda, A. R. Jones, C. Koch, H. Zeng, Shared and distinct transcriptomic cell types across neocortical areas. Nature 563, 72–78 (2018). 30382198 10.1038/s41586-018-0654-5 PMC6456269

[113] F. W. Townes, S. C. Hicks, M. J. Aryee, R. A. Irizarry, Feature selection and dimension reduction for single-cell RNA-seq based on a multinomial model. Genome Biol. 20, 295 (2019). 31870412 10.1186/s13059-019-1861-6 PMC6927135

[114] D. Bates, M. Mächler, B. Bolker, S. Walker, Fitting linear mixed-effects models using lme4. arXiv:1406.5823 [stat.CO] (2014).

