# Supplementary material for: Corticosterone-linked microglial activity underpins sexually dimorphic neuroplasticity after ketamine anesthesia
Source: Sci Adv. 2026 Jul 31;12(31):eadz6517. doi: 10.1126/sciadv.adz6517 (PMC13426458; doi:10.1126/sciadv.adz6517)
Supplement: Supplementary file 1 — Figs. S1 to S14 Legends for movies S1 to S4 Legend for table S1 [file sciadv.adz6517_sm.pdf]

Science Advances
AAAS

Supplementary Materials for

Corticosterone-linked microglial activity underpins sexually dimorphic neuroplasticity after ketamine anesthesia

Alessandro Venturino *et al.*

Corresponding author: Sandra Siegert, ssiegert@ista.ac.at

Sci. Adv. **12**, eadz6517 (2026)
DOI: 10.1126/sciadv.adz6517

The PDF file includes:

Figs. S1 to S14
Legends for movies S1 to S4
Legend for table S1

Other Supplementary Material for this manuscript includes the following:

Movies S1 to S4
Table S1

Figure S1

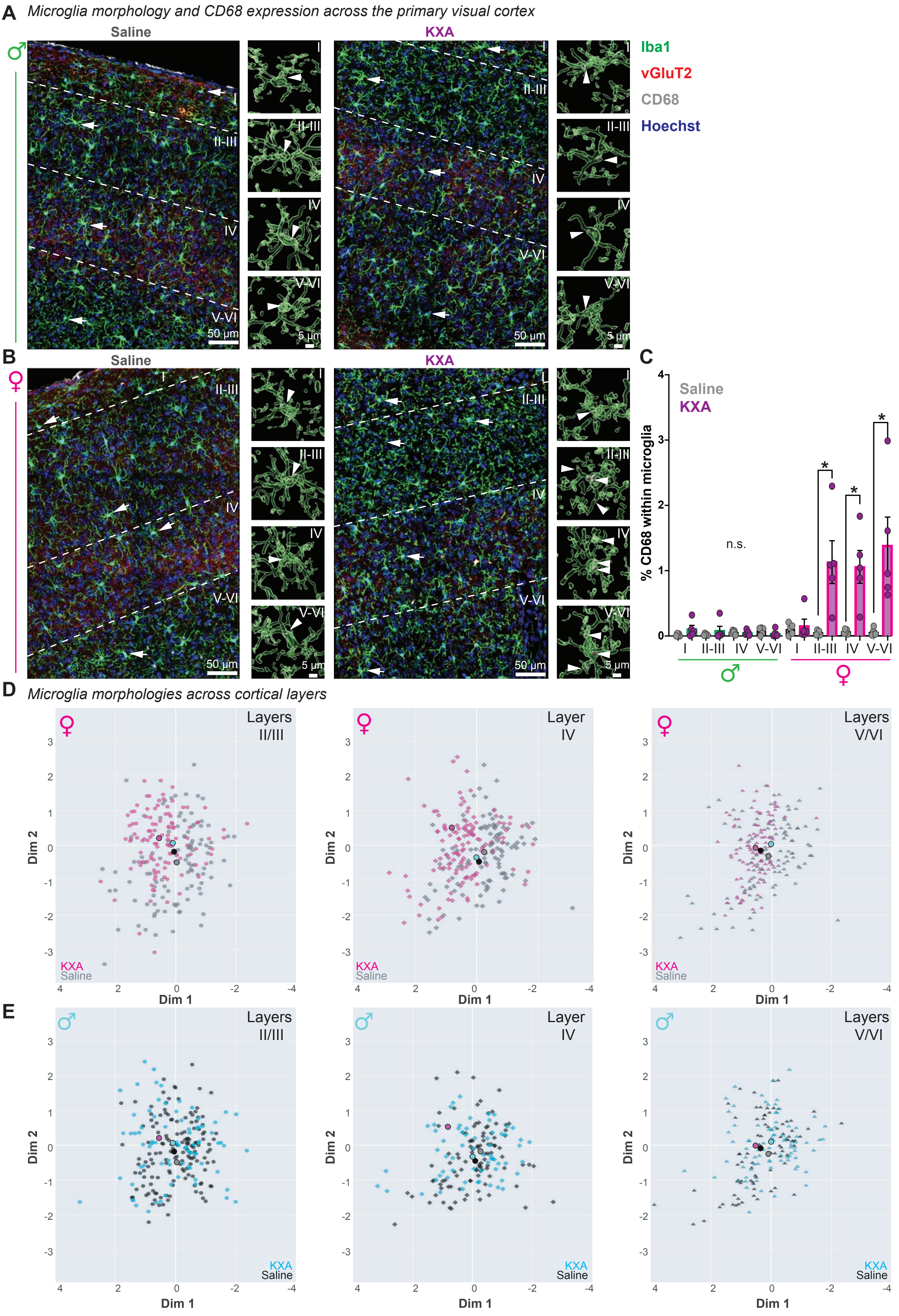

A
Microglia morphology and CD68 expression across the primary visual cortex
Saline
KXA
Iba1
vGluT2
CD68
Hoechst
I
II-III
IV
V-VI
50 µm
5 µm
B
C
Saline
KXA
% CD68 within microglia
n.s.
*
D
Microglia morphologies across cortical layers
Layers II/III
Layer IV
Layers V/VI
Dim 1
Dim 2
E

Figure S1. CD68 expression across VISp cortical layers and microglia morphological adjustment 4h after ketamine anesthesia in males and females.

(**A**-**C**) CD68 quantification across the primary visual cortex (VISp) of males (**A**) and females (**B**), 4 hours after saline or KXA (ketamine-xylazine-acepromacine) injection. (**A**-**B**) Representative immunostainings for Iba1 (green), CD68 (magenta), vGluT2 (red), counterstained with the nuclei-dye Hoechst (blue). Left, overview image of the cortical layers assigned based on the vGluT2 signal. Arrow in each layer, microglia chosen for 3D surface rendering, shown next to the overview. Arrowhead, CD68 location within microglia. Scale bars: 50 µm for overview and 5 µm for rendering. (**C**) Bar chart of the mean percentage of CD68 volume within microglia in each cortical layer with ± SEM. Each dot, one animal. 5 animals/condition. Kruskal-Wallis with Dunn's multiple comparison post hoc test, $^{*}p < 0.05$, $^{ns}p > 0.05$, not significant. (**D**-**E**) Morphological analysis of female (**D**) and male (**E**) microglia within cortical layer II/III, IV, and V/VI in VISp after saline (grey, black) or KXA (magenta, cyan) using morphOMICs. Each microglia's persistence image was embedded in a latent space using a Variational Autoencoder (VAE, see Methods). Larger dots, population mean for sex and condition.

Figure S2

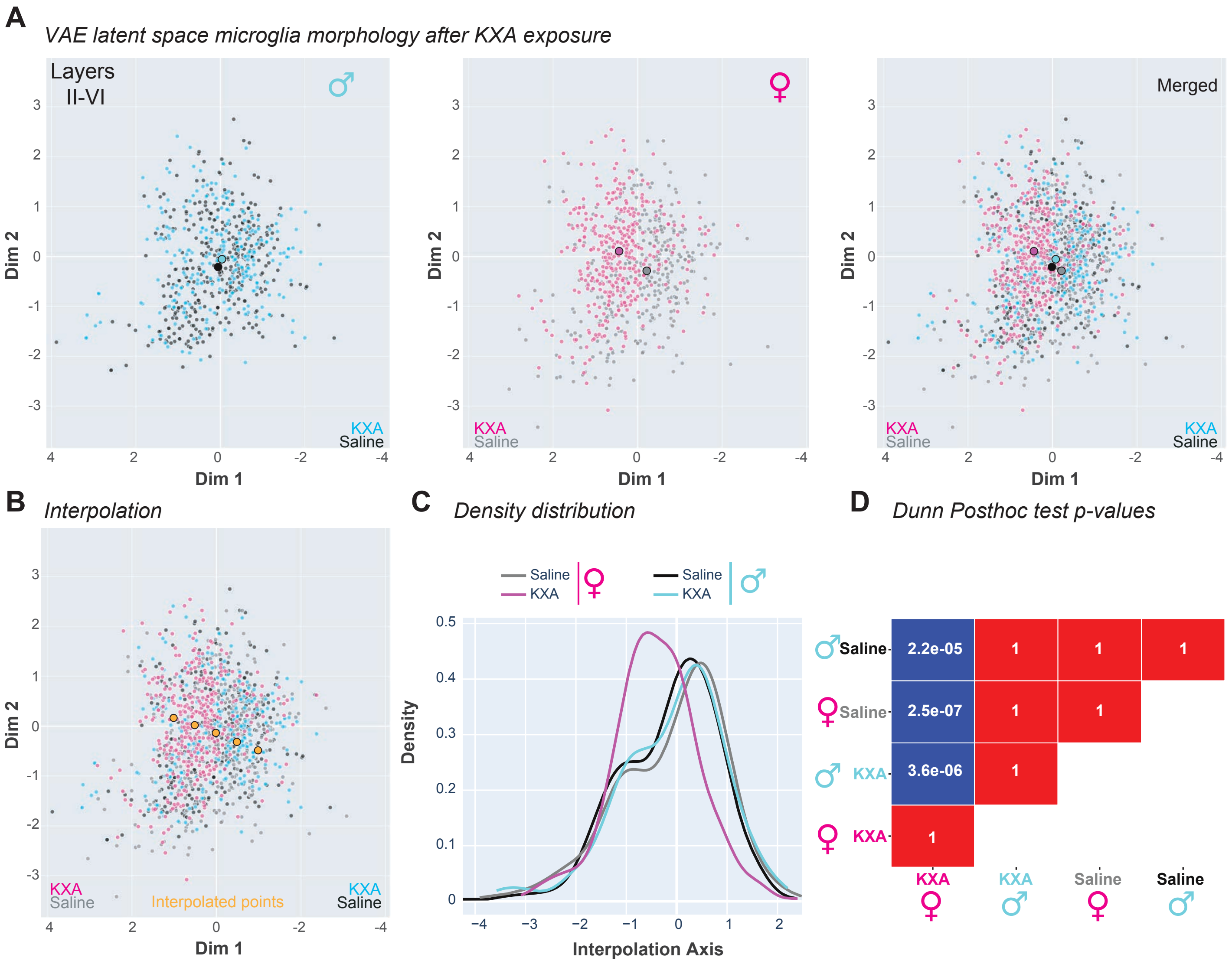
A
VAE latent space microglia morphology after KXA exposure
Layers II-VI
Merged
Dim 1
Dim 2
KXA
Saline
B
Interpolation
Interpolated points
C
Density distribution
Saline
KXA
Density
Interpolation Axis
D
Dunn Posthoc test p-values
Saline
KXA
2.2e-05
2.5e-07
3.6e-06
1

Figure S2. Sex-specific morphological differences in microglia 4h after ketamine anesthesia.

(**A**-**D**) Morphological comparison between male (cyan) and female (magenta) microglia across the cortical layers II-VI in the primary visual cortex (VISp) after saline (grey, black) or KXA (magenta, cyan) using morphOMICs. (**A**) Variational Autoencoder (VAE) latent space representation. Each dot, a single persistence image. Larger dots, population median for sex and condition, color-coded by group conditions. (**B**) One-dimensional projection of the density distribution of embedded persistence images along the interpolation axis. (**C**) Density distribution computed by projecting the embedded persistence images along the interpolation axis (**B**), giving a one-dimensional projection. Color-coded by group conditions. The distribution shift highlights the distinctiveness of the female KXA group relative to the others. (**D**) Statistical comparison of microglia populations using Shapiro-Wilk with Dunn's post hoc test with *p*-values.

Figure S3

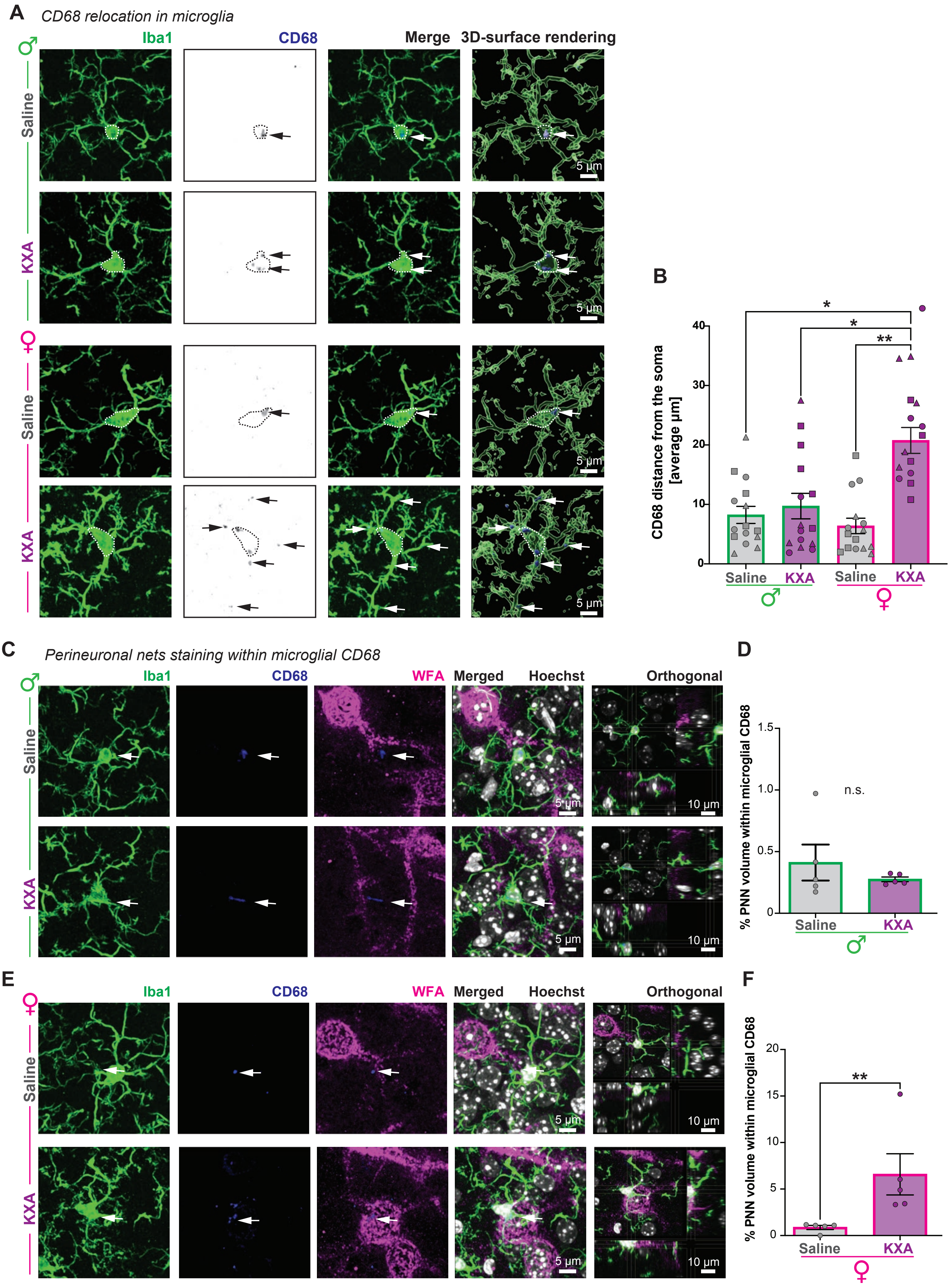
A
CD68 relocation in microglia
Iba1
CD68
Merge
3D-surface rendering
Saline
KXA
5 µm
B
CD68 distance from the soma [average µm]
40
30
20
10
0
*
*
**
Saline
KXA
Saline
KXA
C
Perineuronal nets staining within microglial CD68
Iba1
CD68
WFA
Merged
Hoechst
Orthogonal
5 µm
10 µm
D
% PNN volume within microglial CD68
1.5
1.0
0.5
0
n.s.
Saline
KXA
E
Iba1
CD68
WFA
Merged
Hoechst
Orthogonal
5 µm
10 µm
F
% PNN volume within microglial CD68
20
15
10
5
0
**
Saline
KXA

Figure S3. KXA stimulates female microglia to relocate CD68, which colabels with perineuronal nets (PNN) staining.

(**A**-**F**) Immunostainings for microglia with Iba1 (green) and CD68 (blue) in the primary visual cortex (VISp), layer III-V of males and females, 4 hours after saline or KXA (ketamine-xylazine-acepromazine) injection. (**A**-**B**) Comparison of CD68 relocation within microglia between males (green) and females (magenta). (**A**) Representative immunostainings across sex and condition. Dashed circle, contour of microglial soma as reference. Arrow, CD68 location within microglia. Next to merged images: 3D surface rendering. Scale bar: 5 µm. (**B**) Bar chart of average CD68 distance from the microglia soma with ± SEM. Each dot, a cell with 5 cells/animal. Each symbol: 1 animal per condition. Data have been nested for statistical analysis. One-way nested ANOVA with selected Tukey's multiple comparisons post hoc test, *$p < 0.05$, **$p < 0.01$. (**C**-**F**) Comparison of perineuronal nets staining inside microglia CD68 between males (green, **C**-**D**) and females (magenta, **E**-**F**). Note: Saline male is identical to (**A**), including also WFA. (**C**, **E**) Representative immunostainings across sex and condition, including *Wisteria floribunda agglutinin* (WFA, magenta) staining for perineuronal nets (PNN), and counterstained with the nuclei-dye Hoechst (white). Arrow, CD68 inside microglia. Scale bars: 5 µm and 10 µm for the orthogonal projection. (**D**, **F**) Bar charts of the mean percentage of PNN volume within microglial CD68. Each dot, one animal. 5 animals/condition. (**D**) Unpaired t-test with Welch's correction. $^{ns}p > 0.05$, not significant. (**F**) Mann-Whitney test, **$p < 0.01$.

Figure S4

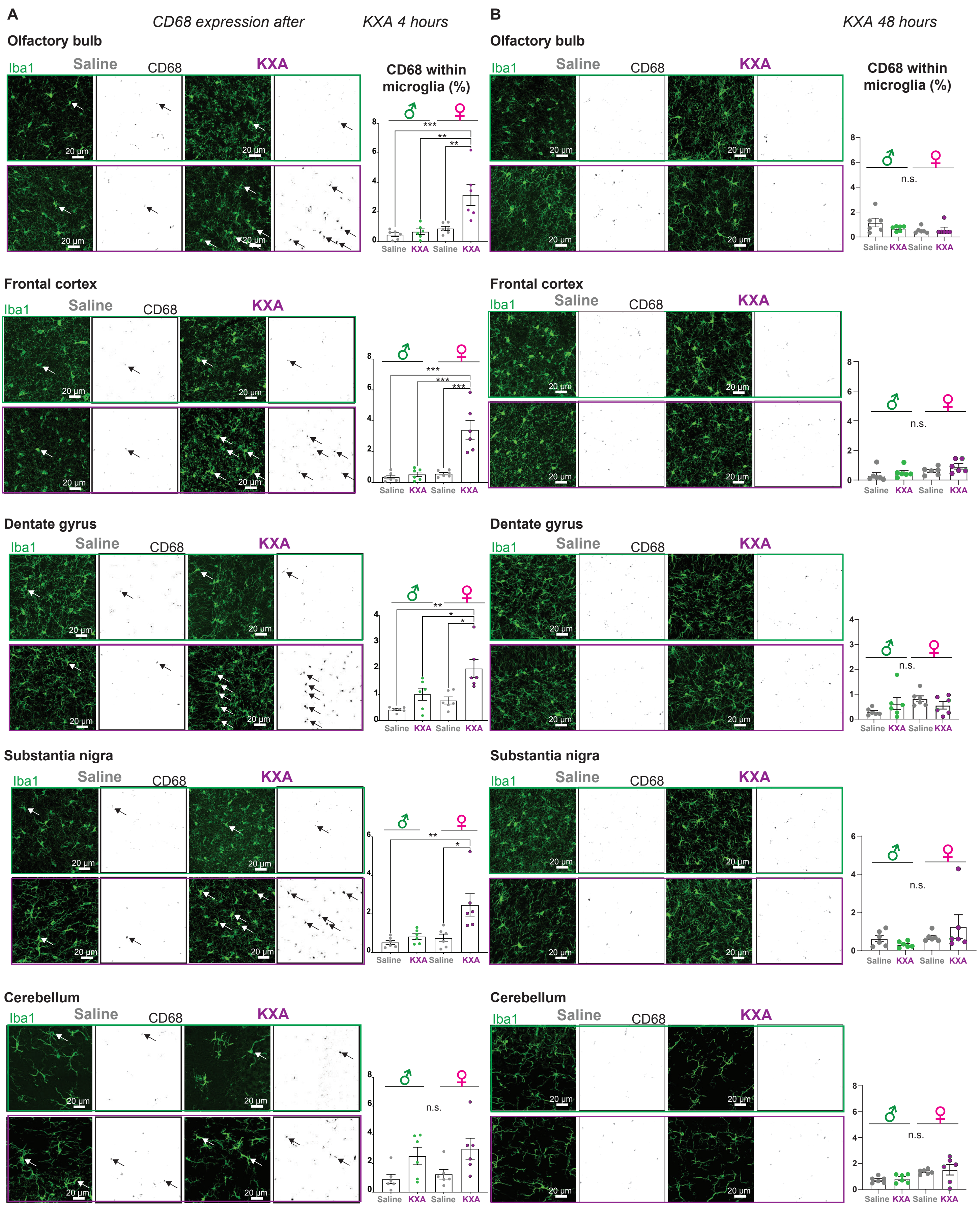
A
CD68 expression after
KXA 4 hours
B
KXA 48 hours
Olfactory bulb
Frontal cortex
Dentate gyrus
Substantia nigra
Cerebellum
Iba1
Saline
CD68
KXA
20 µm
CD68 within microglia (%)
♂
♀
n.s.
Saline
KXA

Figure S4. Microglial CD68 expression across multiple brain regions 4 and 48 hours after KXA anesthesia.

(**A-B**) Representative immunostainings for microglia with Iba1 (green) and CD68 (black) in the olfactory bulb, frontal cortex, dentate gyrus, substantia nigra, and cerebellum of males and females, measured either 4 (**A**) or 48 (**B**) hours after saline or KXA injection. Arrow, CD68 location within microglia. Scale bar: 20 µm. Next, bar charts of the mean percentage ± SEM of CD68 volume within microglia. Each dot, one animal. 6 animals/condition. Kruskal-Wallis with selected Dunn's multiple comparison post hoc test, $^{*}p < 0.05$, $^{**}p < 0.01$, $^{***}p < 0.001$, $^{ns}p > 0.05$, not significant.

Figure S5

CD68 expression after *Isoflurane 4 hours*

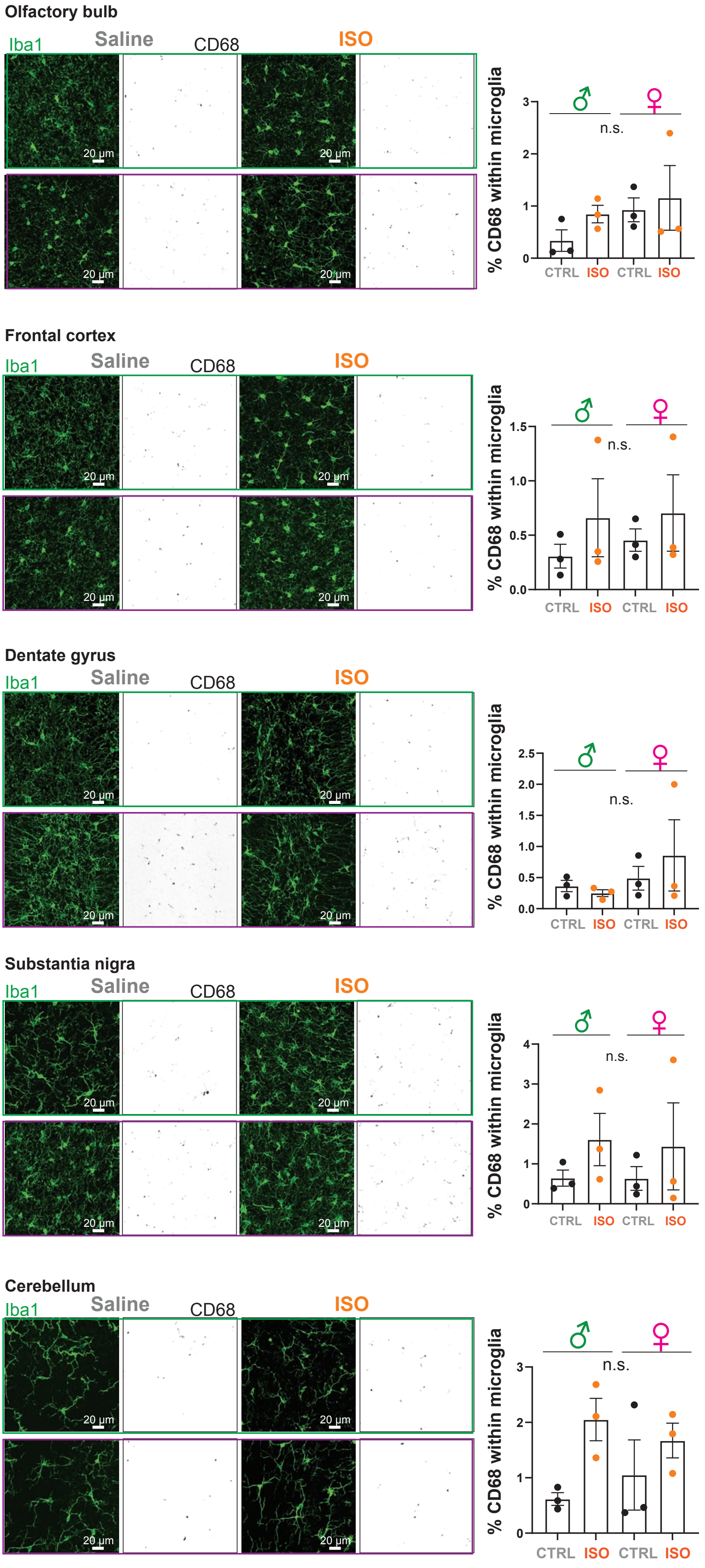

Figure S5. Microglial CD68 expression across multiple brain regions 4 hours after isoflurane anesthesia.

Representative immunostainings for microglia with Iba1 (green) and CD68 (black) in the olfactory bulb, frontal cortex, dentate gyrus, substantia nigra, and cerebellum of males and females, measured 4 hours after start of isoflurane inhalation. Control: wildtype animals. Scale bar: 20 µm. Bar charts of the mean percentage ± SEM of CD68 volume within microglia. Each dot, one animal. 3 animals/condition. Two-way ANOVA, $^{ns}p > 0.05$, not significant.

Figure S6

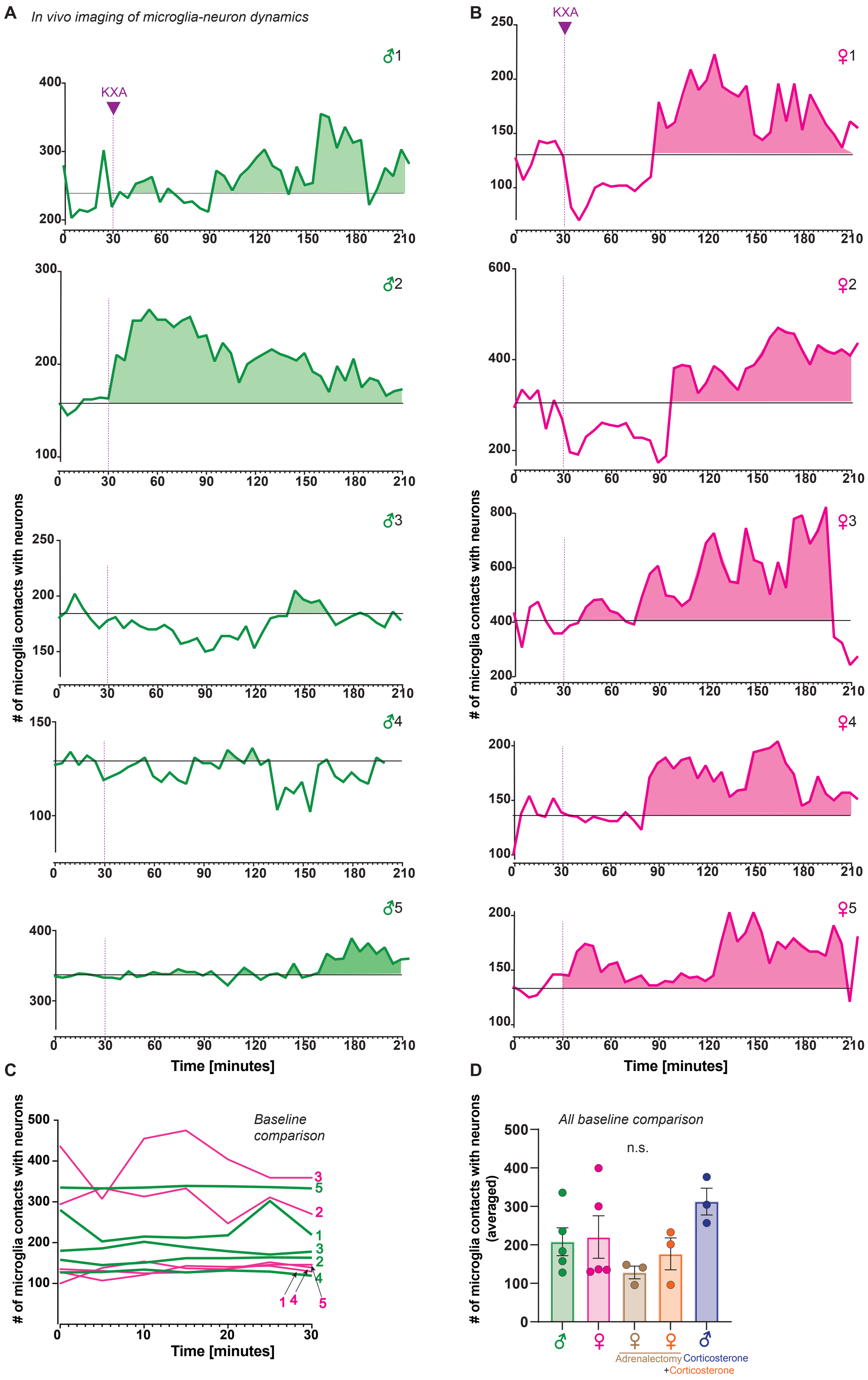

A
In vivo imaging of microglia-neuron dynamics
B
KXA
♂1
♂2
♂3
♂4
♂5
♀1
♀2
♀3
♀4
♀5
# of microglia contacts with neurons
Time [minutes]
C
Baseline comparison
D
All baseline comparison
n.s.
# of microglia contacts with neurons (averaged)
Adrenalectomy
+Corticosterone
Corticosterone

Figure S6. Not normalized data of individual animals of *in vivo* microglia-neurons interaction dynamics.

(**A-B**) Raw values of microglia-neuron interaction data obtained from long-term *in vivo* 2-photon imaging in the primary visual cortex (VISp) through a cranial window of Cx3cr1$^{CreERT2/-}$ ×Ai9×Thy1-EGFP mice of both sexes spanning the phases of awake, deep anesthesia, and recovery after KXA administration. Raw number of microglia and Thy1-EGFP neuronal process contacts over time in males (green, **A**) and in females (magenta, **B**). Positive area under the curve highlighted. Dashed violet line: KXA injection. Each plot is a different animal. (**C**) Comparison of the raw number of contacts between microglia and Thy1-EGFP neuronal process during baseline recordings (awake state) of males (**A**) and females (**B**). (**D**) Comparison of the averaged raw number of contacts between microglia and Thy1-EGFP neuronal processes during baseline recordings (awake state) of all the experimental conditions. Bar charts of the mean ± SEM of the microglia-Thy1-EGFP contacts. Each dot, one animal. 3-5 animals/condition. Two-way ANOVA, $^{ns}p > 0.05$, not significant.

Figure S7

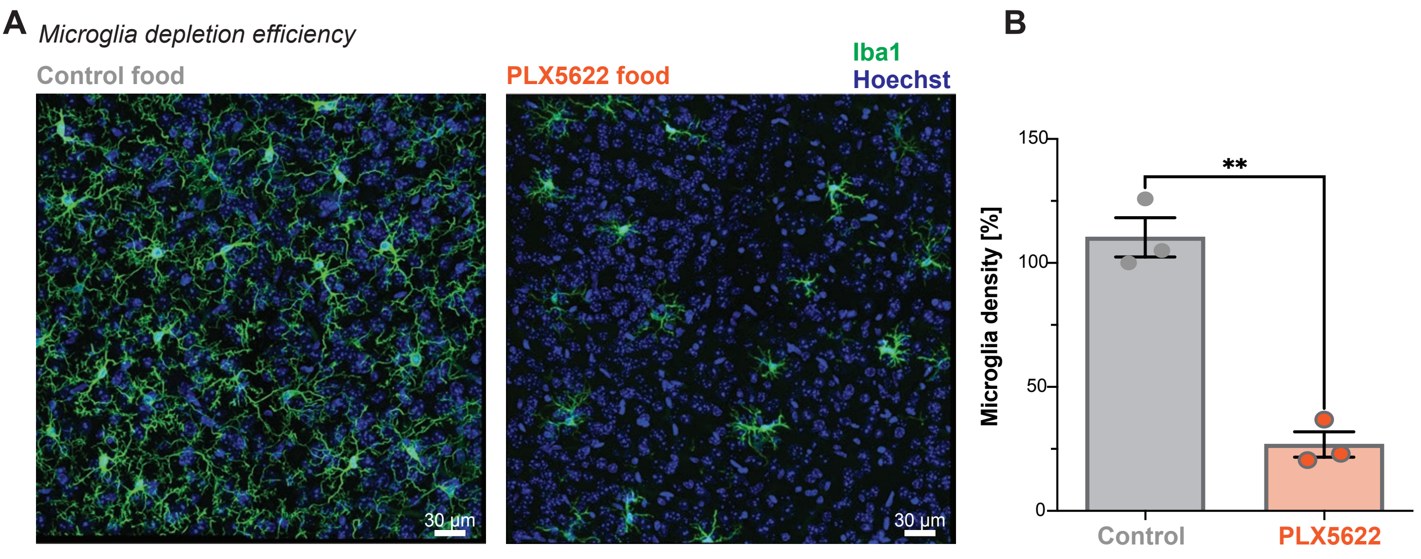

A
Microglia depletion efficiency
Control food
PLX5622 food
Iba1
Hoechst
30 µm
30 µm
B
Microglia density [%]
150
100
50
0
**
Control
PLX5622

Figure S7. Microglia depletion efficiency with PLX5622.

(**A**) Representative immunostainings for Iba1 (green), counterstained with the nuclei-dye Hoechst (blue). Left, control food. Right, feeding animals for 1.5 weeks with chow containing the Csf1-receptor inhibitor PLX5622. Scale bar: 30 µm. (**B**) Bar chart of mean percentage of microglia density in the VISp of saline and PLX5622-treated mice with ± SEM. Each dot, one animal. 3 animals/condition. Unpaired t-test with Welch's correction, $^{**}p < 0.01$.

Figure S8

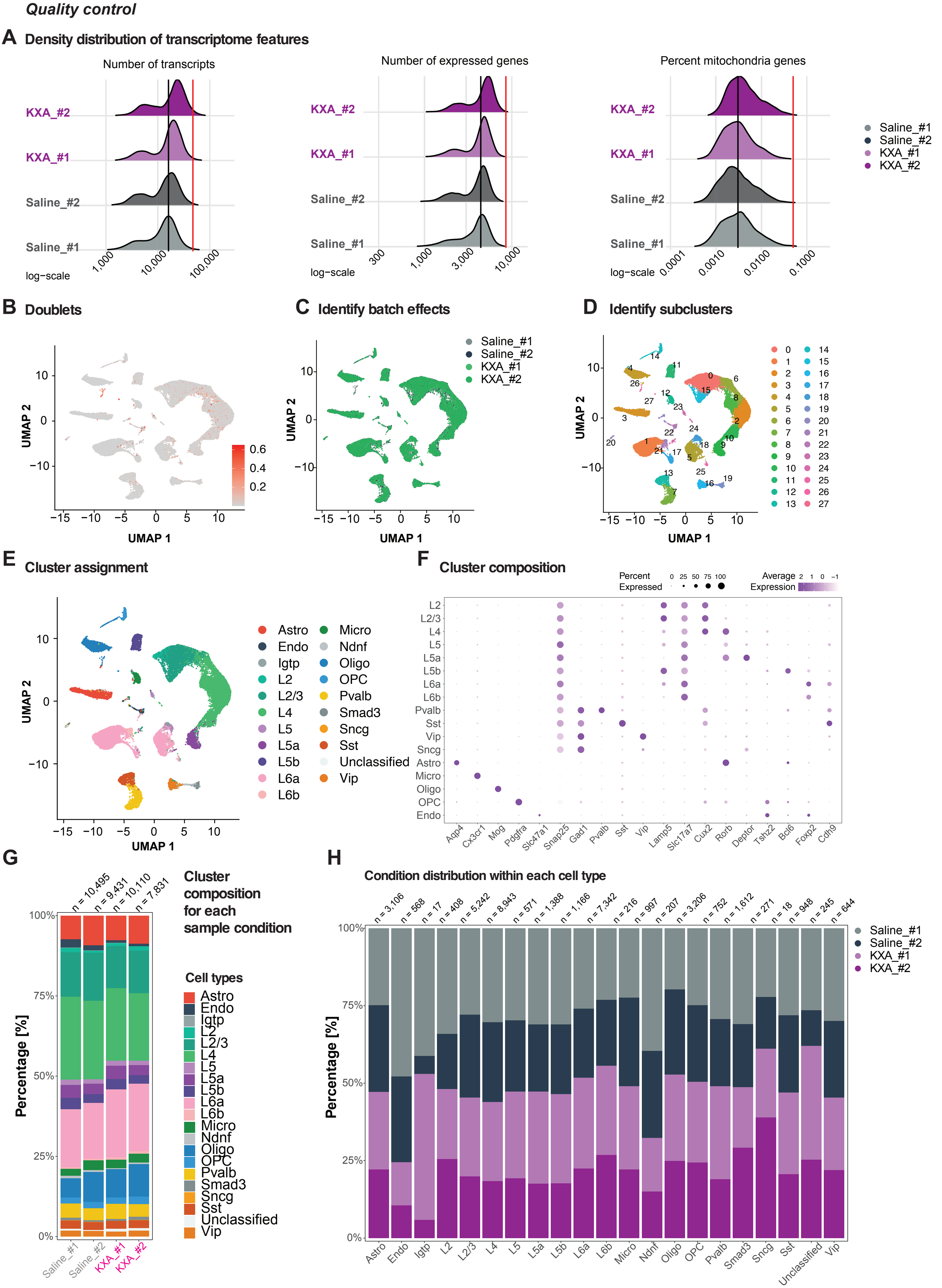

Quality control
A Density distribution of transcriptome features
Number of transcripts
Number of expressed genes
Percent mitochondria genes
KXA_#2
KXA_#1
Saline_#2
Saline_#1
log−scale
1,000
10,000
100,000
300
3,000
10,000
0.0001
0.0010
0.0100
0.1000
B Doublets
C Identify batch effects
D Identify subclusters
UMAP 1
UMAP 2
0.6
0.4
0.2
E Cluster assignment
Astro
Endo
Igtp
L2
L2/3
L4
L5
L5a
L5b
L6a
L6b
Micro
Ndnf
Oligo
OPC
Pvalb
Smad3
Sncg
Sst
Unclassified
Vip
F Cluster composition
Percent Expressed
0 25 50 75 100
Average Expression
2 1 0 −1
Aqp4
Cx3cr1
Mog
Pdgfra
Slc47a1
Snap25
Gad1
Pvalb
Sst
Vip
Lamp5
Slc17a7
Cux2
Rorb
Deptor
Tshz2
Bcl6
Foxp2
Cdh9
G
Cluster composition for each sample condition
Cell types
n = 10,495
n = 9,431
n = 10,110
n = 7,831
Percentage [%]
100%
75%
50%
25%
0%
H
Condition distribution within each cell type
n = 3,106
n = 568
n = 17
n = 408
n = 5,242
n = 8,943
n = 571
n = 1,388
n = 1,166
n = 7,342
n = 216
n = 997
n = 207
n = 3,206
n = 752
n = 1,612
n = 271
n = 18
n = 948
n = 245
n = 644

Figure S8. Quality control of single-nuclei multiome sequencing data.

(**A**-**H**) Multiome single-nuclei sequencing data from the entire primary visual cortex (VISp) of females, 2 hours after saline or KXA (ketamine-xylazine-acepromazine) injection. For each condition, two animals. Detailed description in the method section. (**A**) Density distribution plots of transcriptomic features focusing on the total number of transcripts (left), the number of expressed genes (middle), and the percentage of mitochondrial gene expression (right). Black line, median. Red line, threshold for filtering. (**B**) UMAP representation for doublets within the dataset. Doublets were filtered out. (**C**) UMAP plot after log-normalization of the filtered count matrix. (**D**-**E**) UMAP of batch corrected representation and assignment of subclusters to cell types. (**F**) Verification of cell-type-specific signature genes in the corresponding cluster. (**G**-**H**) Distribution plots for the percentage of cell type populations within a condition (**G**) and the percentage of conditions within a cell type population (**H**). Absolute cell numbers are indicated above each bar.

Figure S9

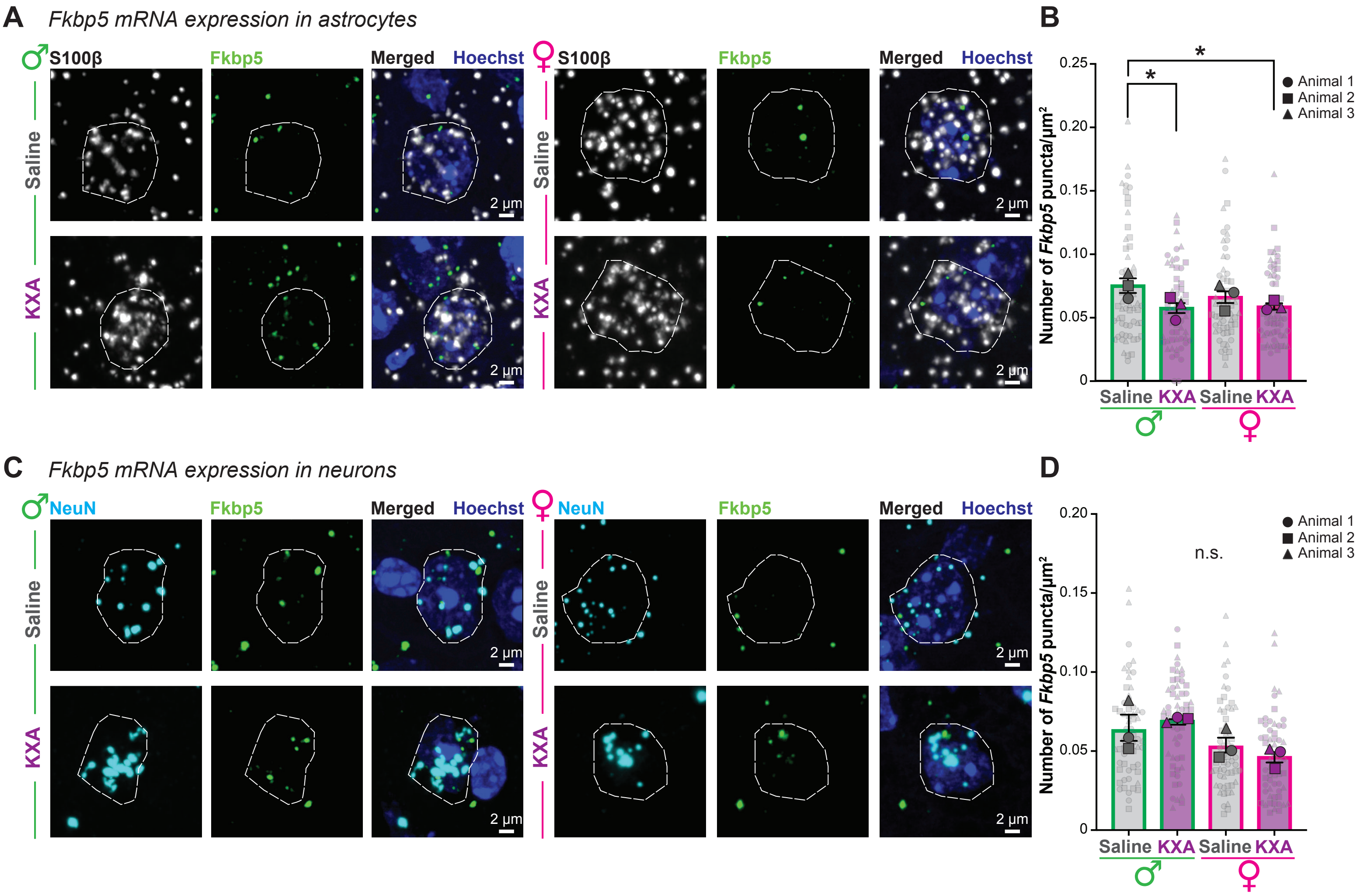

A
Fkbp5 mRNA expression in astrocytes
S100β
Fkbp5
Merged
Hoechst
Saline
KXA
2 µm
B
Number of Fkbp5 puncta/µm²
0.25
0.20
0.15
0.10
0.05
0
Animal 1
Animal 2
Animal 3
*
*
Saline
KXA
Saline
KXA
C
Fkbp5 mRNA expression in neurons
NeuN
Fkbp5
Merged
Hoechst
Saline
KXA
2 µm
D
Number of Fkbp5 puncta/µm²
0.20
0.15
0.10
0.05
0
Animal 1
Animal 2
Animal 3
n.s.
Saline
KXA
Saline
KXA

Figure S9. Astrocytes and neurons do not upregulate *Fkbp5* mRNA expression during recovery from ketamine anesthesia.

(**A**-**D**) Fluorescence *in situ* hybridization for male and female mice 2 hours after saline or KXA injection in VISp for mRNA probes against *Fkbp5* (green), *S100β* (white) for astrocytes (**A**-**B**), and *NeuN* (cyan) for neurons (**C**-**D**), counterstained with the nuclei-dye Hoechst (blue), which provides the nucleus contour (white dashed line). Scale bar: 2 µm. (**B**, **D**) Bar chart of the mean *Fkbp5* mRNA puncta within the Hoechst contour with ± SEM. Each dot represents one contour, 20 cells per animal, 3 animals/condition. One-way nested ANOVA with selected Tukey's multiple comparisons post hoc test, $^{*}p < 0.05$, $^{ns}p > 0.05$, not significant.

Figure S10

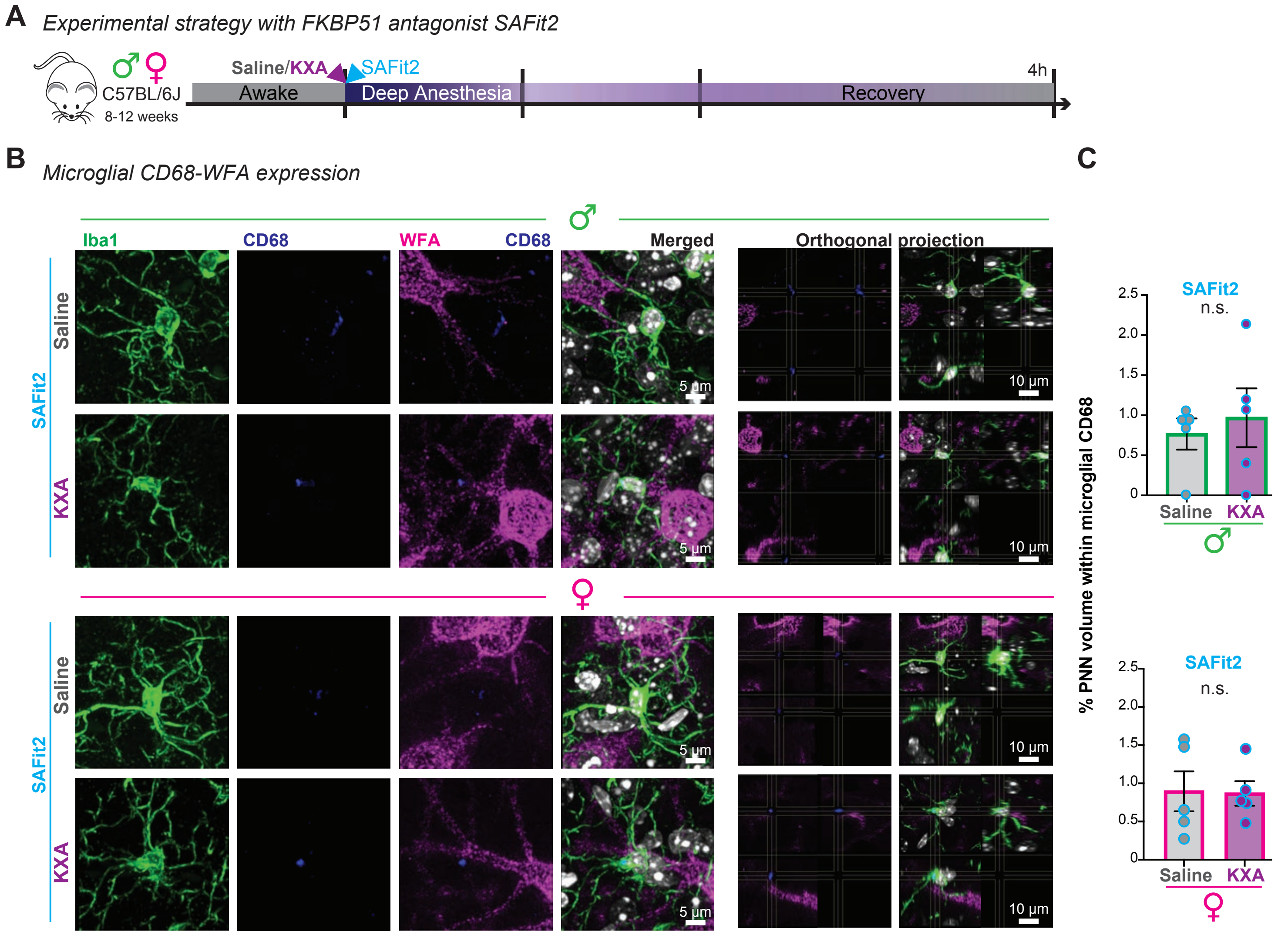

A
Experimental strategy with FKBP51 antagonist SAFit2
♂ ♀
C57BL/6J
8-12 weeks
Saline/KXA
SAFit2
4h
Awake
Deep Anesthesia
Recovery
B
Microglial CD68-WFA expression
♂
Iba1
CD68
WFA
CD68
Merged
Orthogonal projection
SAFit2
Saline
KXA
5 µm
10 µm
♀
C
% PNN volume within microglial CD68
SAFit2
n.s.
2.5
2.0
1.5
1.0
0.5
0
Saline
KXA

Figure S10. SAFit2 treatment inhibits female microglial remodeling of perineuronal nets (PNN).

(**A**-**C**) Antagonising FKBP51 with SAFit2 after injection of saline or KXA (ketamine-xylazine-acepromazine). (**B**-**C**) Comparison of WFA staining inside microglia CD68. (**B**) Representative images of immunostained microglia with Iba1 (green), CD68 (blue), *Wisteria floribunda agglutinin* (WFA, magenta) for perineuronal nets (PNN), and counterstained with the nuclei-dye Hoechst (white) in the primary visual cortex (VISp), layer III-V of males and females, 4 hours after saline or KXA injection. Scale bar: 5 µm. Next to the merged image, orthogonal projections. Scale bar: 10 µm. (**C**) Bar charts of the mean percentage of PNN volume within microglial CD68 between males (green, top) and females (magenta, bottom) with ± SEM. Each dot, one animal. 5 animals/condition. Males (top bar chart), Unpaired t-test with Welch's correction. Mann-Whitney test and Unpaired t-test with Welch's correction. Females (bottom bar chart), Unpaired t-test with Welch's correction. Mann-Whitney test and Unpaired t-test with Welch's correction. $^{ns}p > 0.05$, not significant.

Figure S11

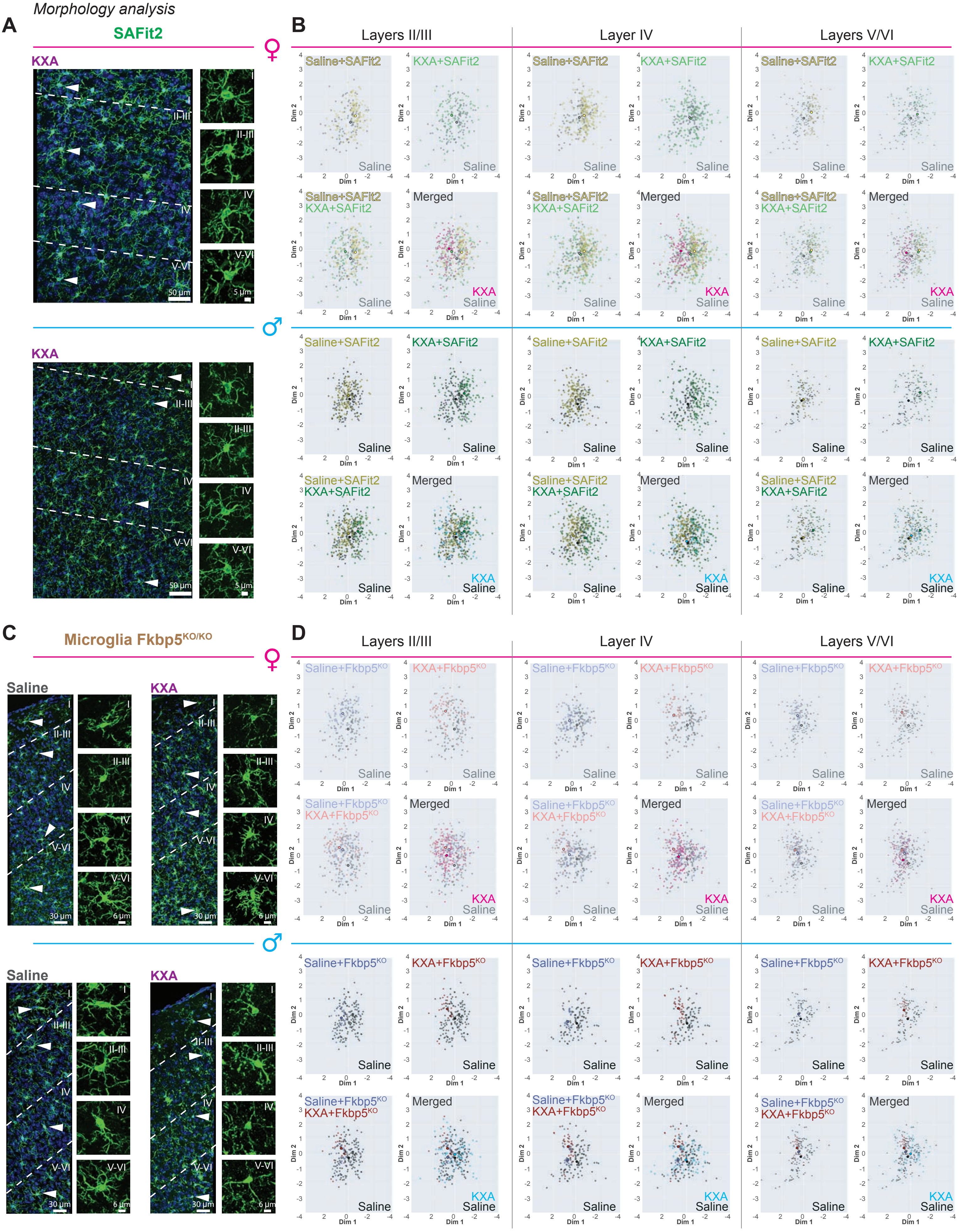

A
Morphology analysis
SAFit2
KXA
B
Layers II/III
Layer IV
Layers V/VI
Saline+SAFit2
KXA+SAFit2
Merged
Saline
KXA
Dim 1
Dim 2
C
Microglia Fkbp5KO/KO
Saline
KXA
D
Saline+Fkbp5KO
KXA+Fkbp5KO
I
II-III
IV
V-VI
50 µm
5 µm
30 µm
6 µm

Figure S11. Morphological analysis of microglia across VISp layers after SAFit2 or microglia-specific *Fkbp5*^{KO}.

Morphological analysis of microglia after saline or KXA (ketamine-xylazine-acepromazine) with (**A**-**B**) antagonising FKBP51 with SAFit2 after injection in C57BL/6J or (**C**-**D**) in microglia-selective *Fkbp5*-knockout experiment using a tamoxifen-inducible Cx3cr1$^{CreERT2/-}$ crossed with *Fkbp5*$^{KO/KO}$ reporter mouse line (Microglia Fkbp5$^{KO/KO}$). 3 consecutive tamoxifen injections, starting 8 days before performing the procedure. (**A**, **C**) Representative immunostainings for Iba1 (green) and CD68 (magenta) counterstained with the nuclei-dye Hoechst (blue) in the primary visual cortex (VISp) of females (magenta) and males (cyan). Left, overview image of the cortical layers. Scale bar: 50 µm (**A**), 30 µm (**C**). Arrow in each layer, microglia chosen for zoom-in. Scale bar: 5 µm. (**B**, **D**) Morphological analysis of SAFit2 (**B**) and microglia *Fkbp5*$^{KO/KO}$ (**D**) microglia morphology within cortical layer II/III, IV, and V/VI in VISp after saline (grey, black) or KXA (magenta, cyan) using morphOMICs. Each microglia's persistence image was embedded in a latent space using a Variational Autoencoder (VAE, see Methods). Larger dots, population mean for sex and condition.

Figure S12

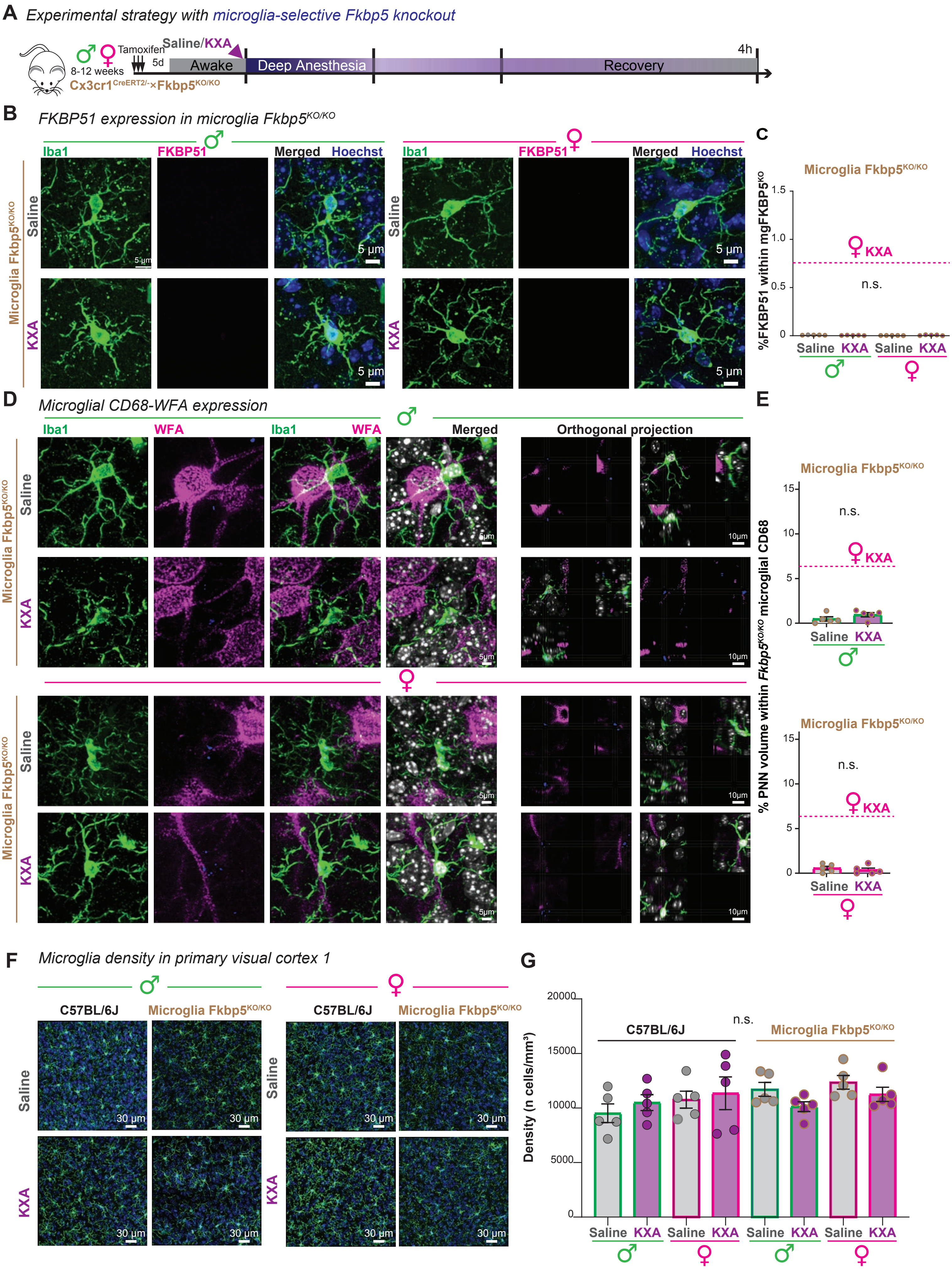
A Experimental strategy with microglia-selective Fkbp5 knockout
Tamoxifen
Saline/KXA
8-12 weeks
5d
Awake
Deep Anesthesia
Recovery
4h
Cx3cr1CreERT2/-×Fkbp5KO/KO
B FKBP51 expression in microglia Fkbp5KO/KO
Iba1
FKBP51
Merged
Hoechst
Saline
KXA
Microglia Fkbp5KO/KO
5 µm
C
Microglia Fkbp5KO/KO
%FKBP51 within mgFKBP5KO
KXA
n.s.
Saline
KXA
D Microglial CD68-WFA expression
Iba1
WFA
Merged
Orthogonal projection
5µm
10µm
E
% PNN volume within Fkbp5KO/KO microglial CD68
n.s.
F Microglia density in primary visual cortex 1
C57BL/6J
Microglia Fkbp5KO/KO
30 µm
G
Density (n cells/mm³)
n.s.
C57BL/6J
Microglia Fkbp5KO/KO
Saline
KXA

Figure S12. Microglia *Fkbp5*^{KO} prevents FKBP51 upregulation and PNN remodeling, without affecting microglia numbers.

(**A**-**G**) Consequences of microglia-selective *Fkbp5*-knockout experiment in the primary visual cortex (VISp), layer III-V of males and females, 4 hours after saline or KXA injection using a tamoxifen-inducible Cx3cr1$^{CreERT2/-}$ ×Fkbp5$^{KO/KO}$ reporter mouse line (Microglia Fkbp5$^{KO/KO}$). 3 consecutive tamoxifen injections, starting 8 days before performing the procedure (**A**). (**B**-**C**) Confirmation of FKBP51 knockdown. (**B**) Immunostaining for FKBP51 protein expression (magenta) in microglia (Iba1, green), counterstained with the nuclei-dye Hoechst (blue). Scale bar: 5 µm. (**C**) Bar chart of the mean percentage of FKBP51 volume within microglia with ± SEM. Each dot, one animal, 5 animals/condition. Magenta line, reference value of female KXA for FKBP51 (**Figure 2G**). Two-way ANOVA. $^{ns}p > 0.05$, not significant. (**D**-**E**) Comparison of perineuronal nets staining inside microglia CD68 between males (green) and females (magenta). (**D**) Representative images of immunostained microglia with Iba1 (green), CD68 (blue), *Wisteria floribunda agglutinin* (WFA, magenta) for perineuronal nets (PNN), and counterstained with the nuclei-dye Hoechst (white) in the primary visual cortex (VISp), layer III-V of males and females. Scale bar: 5 µm. Next to the merged image, orthogonal projections. Scale bar: 10 µm. (**E**) Bar charts of the mean percentage of PNN volume within microglial CD68. Each dot, one animal. 5 animals/condition. Magenta line, reference value of female KXA for PNN within microglia CD68 (**Figures S2D**, **S2F**). Two-way ANOVA. $^{ns}p > 0.05$, not significant. (**F**-**G**) Microglia density. (**F**) Immunostaining for Iba1 (green), counterstained with the nuclei-dye Hoechst (blue). Scale bar: 30 µm. (**G**) Bar chart of the mean microglia density in the primary visual cortex (VISp), layer II-V, across conditions. Each dot, one animal. 5 animals/condition. Kruskal-Wallis test. $^{ns}p > 0.05$, not significant.

Figure S13

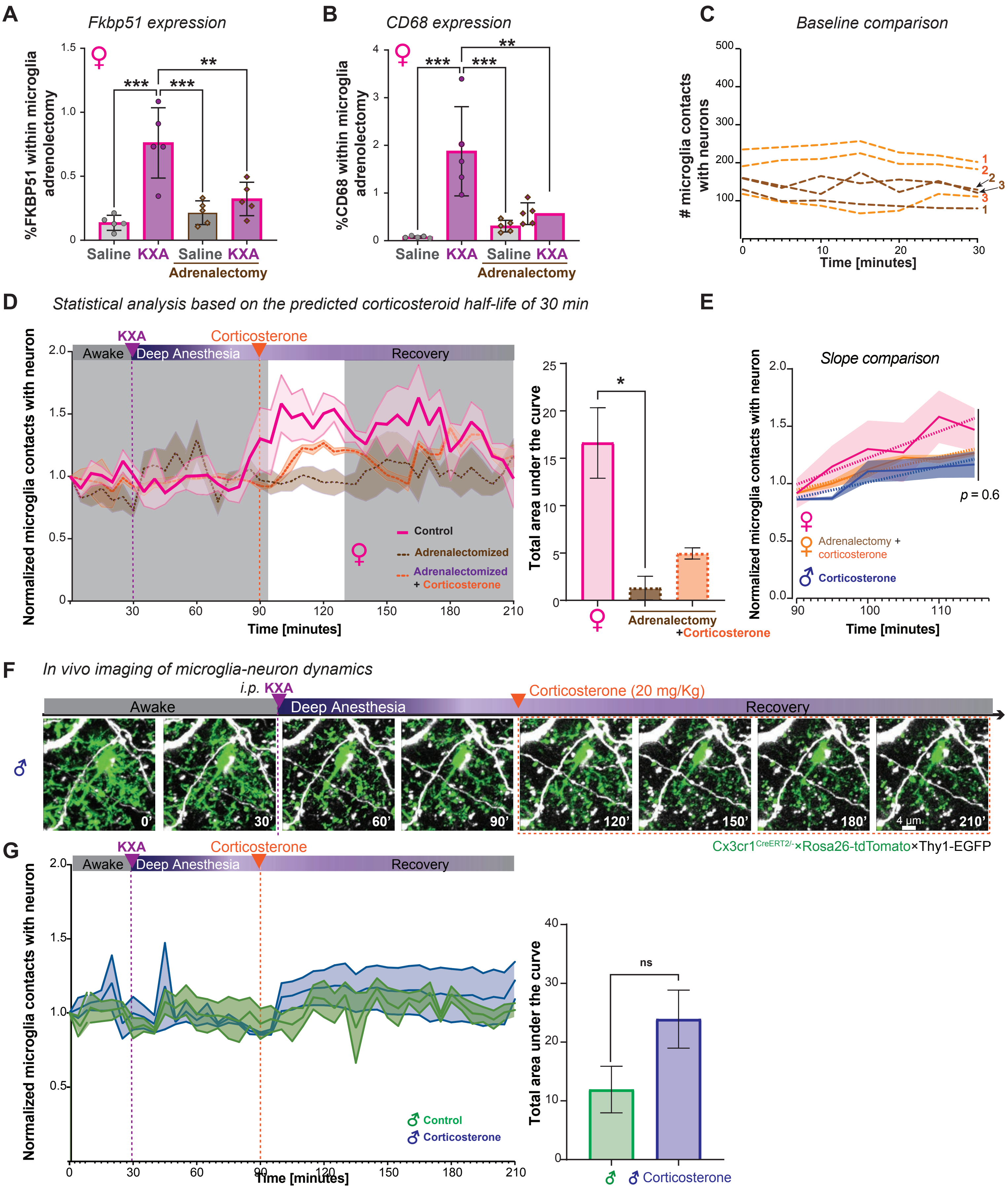
A
Fkbp51 expression
%FKBP51 within microglia adrenolectomy
***
**
Saline
KXA
Adrenalectomy
B
CD68 expression
%CD68 within microglia adrenolectomy
C
Baseline comparison
microglia contacts with neurons
Time [minutes]
D
Statistical analysis based on the predicted corticosteroid half-life of 30 min
KXA
Corticosterone
Awake
Deep Anesthesia
Recovery
Normalized microglia contacts with neuron
Time [minutes]
Control
Adrenalectomized
Adrenalectomized + Corticosterone
Total area under the curve
*
Adrenalectomy
+Corticosterone
E
Slope comparison
p = 0.6
Adrenalectomy + corticosterone
Corticosterone
F
In vivo imaging of microglia-neuron dynamics
i.p. KXA
Corticosterone (20 mg/Kg)
0'
30'
60'
90'
120'
150'
180'
210'
4 µm
Cx3cr1CreERT2/-×Rosa26-tdTomato×Thy1-EGFP
G
Control
Corticosterone
ns

Figure S13. Systemic administration of corticosterone alters microglia-neuron interaction dynamics in males and adrenalectomized females.

(**A**-**B**) Bar chart of the mean percentage of FKBP51 (**A**) and CD68 (**B**) volume within adrenalectomized female microglia with ± SEM. Each dot, one animal, 5 animals/condition. Two-way ANOVA with selected Tukey's multiple comparisons post hoc test, $^{**}p < 0.01$, $^{***}p < 0.001$. (**C**) Comparison of the raw number of contacts between microglia and Thy1-EGFP neuronal process during baseline recordings (awake state) of adrenalectomized females (dashed brown lines) and adrenalectomized females that will be injected with corticosterone (dashed orange line). (**D**) Normalized number of microglia and Thy1-EGFP neuronal process contacts over time in females (magenta, see **Figure 1B**), adrenalectomized females (purple, n=3), and adrenalectomized females injected with corticosterone 60 minutes after KXA injection (orange, n=3) represented as mean ± SEM confidence band. Dashed lines: KXA (violet) and corticosterone (orange) injections. White band, area used for statistical analysis based on predicted corticosteroid half-life in rodents. Next, bar chart of the mean total area under the curves in (**C**) (magenta female from **Figure 1C**). Mean ± SEM of 3 - 5 animals per condition. Brown-Forsythe ANOVA test with Dunnett's T3 multiple comparisons test, $^{*}p < 0.05$. (**E**) Slope measurement calculated between 90 and 115 minutes of recording of the Normalized number of microglia and Thy1-EGFP neuronal process contacts represented as mean ± SEM confidence band. Magenta, females, orange, adrenalectomized females after corticosterone injection, blue, males after corticosterone injection. Slopes were compared using linear regression analysis and the Extra sum-of-squares F test, $^{ns}p > 0.05$, not significant. (**F**) Sequential snapshots of microglia (green) and excitatory projection neurons (white) of males. Dashed violet line: KXA injection, dashed orange line: corticosterone injection. 3 animals per condition. Scale bar: 4 µm. (**G**) Normalized number of microglia and Thy1-EGFP neuronal process contacts over time in males (green) and males injected with corticosterone (blue) represented as mean ± SEM confidence band. Dashed violet line: KXA injection, dashed orange line: corticosterone injection. 3-5 animals per condition. Next, a bar chart of the mean total area under the curves. Mean ± SEM of 3-5 animals per condition. Unpaired t-test with Welch's correction, $^{ns}p > 0.05$, not significant.

Figure S14

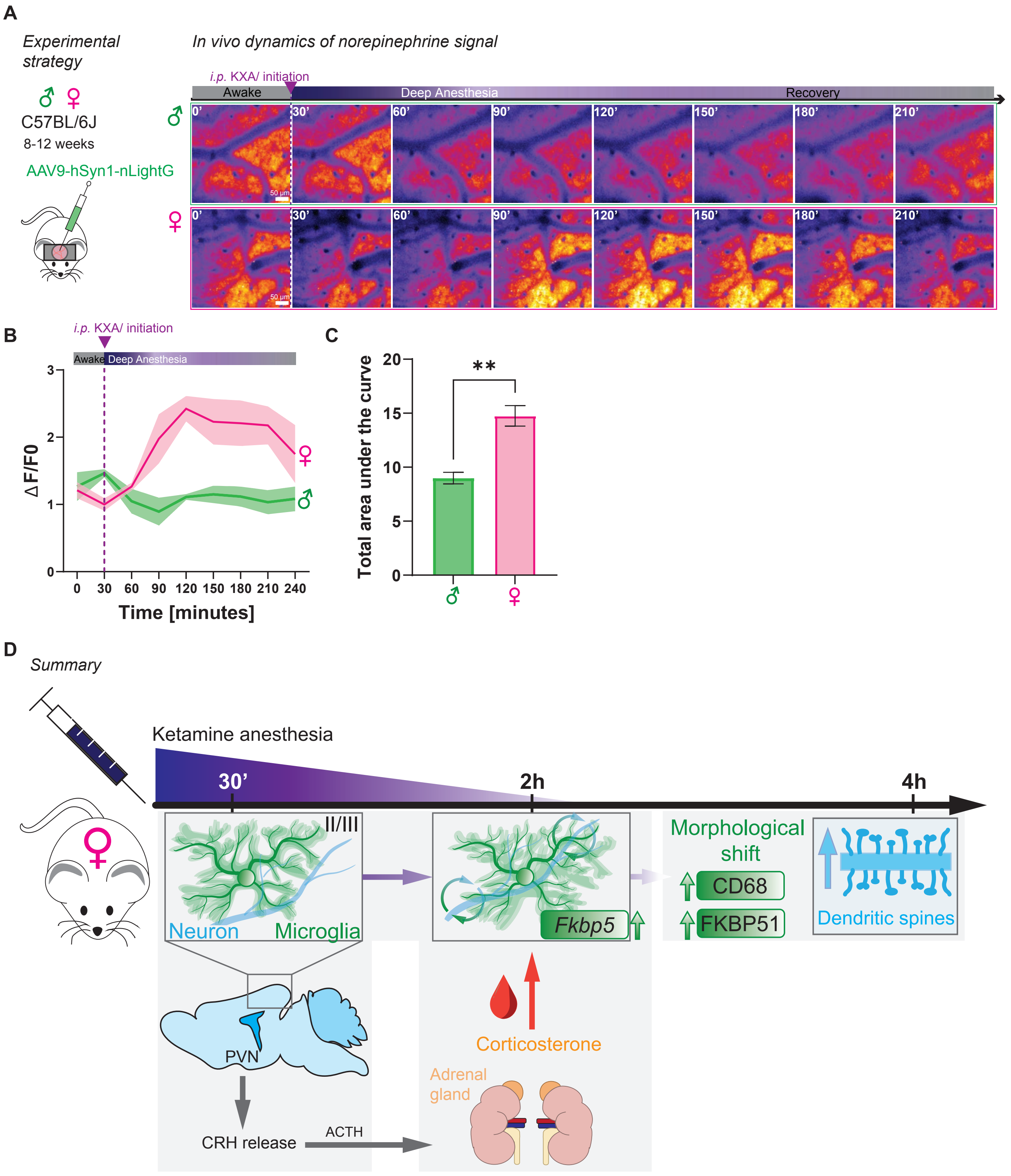
A
Experimental strategy
♂ ♀
C57BL/6J
8-12 weeks
AAV9-hSyn1-nLightG
In vivo dynamics of norepinephrine signal
i.p. KXA/ initiation
Awake
Deep Anesthesia
Recovery
0' 30' 60' 90' 120' 150' 180' 210'
50 µm
B
i.p. KXA/ initiation
Awake
Deep Anesthesia
ΔF/F0
Time [minutes]
0 30 60 90 120 150 180 210 240
C
Total area under the curve
**
D
Summary
Ketamine anesthesia
30'
2h
4h
II/III
Neuron
Microglia
Fkbp5
Morphological shift
CD68
FKBP51
Dendritic spines
PVN
CRH release
ACTH
Corticosterone
Adrenal gland

Figure S14.

(**A**-**C**) Effect of KXA anesthesia on norepinephrine dynamics measured in vivo in C57BL/6J mice of both sexes with intracranial injection of AAV9-hSyn1-nLightG virus during cranial window implant. (**A**) Sequential snapshots of norepinephrine fluorescence intensity dynamics (LUT fire) of males and females. Dashed violet line: KXA injection. 3 animals per condition. Scale bar: 50 μm. (**G**) Fluorescence intensity dynamics (ΔF/F0) of the norepinephrine signal over time in males (green) and females (magenta) during awake, deep anesthesia, and recovery from KXA, represented as mean ± SEM confidence band. Dashed violet line: KXA injection. (**C**) Bar chart of the mean total area under the curves in (**B**). Mean ± SEM of 3 animals per condition. Unpaired t-test, ***p* < 0.01. **(D)** Schematic overview of the effects of ketamine anesthesia during the recovery on microglia (green) and neurons (blue). II/III, cortical layer II and III. ACTH, adrenocorticotropic hormone. CRH, corticosterone-releasing hormone. PVN, paraventricular nucleus.

Video S1. *In vivo* two-photon time-lapse imaging of microglia-neuron interaction dynamics before and after KXA injection in the VISp of $Cx3cr1^{CreERT2/-}$×Ai9×Thy1-EGFP males (same view as Figure 1A). Microglia (green) interact with excitatory projection neurons (white) at baseline, deep anesthesia, and the recovery phase.

Video S2. *In vivo* two-photon time-lapse imaging of microglia-neuron interaction dynamics before and after KXA injection in the VISp of $Cx3cr1^{CreERT2/-}$×Ai9×Thy1-EGFP females (same view as Figure 1A). Microglia (green) interact with excitatory projection neurons (white) at baseline, deep anesthesia, and the recovery phase. During the recovery phase, female microglia show long-lasting contacts with excitatory dendrites.

Video S3. *In vivo* two-photon time-lapse imaging of microglia-neuron interaction dynamics before and after KXA injection in the VISp of adrenalectomized $Cx3cr1^{CreERT2/-}$×Ai9×Thy1-EGFP females (same view as Figure 4I). Microglia (green) interact with excitatory projection neurons (white) at baseline, deep anesthesia, and the recovery phase.

Video S4. *In vivo* two-photon time-lapse imaging of microglia-neuron interaction dynamics before and after KXA injection in the VISp of adrenalectomized $Cx3cr1^{CreERT2/-}$×Ai9×Thy1-EGFP females injected *i.p.* with corticosterone (same view as Figure 4I). Microglia (green) interact with excitatory projection neurons (white) at baseline, during deep anesthesia, and after corticosterone injection 1 hour after KXA.

Supplementary Table 1. Summary of data and statistical analyses, related to Figures 1-4 and Figures S1-S14.
